# Structure-based computational prioritization of Emodin-8-glucoside as a candidate ligand for the VEGFR1 kinase domain in the context of placenta previa

**DOI:** 10.1371/journal.pone.0345201

**Published:** 2026-10-15

**Authors:** Deniz Inan, Sinan Karageçili, Nouman Ali

**Affiliations:** 1 Özel Nev Hospital, Sanlıurfa, Türkiye; 2 Department of Molecular Biosciences, Faculty of Physical and Biological Sciences, Rashid Latif Khan University, Lahore, Punjab, Pakistan; Kwara State University, NIGERIA

## Abstract

Placenta previa is a serious obstetric condition associated with abnormal placental implantation and hemorrhagic complications, for which no targeted pharmacological therapy is established. This study applied an integrated structure-based computational workflow to explore the intracellular VEGFR1 kinase domain as a candidate molecular target. Twenty-one phytochemicals were screened using AutoDock Vina and MOE, with Emodin-8-glucoside consistently prioritized among the highest-ranked compounds. Redocking of the co-crystallized ligand 8ST reproduced its experimental pose with a heavy-atom RMSD of 1.465 Å. Comparative 500 ns molecular dynamics simulations showed persistent binding-site association of Emodin-8-glucoside and the 8ST control. End-point energy estimates were method dependent, with MM-GBSA yielding a negative mean estimate while MM-PBSA yielded a near-zero estimate with substantial variability. DFT analysis produced a HOMO-LUMO gap of 0.13204 Hartree (approximately 3.59 eV). SwissADME and ProTox 3.0 identified low gastrointestinal absorption, P-glycoprotein substrate behavior, high polarity, and several predicted toxicity liabilities. Overall, Emodin-8-glucoside represents an in-silico-prioritized exploratory candidate requiring biochemical, selectivity, placental-cell, and pregnancy-safety validation.

## Introduction

Placenta previa is an obstetric complication which occurs when the placenta adheres to the lower uterine segment, thereby partially or completely obstructing the cervical os [1–3]. This unusual placentation is a serious risk, often leading to widespread maternal and fetal morbidity due to the antenatal hemorrhage [1,4]. Clinically, placenta previa usually presents with sudden, painless vaginal bleeding during the second half of gestation, which may be repeated and, in some cases, may lead to massive and life-threatening hemorrhage [5]. Indeed, bleeding associated with placenta previa is a leading cause of maternal mortality worldwide and may require blood transfusion, emergency hysterectomy, or intensive care admission and, in severe cases, results in maternal death [6,7]. There is no pharmacological treatment that has been shown to resolve placenta previa, therefore modern management is mainly supportive and includes pelvic rest, hemodynamic stabilization, and corticosteroid treatment to hasten fetal lung maturity, with definitive treatment of caesarean delivery when fetal maturity is achieved or when hemorrhage justifies surgical treatment [7]. This lack of a therapeutic modality that can prevent or ameliorate this disease highlights the urgent need for innovative approaches that focus on the pathophysiologic mechanisms of implantation.

Vascular endothelial growth factor receptor 1 (VEGFR1), encoded by the FLT1 gene, occurs as a full-length membrane-bound receptor and as soluble splice variants collectively referred to as soluble fms-like tyrosine kinase 1 (sFlt-1). Full-length VEGFR1 contains extracellular ligand-binding, transmembrane, and intracellular tyrosine-kinase regions. Binding of VEGF-A or placental growth factor (PlGF) to the extracellular region can initiate phosphorylation-dependent signaling through the intracellular kinase domain in responsive cells. By contrast, sFlt-1 retains the extracellular ligand-binding region but lacks the transmembrane and intracellular kinase domains, enabling it to function as a soluble decoy receptor that sequesters VEGF-A and PlGF [8,9]. Consequently, a small molecule predicted to occupy the intracellular ATP-binding pocket could be hypothesized to influence the kinase activity of membrane-bound VEGFR1, but it would not directly alter the binding or sequestration of VEGF-A or PlGF by sFlt-1.

Placenta previa, placenta accreta spectrum (PAS), preeclampsia, and fetal growth restriction (also termed intrauterine growth restriction in some cited studies) are clinically and biologically distinct conditions. Placenta previa is defined by placental implantation over or near the internal cervical os, whereas PAS is characterized by abnormal placental adherence or invasion into the uterine wall and may coexist with placenta previa. Preeclampsia is a hypertensive multisystem disorder frequently associated with an antiangiogenic circulating profile, while fetal growth restriction is a heterogeneous fetal-growth outcome commonly associated with placental insufficiency. Therefore, observations concerning FLT1 or sFlt-1 expression in preeclampsia and fetal growth restriction provide broader information about placental angiogenic biology but cannot be assumed to represent the pathogenesis of placenta previa [9]. Similarly, biomarker findings obtained from patients with “pernicious” placenta previa accompanied by placenta accreta or increta should not be generalized to uncomplicated placenta previa [1]. Placenta-previa tissue studies have reported altered VEGF expression, inflammatory signaling, and vascular morphology, providing disease-specific evidence that angiogenic regulation may be disturbed in this condition [10]. However, these studies did not demonstrate increased kinase activity of membrane-bound VEGFR1 or establish that pharmacological VEGFR1 inhibition would be beneficial. VEGFR1 was therefore examined in the present study as a hypothesis-generating structural target within a broader angiogenic context rather than as an established therapeutic target for placenta previa.

Natural products provide chemically diverse scaffolds that have contributed substantially to drug discovery. Secondary metabolites, including alkaloids, flavonoids, quinones, and terpenoids, therefore represent useful starting points for structure-based virtual screening [11–13]. However, natural origin, dietary exposure, or traditional use does not independently establish pharmacological efficacy or maternal–fetal safety. In the present study, phytochemicals were treated as a chemically diverse screening library rather than as established obstetric therapeutics. Emodin-8-glucoside is a hydroxyanthraquinone glycoside reported in several medicinal plant species, including rhubarb and Aloe species, and has been investigated in non-obstetric biological models [14–17]. Because its activity against VEGFR1 and its pregnancy-specific safety have not been established, Emodin-8-glucoside was evaluated only as a candidate for computational prioritization.

Emodin-8-glucoside and structurally related anthraquinones have been investigated in several non-placental experimental systems. Reported observations include effects on cell-cycle regulation in cancer models and modulation of macrophage-associated signaling pathways [17,18]. The aglycone emodin has also been reported to influence angiogenesis-related processes, including VEGFR2 phosphorylation, matrix metalloproteinase expression, and inflammatory signaling in experimental cancer or inflammatory models [19,20]. These findings concern different molecular forms, biological systems, and disease contexts and therefore cannot be extrapolated directly to VEGFR1 inhibition by Emodin-8-glucoside or to biological activity in placenta previa or PAS. They provide a rationale for including Emodin-8-glucoside in an exploratory structure-based screen but do not establish therapeutic efficacy, pregnancy safety, or modulation of placental angiogenesis.

To extract these benefits in a rationalized way, a structure-based drug design (SBDD) approach is justified. Recent progress in computer-aided drug design (CADD) has allowed researchers to rationalize *in-silico* the design and optimization of drug candidates, which allows for rapid lead discovery at substantially reduced cost [21,22]. SBDD takes advantage of high-resolution three-dimensional structures of target proteins to rationalize the potential binding mode and modulation of those targets by small molecules [23]. This method has become a major driver for modern medicinal chemistry, especially in view of the recent explosion of structural data from X-ray crystallography, cryo-electron microscopy, and *in-silico* methods for structure prediction (AlphaFold 3.0) [23,24]. By virtually screening large small molecule libraries and using docking algorithms, promising hit molecules that bind into the active/potentially allosteric site(s) of the protein can be identified without requiring purified protein. The hits are then optimized via iterative design cycles and molecular dynamics simulations to assess the stability of ligand-protein interactions and to take into account receptor flexibility [23,25]. *In-silico* modeling is particularly useful for diseases such as placenta previa, for which traditional drug screening is constrained by ethical and logistical considerations in pregnant patients. Thus, CADD approaches provide a time- and cost-efficient approach for examining potential therapeutics in a virtual environment before laboratory or clinical testing for placenta previa.

In this study, an integrated structure-based computational workflow was applied to assess whether selected phytochemicals could adopt predicted binding poses within the ATP-binding site of the intracellular VEGFR1 kinase domain. Molecular docking, molecular dynamics simulation, end-point binding-energy calculations, and in silico molecular-property analyses were used to prioritize candidate ligands for subsequent experimental investigation. Because the modeled ATP-binding site is present in full-length membrane-bound VEGFR1 but absent from soluble sFlt-1, the results relate specifically to predicted kinase-domain binding and do not demonstrate direct modulation of PlGF sequestration by sFlt-1. Placenta previa provides the exploratory disease context for this investigation; however, the computational analysis does not establish increased VEGFR1 kinase activity in placenta previa, therapeutic benefit from its inhibition, or the maternal–fetal safety of the screened compounds. Emodin-8-glucoside is therefore presented as a computationally prioritized candidate requiring biochemical kinase assays, cellular target-engagement studies, and validation in appropriate placental models.

## Methodology

The overall methodology flow chart of the present study can be seen in Fig 1.

**Fig 1:**
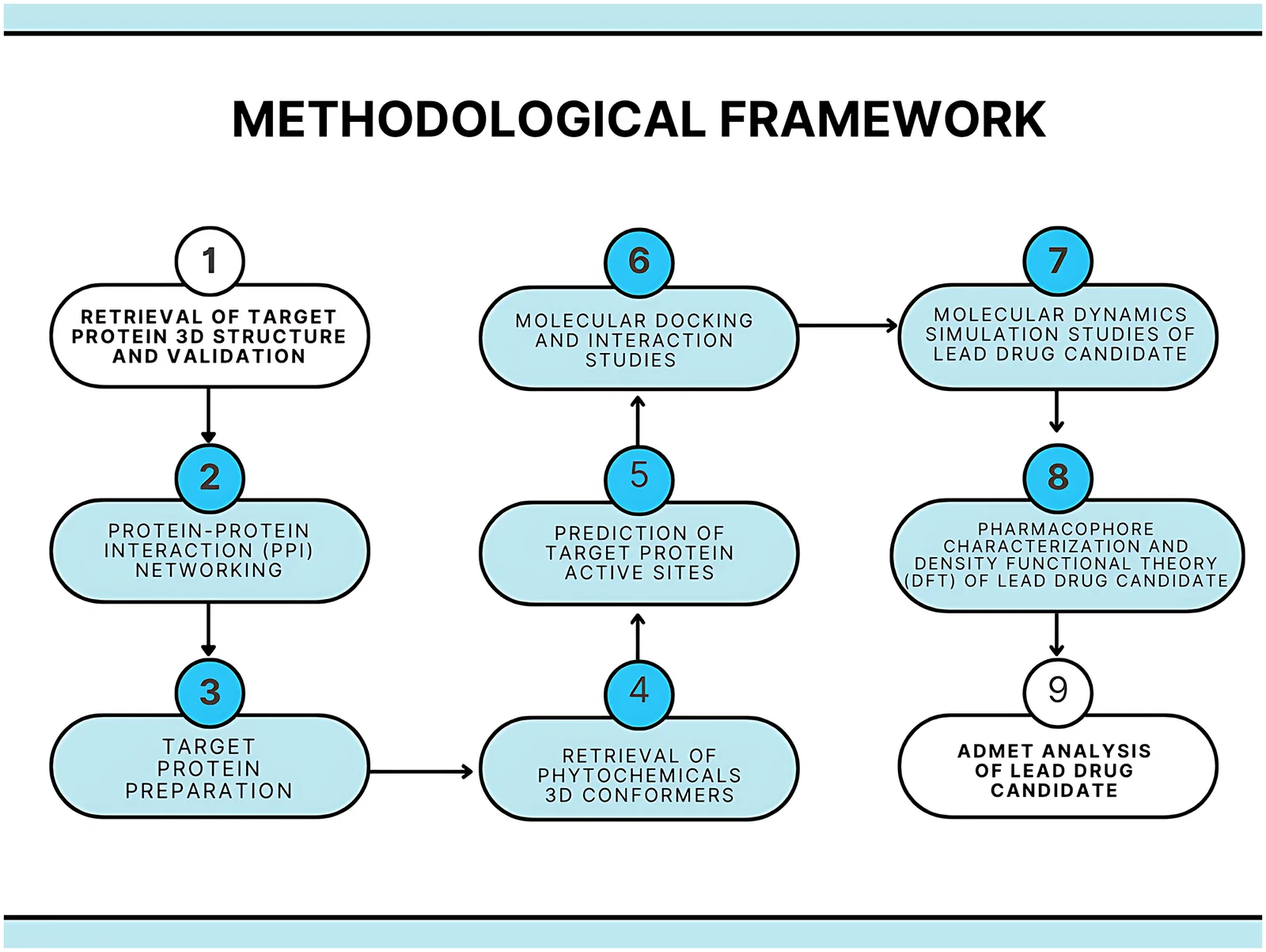
Graphical representation of the overall methodological workflow employed in the present study, illustrating the sequential computational pipeline.

### Selection and structural validation of the VEGFR1 kinase-domain structure

Experimental human VEGFR1 structures available in the Protein Data Bank (https://www.rcsb.org/) were examined to identify an appropriate template for kinase-pocket modeling. Representative alternative structures included PDB entries 1FLT and 1QTY, which contain VEGF-A bound to VEGFR1 extracellular domain 2; 1RV6, which contains PlGF bound to extracellular domain 2; and 5T89, which contains VEGF-A bound to VEGFR1 extracellular domains 1–6 [26–28]. Although these structures provide information about extracellular ligand recognition, they do not contain the intracellular ATP-binding pocket required for kinase-domain docking. PDB entry 3HNG was therefore selected because it experimentally resolves the intracellular VEGFR1 kinase domain in complex with the orthosteric inhibitor N-(4-chlorophenyl)-2-[(pyridin-4-ylmethyl)amino]benzamide, designated 8ST. The structure was determined by X-ray diffraction at a resolution of 2.70 Å [27,28]. Thus, selection of 3HNG prioritized the presence of an experimentally defined kinase pocket and a co-crystallized reference ligand; its 2.70 Å resolution was considered moderate rather than high.

The 3HNG biological assembly is monomeric and contains a single VEGFR1 protein chain, chain A. The deposited construct comprises two N-terminal expression-tag residues, Ser799 and Met800, followed by the human FLT1 kinase-domain sequence corresponding to UniProt residues 801–1158. No amino-acid substitutions are annotated within the aligned VEGFR1 sequence. Of the 360 deposited construct residues, 288 were modeled in the crystallographic structure. The unresolved residues comprised Ser799–Met800, Pro801–Asp802, Leu813, Asp926–Tyr990, and Gly1057–Asp1058. The entry additionally contains the co-crystallized inhibitor 8ST, one chloride ion, and 33 crystallographic water molecules. Because the construct lacks the extracellular, transmembrane, juxtamembrane, and distal C-terminal regions of full-length VEGFR1, it was used specifically to investigate ligand placement within the intracellular kinase pocket and not to model full-length receptor activation or extracellular ligand binding.

Structural-state annotation using the KLIFS kinase-structure database classified 3HNG chain A as adopting a DFG-out/αC-helix-in conformation [29]. Accordingly, 3HNG represents an inhibitor-stabilized, inactive-like kinase conformation rather than a ligand-activated state of the full-length receptor. This conformation was considered suitable for prioritizing compounds capable of occupying the inhibitor-defined ATP-binding pocket, while recognizing that it represents only one member of the conformational ensemble accessible to VEGFR1.

The stereochemical quality of the deposited 3HNG coordinates was initially evaluated using the SAVES v6.0 server (https://saves.mbi.ucla.edu/) [29]. PROCHECK was used to examine the distribution of backbone φ and ψ dihedral angles, while ERRAT was used to evaluate patterns of nonbonded atomic interactions [30,31]. As a complementary assessment, the full wwPDB X-ray structure-validation report for 3HNG was examined. This report applies MolProbity-based analyses (https://molprobity.biochem.duke.edu/) of backbone geometry, side-chain conformations, all-atom steric clashes, and bond-length, bond-angle, chirality, and planarity outliers [32]. Because PROCHECK and MolProbity apply different reference distributions and classification criteria, their Ramachandran results were recorded and reported separately. These validation metrics characterize the deposited crystallographic coordinates and were not interpreted as evidence of receptor activity, energetic stability, or biological efficacy.

### Preparation of VEGFR1 protein for further studies

Prior to performing molecular docking and molecular dynamics simulations, a three-dimensional structure of VEGFR1 (PDB ID: 3HNG) was refined to get a structurally accurate and biologically relevant model. The preprocessing involved using BIOVIA Discovery Studio Visualizer, which enabled the removal of extraneous components in the PDB structure, such as co-crystallized ligands, water molecules, and additional protein chains. The 3HNG biological assembly contains only one VEGFR1 protein chain, chain A; therefore, no additional protein chain was removed. The crystallographic coordinates of 8ST were retained separately as the experimental reference pose for redocking validation. The 8ST ligand, chloride ion, and crystallographic water molecules were removed from the receptor copy subsequently prepared for docking.

The deposited 3HNG structure contains 288 modeled residues from a 360-residue construct. The unresolved positions were Ser799–Met800, Pro801–Asp802, Leu813, Asp926–Tyr990, and Gly1057–Asp1058. PDBFixer, implemented through the SAMSON Connect workflow, was used to identify unresolved residues and missing heavy atoms and to prepare a continuous receptor model for subsequent calculations [33]. The extended unresolved region spanning Asp926–Tyr990 corresponds predominantly to the VEGFR1 kinase-insert segment and lies outside the KLIFS-defined ATP-binding pocket. Consequently, the reconstructed conformation of this segment was not used to assign protein–ligand contacts or to infer a specific biological mechanism.

The prepared receptor was subjected to energy minimization in Swiss-PDB Viewer using the GROMOS96 force field for 1,000 minimization steps [34]. A fixed number of minimization steps was applied; therefore, minimization was used to reduce local steric strain and was not interpreted as demonstrating convergence to a native or thermodynamically preferred receptor conformation.

### Retrieval of phytochemicals 3D conformers

Based on the existing literature, a heterogeneous group of phytochemicals with reported anti-angiogenic and anti-inflammatory activity was selected for molecular screening (S1 Table). These compounds were selected as a chemically diverse phytochemical library for evaluating predicted binding within the intracellular ATP-binding pocket of the VEGFR1 kinase domain. Their inclusion was intended for exploratory ligand prioritization and did not assume experimentally established VEGFR1 inhibition or modulation of extracellular PlGF–*sFlt-1* binding.

Three-dimensional (3D) conformers of the selected phytochemicals were downloaded from PubChem (https://pubchem.ncbi.nlm.nih.gov/), an openly accessible chemical database of the National Center for Biotechnology Information (NCBI) [35]. PubChem provides high-resolution molecular structure and physicochemical information that enables reproducible molecular-docking and simulation studies. For each substance the PubChem compound identifier (CID) was used to retrieve the respective 3D structure in the SDF format as well as SMILES notation which were then transformed to PDB to guarantee compatibility with molecular docking software.

The curated dataset consisted of naturally occurring flavonoids, alkaloids, phenolic acids, and glycosides from several botanical origins, such as *Rheum palmatum*, *Curcuma longa*, *and Camellia sinensis*. Compounds representing diverse bioactive scaffolds were deliberately included. This curated phytochemical library was used to provide the ligand pool for the subsequent docking, molecular-dynamics, and pharmacophore-characterization studies.

Three reference compounds were additionally included as experimental-pose and pharmacological comparators. The native crystallographic ligand, N-(4-chlorophenyl)-2-[(pyridin-4-ylmethyl)amino]benzamide (PDB chemical component ID: 8ST), was extracted directly from the original VEGFR1 crystal structure (PDB ID: 3HNG). Its crystallographic coordinates were preserved as the experimental reference pose, while a separate copy was prepared using the same ligand-preparation protocol applied during molecular docking and subsequently used for redocking validation. The 3HNG structure was experimentally resolved with 8ST in the VEGFR1 kinase domain, making this ligand the primary control for evaluating whether the docking protocol could reproduce the experimentally observed binding orientation. Lenvatinib and sorafenib were included as reference VEGFR-targeting multikinase inhibitors for comparative docking and interaction analysis. Three-dimensional structures of the parent compounds lenvatinib (PubChem CID: 9823820) and sorafenib (PubChem CID: 216239) were retrieved from PubChem in SDF format. The parent compounds, rather than lenvatinib mesylate or sorafenib tosylate salt forms, were used to ensure consistency in molecular preparation and docking comparisons.

### Prediction of target protein active sites

Binding-site prediction for VEGFR1 was performed using the PrankWeb machine learning pocket prediction server (https://prankweb.cz), which provides data-driven inference of ligandable cavities for grid definition and subsequent screening [36,37]. This step is important because an accurate pocket map can increase the relevance of docking poses and minimize the effect of false positives during virtual screening. The curated VEGFR1 model was uploaded in PDB format, the organism was defined as Homo sapiens and the default values of the PrankWeb parameters were used to generate ranked pockets. The highest-ranked pocket identified by PrankWeb was designated Pocket 1 and used for the subsequent docking-box placement and interaction analyses, consistent with a pre-defined strategy of the project to screen ligands within the most likely interacting area.

### Molecular docking and interaction studies of phytochemicals with VEGFR1

Molecular docking was used to compare the predicted binding poses and method-specific docking scores of the selected phytochemicals within the inhibitor-defined ATP-binding pocket of the intracellular VEGFR1 kinase domain. The docking results were used for computational candidate prioritization and were not interpreted as evidence of VEGFR1 inhibition or direct modulation of PlGF binding by soluble *sFlt-1*. Docking calculations were performed using AutoDock Vina Extended version (v.1.2.7) implemented within SAMSON version 2026 R1 (11.0.1) [38–40]. No user-defined random seed was specified; therefore, the default stochastic initialization implemented by the AutoDock Vina Extended module was used.

Prior to molecular docking, the prepared VEGFR1 receptor was treated as a rigid macromolecule, whereas ligand rotatable bonds were treated as flexible to permit conformational sampling within the binding pocket. The molecular connectivity and tautomeric form supplied in the retrieved PubChem structure were retained for each ligand. Alternative tautomeric states were not enumerated. Hydrogen atoms and partial charges were subsequently prepared to represent the selected ligand forms at a nominal pH of 7.4. During PDBQT preparation, polar hydrogen atoms were retained and nonpolar hydrogen atoms were merged with their corresponding carbon atoms. Kollman united-atom partial charges were assigned to the receptor, while Gasteiger–Marsili partial charges were assigned to each ligand. Receptor side-chain flexibility was not sampled during docking.

The docking grid was positioned over Pocket 1 identified by PrankWeb, which corresponded spatially to the ATP-binding pocket occupied by the co-crystallized ligand 8ST in the original 3HNG structure. The grid center was defined at x = 7.3 Å, y = 17.8 Å, and z = 30.8 Å. A cubic search space with dimensions of 20 × 20 × 20 Å along the x, y, and z axes, respectively, was applied using a grid spacing of 1 Å. AutoDock Vina conformational sampling was performed with an exhaustiveness value of 8. The receptor remained rigid throughout docking, whereas ligand rotatable bonds were treated as flexible.

Independent comparative docking was performed using Molecular Operating Environment (MOE), version 2024.06 [41]. The default MOE small-molecule docking settings were retained without modification. Ligand poses were generated using the Triangle Matcher placement algorithm and initially evaluated using the London dG scoring function. A maximum of 10 poses per ligand was retained following the placement stage. The retained poses were subjected to rigid-receptor refinement using the default AmberEHT force field, during which ligand geometry was refined while the receptor coordinates remained fixed. Refined poses were rescored using the GBVI/WSA dG scoring function, and the five highest-ranked final poses were retained for each ligand. The most favorably scored final pose was selected for comparative interaction analysis. Because AutoDock Vina and MOE employ different search algorithms and scoring functions, their absolute numerical scores were not treated as directly interchangeable binding-energy estimates. Instead, relative rankings, predicted pose placement, and protein–ligand interaction patterns were considered complementary outputs. Agreement between the AutoDock Vina and MOE rankings across the complete set of 21 phytochemicals and three reference ligands was evaluated using Spearman’s rank correlation coefficient, with more negative method-specific scores assigned higher ranks.

The docking protocol was further validated by redocking the native crystallographic ligand, N-(4-chlorophenyl)-2-[(pyridin-4-ylmethyl)amino]benzamide (PDB ligand ID: 8ST), into the ATP-binding pocket of VEGFR1. The ligand was extracted from the original PDB structure 3HNG and redocked using the same prepared receptor, grid box, and docking parameters applied to the phytochemical and reference-inhibitor library. The experimental and top-ranked redocked ligand poses, maintained within the same receptor coordinate frame, were submitted in MOL2 format to the Zhang Lab DockRMSD server (https://zhanggroup.org/DockRMSD/). A symmetry-corrected heavy-atom RMSD was calculated using graph-isomorphism-based atom mapping, and an RMSD of ≤2.0 Å relative to the crystallographic pose was considered indicative of near-native pose reproduction [42].

Following docking, the selected poses were examined using the Protein-Ligand Interaction Profiler (PLIP) (https://plip-tool.biotec.tu-dresden.de/), PyMOL, and BIOVIA Discovery Studio Visualizer to characterize predicted hydrogen bonds, hydrophobic contacts, and other non-covalent interactions [43–45]. These interaction profiles were considered together with docking rank and pose placement to support candidate prioritization for subsequent molecular dynamics and end-point energy analyses.

### Molecular dynamics simulation studies of lead candidate ligand with VEGFR1

All-atom molecular dynamics simulations were performed to characterize the time-dependent conformational behavior and interaction persistence of the computationally prioritized VEGFR1–Emodin-8-glucoside complex. A corresponding simulation of VEGFR1 bound to the co-crystallized inhibitor 8ST was performed under identical conditions as a crystallographic control. The starting Emodin-8-glucoside complex was obtained from its top-ranked docking pose within the ATP-binding pocket, whereas the experimentally resolved 8ST coordinates were retained from the original 3HNG crystal structure. The same prepared VEGFR1 receptor model was used in both systems to permit a consistent lead–control comparison. These simulations were used to examine conformational behavior after docking and were not interpreted as experimental confirmation of binding or inhibition.

Simulations were conducted using OpenMM version 8.4.0 with AMBER-compatible force fields. The VEGFR1 protein was parameterized using the AMBER ff19SB force field [31,46]. Ligand parameters were generated through an Antechamber-compatible workflow using the General AMBER Force Field 2 (GAFF2), and ligand partial atomic charges were assigned using the AM1-BCC method in AmberTools25 [47,48]. The formal charge and protonation state selected for each ligand were retained throughout the simulation. The protein–ligand complexes were solvated with explicit SPC water molecules in orthorhombic periodic boxes, maintaining a minimum distance of 10 Å between the solute and each box boundary [49,50]. Each system was electrically neutralized with Na+ or Cl− counterions, followed by the addition of NaCl to a final concentration of 0.15 M.

Each solvated system was subjected to 20,000 energy-minimization steps to reduce steric clashes and unfavorable local contacts. The minimized systems were equilibrated sequentially under the constant-volume and constant-temperature NVT ensemble at 300 K and the constant-pressure and constant-temperature NPT ensemble at 300 K and 1 atm. Temperature was controlled using a Langevin thermostat with a collision frequency of 1 ps − 1, while pressure was controlled using an isotropic Monte Carlo barostat with volume-change attempts every 25 integration steps. Long-range electrostatic interactions were treated using the particle-mesh Ewald method, and a 10 Å cutoff was applied to short-range nonbonded interactions. Bonds involving hydrogen atoms were constrained, permitting use of a 2 fs integration timestep [49,50].

After equilibration, one 500 ns production trajectory was generated for each VEGFR1–ligand complex under the NPT ensemble with periodic boundary conditions [50,51]. During production, the receptor backbone and side chains were allowed to move without positional restraints, and ligand internal degrees of freedom were treated flexibly; only bonds involving hydrogen were constrained. Coordinates were recorded every 10 ps, producing 50,000 saved frames per trajectory. The use of a fully flexible receptor during MD allowed the complexes to depart from their initially docked conformations, in contrast to the rigid-receptor approximation applied during molecular docking.

Trajectory analyses included protein-backbone, ligand, and ATP-binding-site root-mean-square deviation; residue-level root-mean-square fluctuation; radius of gyration; solvent-accessible surface area; protein–ligand hydrogen bonds; ligand-to-catalytic-site distance; and electrostatic and van der Waals interaction energies. Principal component analysis was used to examine dominant collective motions, while dynamic cross-correlation matrix analysis was used to evaluate correlated and anticorrelated residue motions. All trajectory analyses, including the hydrogen-bond calculations, were carried out with the CPPTRAJ module distributed in AmberTools25. Intermolecular receptor–ligand hydrogen bonds were identified geometrically using a donor heavy-atom to acceptor heavy-atom distance cutoff of 3.0 Å together with a donor–hydrogen–acceptor angle cutoff of 135°, and only direct hydrogen bonds formed between VEGFR1 and the bound ligand were counted, so that intramolecular protein–protein contacts and water-mediated bridges were excluded. The resulting per-frame counts were smoothed with a fifty-frame rolling average for presentation only. An earlier version of this analysis reported a markedly higher per-frame average because the count had not been restricted to direct intermolecular receptor–ligand donor–acceptor pairs; the values reported in this manuscript were recomputed under the criteria described above and supersede that earlier Fig [52]. Identical analytical procedures were applied to the Emodin-8-glucoside and 8ST trajectories. The calculated profiles describe conformational sampling within the individual trajectories and do not independently demonstrate receptor stabilization, binding affinity, or biological inhibition.

End-point binding-energy calculations were performed using the MMPBSA.py workflow implemented in AmberTools25 [47,53]. MM-GBSA and MM-PBSA calculations were applied separately to the VEGFR1–Emodin-8-glucoside and VEGFR1–8ST trajectories using the single-trajectory approach, in which the complex, receptor, and ligand energies were evaluated from the same coordinate frames. Ten snapshots were extracted at 50 ns intervals from 50 to 500 ns, including the final frame at 500 ns. The reported averages, standard deviations, and standard errors were calculated across these ten snapshots. Gas-phase molecular-mechanics, polar-solvation, and nonpolar-solvation contributions were calculated for each frame, and the total end-point binding-energy estimate was determined as the complex-minus-receptor-minus-ligand difference. Configurational entropy was not included. Because the ten snapshots were separated by 50 ns of simulation time, correlation between successive snapshots is expected to be limited; however, no block averaging or formal convergence assessment was performed, and the reported standard deviations and standard errors therefore describe the spread across this small snapshot set rather than a converged estimate of sampling uncertainty. Accordingly, the reported MM-GBSA and MM-PBSA values represent end-point energy estimates from a limited set of trajectory snapshots rather than experimentally measured binding free energies.

### Pharmacophore characterization and density functional theory (DFT) of lead candidate ligand

Pharmacophore modeling and electronic structure analysis were conducted to examine the molecular characteristics and electronic properties of the first compound, Emodin-8-glucoside, which may contribute to recognition by VEGFR1. The pharmacophore characterization was performed using the Pharmit platform (http://pharmit.csb.pitt.edu/), which provides an interactive ligand-based modeling environment and visualization of various pharmacophoric features, such as hydrogen-bond donors, hydrogen-bond acceptors, aromatic rings and hydrophobic centers [54,55]. This analysis allowed us to identify the spatial organization of key functional groups crucial for receptor recognition and binding, and thereby to provide a structural basis for our understanding of the molecular interactions in the binding cavity of VEGFR1.

Electronic-structure calculations for Emodin-8-glucoside were performed using Gaussian 09W (Gaussian, Inc., Wallingford, CT, USA) [56]. Geometry optimization was carried out using the B3LYP density functional with the 6-31G(d,p) basis set in the gas phase. Emodin-8-glucoside was treated as a neutral closed-shell system with a formal charge of 0 and a singlet spin multiplicity of 1. Following geometry optimization, the converged wavefunction was used to calculate the highest occupied molecular orbital (HOMO), lowest unoccupied molecular orbital (LUMO), and molecular electrostatic potential surface. The HOMO-LUMO energy gap was calculated as the difference between the LUMO and HOMO orbital energies. Orbital energies and the resulting energy gap were obtained in Hartree and converted to electronvolts using the conversion factor 1 Hartree = 27.2114 eV. These quantum-chemical descriptors were used to characterize the electronic properties of Emodin-8-glucoside and were not interpreted as direct measures of VEGFR1 binding affinity or inhibitory activity.

### ADMET analysis of lead candidate ligand

Selected physicochemical, pharmacokinetic, drug-likeness, and toxicity properties of Emodin-8-glucoside were evaluated computationally. SwissADME (http://www.swissadme.ch/) was used to predict molecular descriptors, lipophilicity, aqueous solubility, gastrointestinal absorption, blood-brain barrier permeability, P-glycoprotein substrate status, cytochrome P450 inhibition, Lipinski drug-likeness, and bioavailability score [57]. ProTox 3.0 (https://tox.charite.de/protox3/index.php?site=compoundsearchsimilarity) was used to predict acute oral toxicity class, estimated median lethal dose (LD50), organ-toxicity endpoints, general toxicity endpoints, nuclear-receptor signaling pathways, stress-response pathways, molecular initiating events, and metabolic-enzyme interactions [58]. Predictions and associated probabilities were recorded as generated by the respective computational models. Pregnancy-specific safety endpoints, including teratogenicity, embryotoxicity, reproductive toxicity, fetal toxicity, and placental transfer, were not evaluated by these analyses. Therefore, the ADMET predictions were used for preliminary candidate characterization rather than as evidence of maternal or fetal safety.

### Use of generative artificial intelligence

Generative artificial intelligence tools were used during manuscript preparation solely to assist with language editing, grammar correction, and improvement of readability. No generative artificial intelligence tool was used to design the computational workflow, to generate, process, or analyze any data, to prepare Figs or tables, or to interpret the work described here. All text drafted with such assistance was subsequently checked and verified by the authors, who take full responsibility for the content of the manuscript.

## Results

### Selection and structural validation of the VEGFR1 kinase-domain structure

PDB entry 3HNG provided an experimentally determined structure of the intracellular VEGFR1 kinase domain in complex with the inhibitor 8ST (Fig 2A). The structure was resolved by X-ray diffraction at 2.70 Å and contains one protein chain with 288 modeled residues from a 360-residue construct. No amino-acid mutations are annotated in the aligned VEGFR1 sequence. Comparison with representative alternative VEGFR1 structures showed that entries 1FLT, 1QTY, 1RV6, and 5T89 contain extracellular ligand-binding regions rather than the intracellular kinase domain. Therefore, 3HNG was selected because it provided both the kinase-domain coordinates and an experimentally observed ATP-pocket ligand. KLIFS classified the structure as DFG-out/αC-helix-in, indicating an inhibitor-bound, inactive-like kinase conformation.

**Fig 2:**
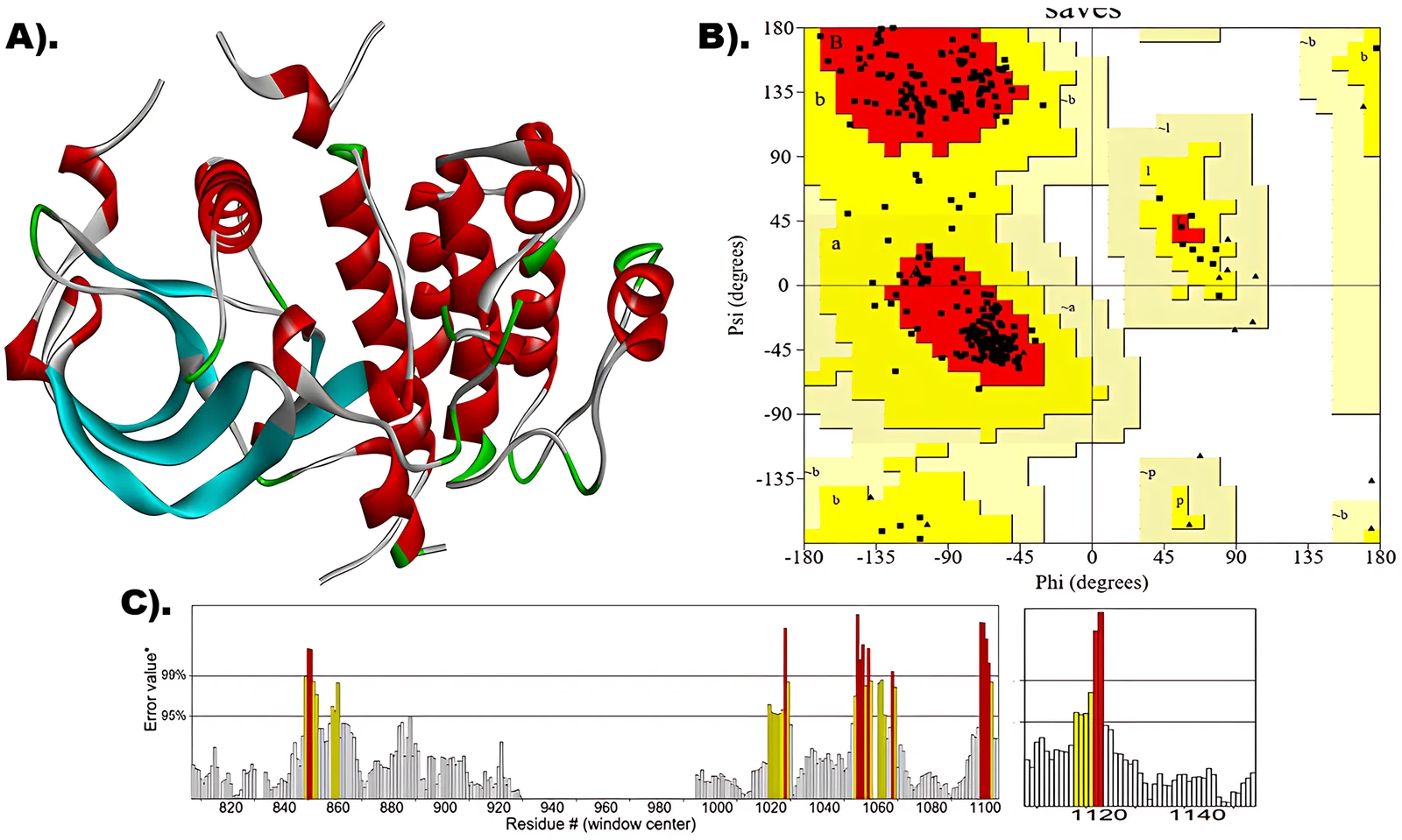
Structural representation and validation of the VEGFR1 kinase-domain structure (PDB ID: 3HNG). (A) Three-dimensional crystallographic structure of VEGFR1 visualized using BIOVIA Discovery Studio. (B) PROCHECK Ramachandran plot showing 85.5% of evaluated residues in the most favored regions and 14.5% in additionally allowed regions, with no residues in generously allowed or disallowed regions. (C) ERRAT quality profile showing an overall quality factor of 85.92.

PROCHECK analysis classified 85.5% of the evaluated residues within the most favored Ramachandran regions and 14.5% within additionally allowed regions, while no residues were assigned to generously allowed or disallowed regions (Fig 2B). The reported PROCHECK categories therefore accounted for 100% of the evaluated residues. ERRAT yielded an overall quality factor of 85.92, indicating generally acceptable nonbonded atomic interactions for the crystallographic model (Fig 2C).

Complementary MolProbity assessment provided a more detailed evaluation of backbone and side-chain geometry. Of the residues evaluated by MolProbity, 264 residues (94.29%) were classified within Ramachandran-favored regions, while one residue (0.36%) was identified as a Ramachandran outlier. The Ramachandran distribution Z-score was −2.71 ± 0.42. Side-chain analysis identified 200 residues (80.32%) in favored rotameric conformations and 18 residues (7.23%) as poor rotamers. No Cbeta deviations greater than 0.25 Å, bond-length outliers, bond-angle outliers, or chiral-volume outliers were detected. One twisted peptide (0.35%) and seven CaBLAM outliers (2.6%) were identified. Taken together, these validation results indicate that the 3HNG crystallographic structure possesses generally acceptable backbone and covalent geometry while retaining localized conformational limitations that should be considered when interpreting structure-based predictions.

### Prediction of target protein active sites

Potential binding cavities in the VEGFR1 structure were identified using the PrankWeb server, which is an artificial intelligence-based server for the prediction of ligand binding pockets that relies on the analysis of spatial, physicochemical and evolutionary properties of protein surfaces. Precise mapping of such pockets can be important to defining docking grids, as well as to ensuring that ligands interact with biologically relevant sites and not random locations at the surface.

Following the submission of the validated VEGFR1 structure, PrankWeb identified five different binding pockets based on their probability and score of observations of conservation. Pocket 1 had the highest probability score of 0.97 and consisted of 43 residues, with an average conservation value of 2.07 (Table 1 and Fig 3). These metrics indicate substantial structural and evolutionary conservation and identify Pocket 1 as the most probable functional binding site of VEGFR1. The predicted center of Pocket 1 was located at approximately x = 7.39 Å, y = 17.90 Å, and z = 30.88 Å, corresponding spatially to the ATP-binding pocket occupied by the co-crystallized ligand 8ST.

**Table 1:** Five predicted binding pockets of VEGFR1 generated by PrankWeb, showing score, probability, number of residues, average conservation score, and spatial dimensions (X, Y, Z).

No.	Score	Probability	No. of Residues	Avg Conservation	Pocket-center coordinates (Å)		
					X	Y	Z
1	45.24	0.970	43	2.069	7.3883	17.896	30.8766
2	3.18	0.111	10	1.268	6.7067	7.8616	19.1595
3	1.10	0.009	4	0.282	−6.1999	9.1232	49.3812
4	0.88	0.004	4	1.255	9.344	16.6504	7.5772
5	0.87	0.004	7	0.427	23.0648	16.5072	12.1037

**Fig 3:**
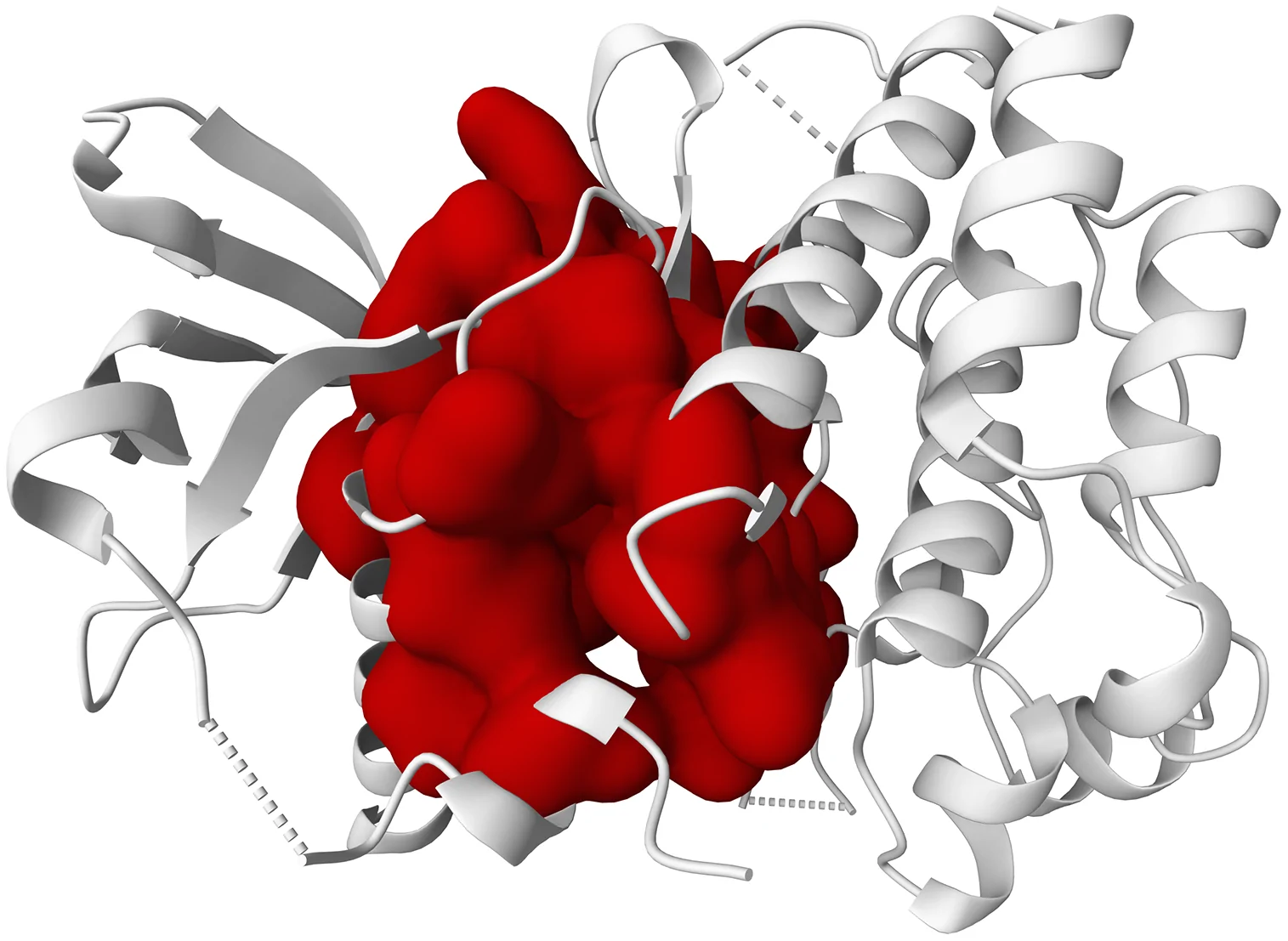
Predicted binding pocket 1 of VEGFR1 generated by PrankWeb. The top-ranked pocket (Pocket 1, shown in red) was identified as the most probable ligand-binding site based on probability and conservation scores, located within the kinase domain of VEGFR1.

Fig 3 shows that Pocket 1 (highlighted in red) lies within the kinase domain of VEGFR1, in agreement with the known ligand-recognition region of the receptor. The residues defining this cavity include important polar and hydrophobic amino acids frequently linked to substrate and inhibitor binding for tyrosine kinase receptors. This structural delineation provides a strong framework for defining the docking grid box and for the subsequent molecular docking and molecular dynamics simulation studies, confining the computational exploration to the most biologically plausible interaction site of VEGFR1.

### Molecular docking and interaction studies of phytochemicals with VEGFR1

Molecular docking was used to compare the predicted binding poses and method-specific docking scores of 21 phytochemicals and three reference ligands within the ATP-binding pocket of VEGFR1. The reference compounds included the co-crystallized ligand 8ST, lenvatinib, and sorafenib. The complete AutoDock Vina and MOE docking results are presented in Table 2. The AutoDock Vina scores of the 21 phytochemicals are additionally plotted in Fig 4.

**Table 2:** Comparative docking outputs of 21 phytochemicals and three reference ligands against VEGFR1 obtained using AutoDock Vina Extended and MOE.

No.	Compound	AutoDock Vina	MOE	
		Predicted Binding Affinity (kcal/mol)	RMSD_refine (Å)	Predicted Binding Affinity (kcal/mol)
1.	Apigenin	−8.324	1.0796	−5.8073
2.	Apigenin 7-O-glucoside	−8.898	1.4053	−8.2755
3.	Baicalein	−7.949	1.9744	−5.5523
4.	Berberine	−8.416	1.4013	−6.5447
5.	Beta Sitosterol	−8.145	0.8953	−4.0819
6.	Caffeic Acid Phenethyl Ester	−8.145	1.8196	−6.4509
7.	Curcumin	−8.129	1.5690	−6.4201
8.	Daidzein	−8.528	1.8785	−5.1934
9.	Ellagic acid	−8.738	1.3357	−5.9592
10.	Emodin-8-glucoside	−9.311	1.0843	−8.3229
11.	Epigallocatechin	−8.099	0.7848	−4.4160
12.	Fisetin	−8.012	1.9097	−4.7632
13.	Genistein	−8.270	0.8848	−5.5556
14.	Gallic Acid	−4.737	1.1457	−4.8930
15.	Kaempferol	−7.556	0.5597	−6.0932
16.	Luteolin	−7.722	0.7904	−6.0971
17.	Quercetin	−8.005	2.0451	−5.2632
18.	Resveratrol	−7.525	1.4612	−5.1844
19.	Rosmarinic acid	−8.113	1.2770	−6.4361
20.	Rutin	−8.244	2.0737	−6.2267
21.	Wogonin	−8.139	2.8525	−5.9737
22.	Co-crystallized ligand 8ST^a^	−8.514	0.9157	−7.6025
23.	Lenvatinib	−8.290	1.8558	−8.2877
24.	Sorafenib	−8.473	3.0932	−7.5157

More negative values indicate more favorable method-specific docking scores within the respective scoring functions. AutoDock Vina and MOE use different search algorithms and scoring functions; therefore, their absolute numerical scores were not interpreted as directly equivalent binding-energy estimates. The MOE RMSD_refine values describe the positional change occurring during the MOE refinement stage and do not represent RMSD relative to an experimentally determined ligand pose. AutoDock Vina RMSD values of 0.00 Å for individual top-ranked poses were not included because these values result from comparison of each top-ranked pose with itself. Validation against an experimental pose was performed separately by redocking the co-crystallized ligand 8ST.

^a^8ST: N-(4-chlorophenyl)-2-[(pyridin-4-ylmethyl)amino]benzamide, the native co-crystallized ligand extracted from PDB structure 3HNG.

**Fig 4:**
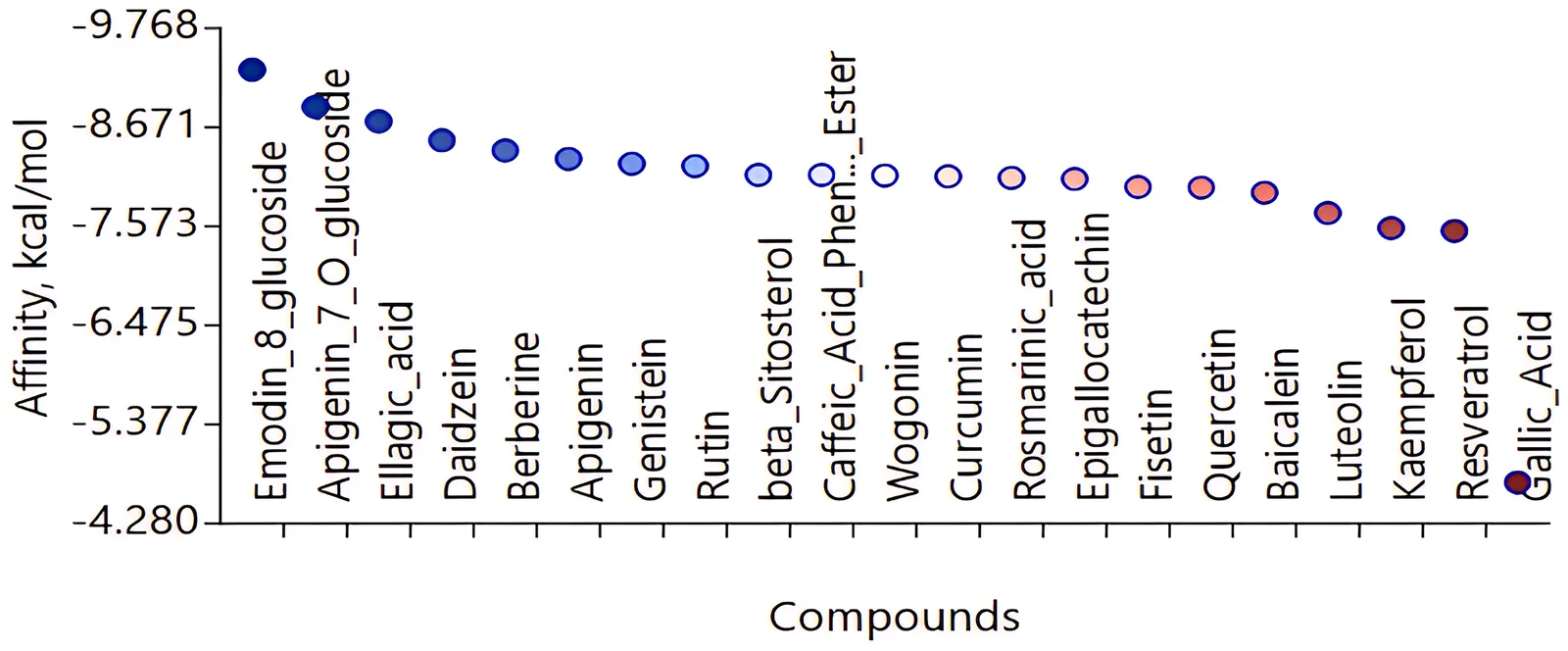
Comparative docking scores of twenty-one phytochemicals screened against VEGFR1 using AutoDock Vina Extended. Emodin-8-glucoside produced the most favorable AutoDock Vina score among the evaluated compounds (−9.311 kcal/mol); however, docking-score differences were used for computational ranking and not interpreted as experimentally resolved differences in binding affinity.

AutoDock Vina assigned Emodin-8-glucoside the most favorable score among the evaluated compounds at −9.311 kcal/mol, followed by apigenin 7-O-glucoside at −8.898 kcal/mol and ellagic acid at −8.738 kcal/mol. The corresponding Vina scores for the reference compounds 8ST, sorafenib, and lenvatinib were −8.514, −8.473, and −8.290 kcal/mol, respectively. Accordingly, Emodin-8-glucoside ranked first in the Vina screening, whereas 8ST, sorafenib, and lenvatinib ranked fifth, sixth, and ninth, respectively. Because docking-score differences of this magnitude do not directly represent experimentally resolvable differences in binding affinity, the ranking was used for computational prioritization rather than as evidence that Emodin-8-glucoside binds more strongly than the reference inhibitors.

The independent MOE analysis also placed Emodin-8-glucoside among the highest-ranked compounds, with a GBVI/WSA dG score of −8.3229 kcal/mol. Lenvatinib and apigenin 7-O-glucoside produced closely comparable MOE scores of −8.2877 and −8.2755 kcal/mol, respectively, corresponding to differences of only 0.0352 and 0.0474 kcal/mol relative to Emodin-8-glucoside. The co-crystallized ligand 8ST and sorafenib produced MOE scores of −7.6025 and −7.5157 kcal/mol, respectively. Thus, the MOE results supported the high ranking of Emodin-8-glucoside but did not indicate a substantial numerical separation from the next highest-ranked compounds.

Comparison of the complete AutoDock Vina and MOE rankings showed moderate positive concordance across the 24 evaluated compounds (Spearman’s rho = 0.553, p = 0.005). Although Emodin-8-glucoside ranked first in both workflows, several other compounds exhibited method-dependent rank changes, reflecting differences in the search and scoring procedures used by AutoDock Vina and MOE. Consequently, candidate prioritization was based on docking rank, predicted pose placement, reference-ligand comparison, and protein-ligand interaction patterns rather than direct comparison of the absolute numerical scores generated by the two docking programs.

Structural examination of the selected Emodin-8-glucoside pose showed occupancy of the VEGFR1 ATP-binding pocket (Fig 5). Hydrophobic contacts were predicted with Lys861, Leu882, Val891, and Val909, while hydrogen-bond interactions involved Asp807, Lys861, Glu878, Ile1019, Arg1021, and Asp1040 (Tables 3 and 4). These predicted contacts supported retention of Emodin-8-glucoside for subsequent molecular dynamics and end-point energy analyses but were not interpreted as experimental evidence of VEGFR1 inhibition, potency, or selectivity.

**Table 3:** Summary of hydrophobic interactions between VEGFR1 and Emodin-8-glucoside residues identified by PLIP and BIOVIA Discovery Studio.

Index	Residue	AA	Distance (Å)	Ligand Atom	Protein Atom
1	861A	LYS	3.92	4695	906
2	861A	LYS	3.87	4691	908
3	882A	LEU	3.74	4689	1248
4	891A	VAL	3.83	4684	1390
5	909A	VAL	3.71	4688	1655

**Table 4:** List of hydrogen bonds formed between VEGFR1 and Emodin-8-glucoside, including donor–acceptor distances, angles, and participating residues.

Index	Residue	AA	Distance H-A	Distance D-A	Donor Angle	Protein donor?	Side Chain	Donor Atom	Acceptor Atom
1	807A	ASP	3.17	3.79	120.45	Yes	No	67 [Nam]	4697 [O3]
2	807A	ASP	3.65	4.01	104.44	No	Yes	4699 [O3]	74 [O-]
3	861A	LYS	2.40	2.86	106.32	Yes	Yes	910 [N3+]	4703 [O3]
4	878A	GLU	3.19	3.59	106.08	No	Yes	4700 [O3]	1175 [O-]
5	1019A	ILE	3.37	4.04	124.65	Yes	No	2395 [Nam]	4697 [O3]
6	1021A	ARG	2.41	2.93	111.95	Yes	Yes	2440 [Ng+]	4698 [O3]
7	1021A	ARG	2.23	2.93	125.90	No	Yes	4698 [O3]	2440 [Ng+]
8	1040A	ASP	2.97	3.72	131.21	Yes	No	2739 [Nam]	4704 [O3]

**Fig 5:**
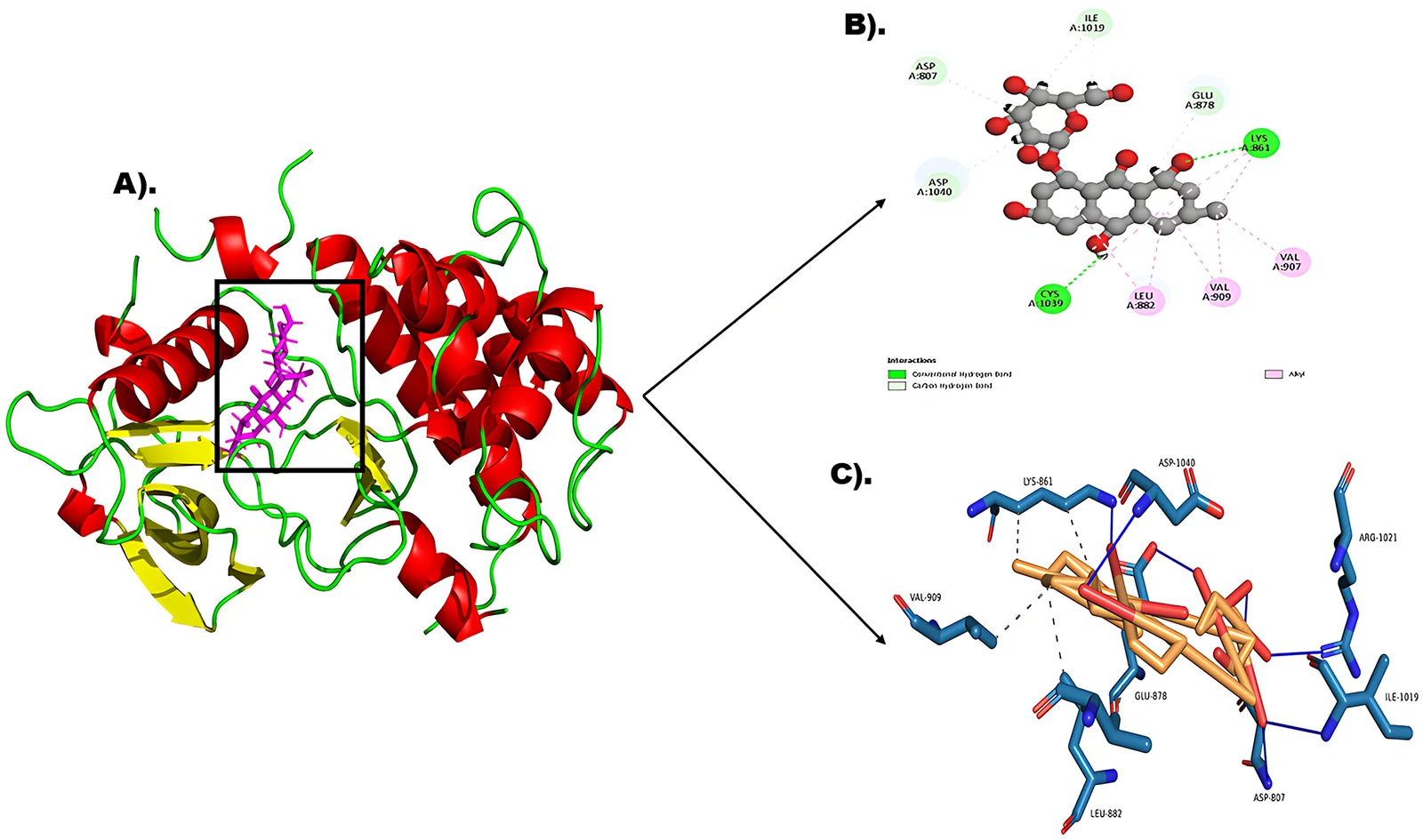
3D and 2D visualization of the VEGFR1–Emodin-8-glucoside interaction complex. (A). Docked pose of Emodin-8-glucoside within Pocket 1 of VEGFR1. (B). 2D interaction diagram depicting hydrogen bonds and hydrophobic contacts between the ligand and active-site residues. (C). Surface representation showing ligand occupancy within the receptor binding cavity.

### Redocking validation of the molecular docking protocol

The native crystallographic ligand 8ST was redocked into the ATP-binding pocket of VEGFR1 to evaluate whether the applied docking protocol could reproduce its experimentally observed binding orientation. Comparison of the crystallographic and top-ranked redocked poses using DockRMSD produced a symmetry-corrected heavy-atom RMSD of 1.465 Å. The experimental PDB 3HNG complex and crystallographic ligand pose are presented in Fig 6A, the top-ranked redocked complex and ligand pose are shown in Fig 6B, and the alignment of the experimental and redocked ligand poses is presented in Fig 6C. The symmetry-corrected heavy-atom RMSD of 1.465 Å was below the predefined 2.0 Å near-native pose criterion, indicating that the applied docking protocol reproduced the crystallographic orientation of 8ST with reasonable positional agreement. This result specifically supports pose-reproduction performance and does not independently demonstrate binding affinity, biological inhibition, or ligand stability.

**Fig 6:**
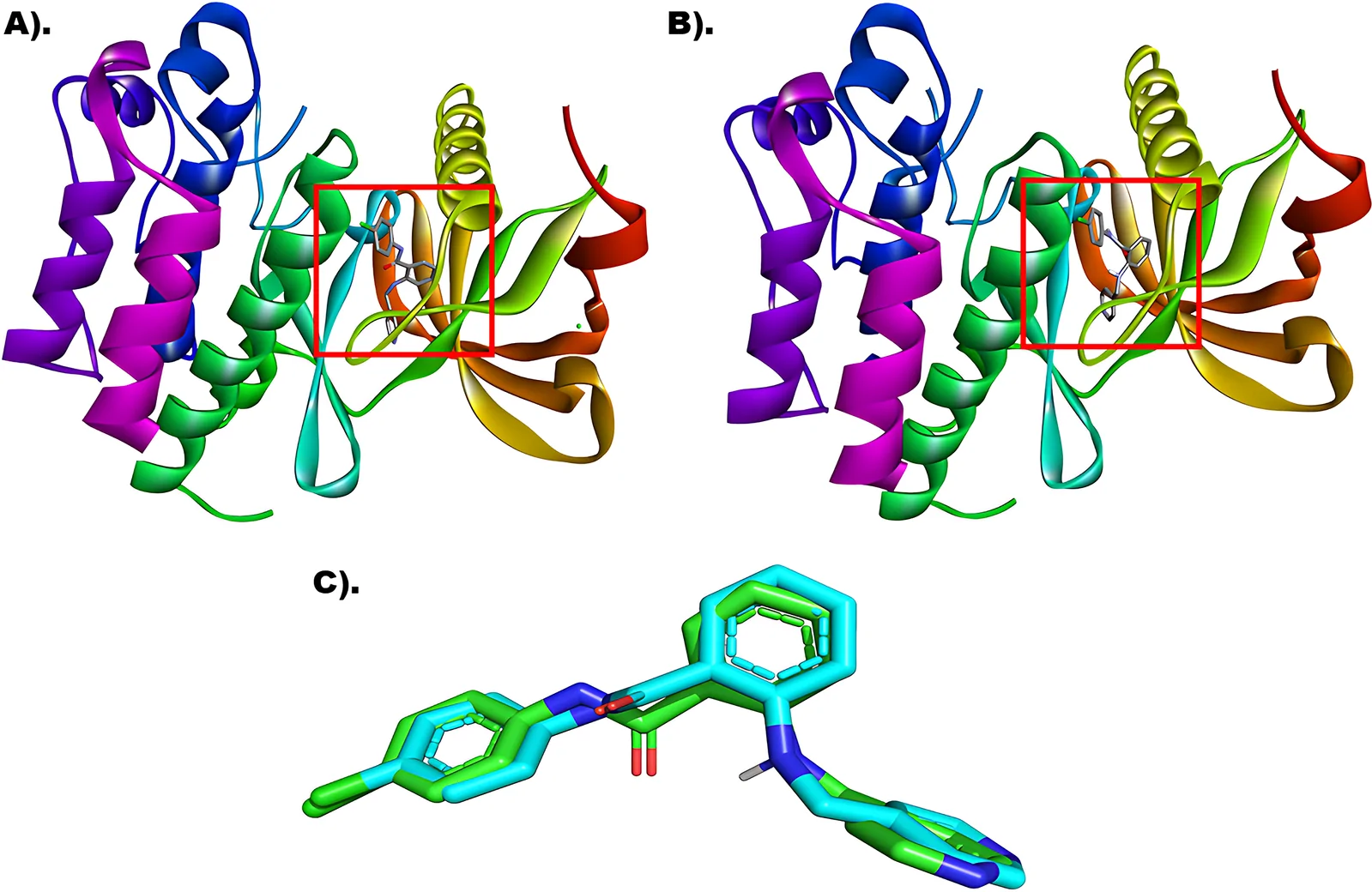
Redocking validation of the co-crystallized ligand N-(4-chlorophenyl)-2-[(pyridin-4-ylmethyl)amino]benzamide (PDB ligand ID: 8ST) against VEGFR1. (A). Experimental PDB 3HNG structure showing the crystallographic pose of 8ST within the VEGFR1 ATP-binding pocket. (B). Top-ranked pose obtained after redocking 8ST using the applied AutoDock Vina Extended protocol. (C). Alignment of the experimental and redocked ligand poses, shown as cyan and green sticks, respectively. DockRMSD calculated a symmetry-corrected heavy-atom RMSD of 1.465 Å between the two poses.

### Molecular dynamics simulations of VEGFR1–ligand complexes

#### Molecular dynamics simulation of the VEGFR1–Emodin-8-glucoside complex.

The dynamic behavior and conformational flexibility of the VEGFR1-Emodin-8-glucoside complex were examined over the 500 ns molecular dynamics trajectory.

**RMSD Analysis:** The root-mean-square deviation (RMSD) profile provides a quantitative measure for backbone and ligand stability over the 500 ns trajectory (Fig 7A). Backbone RMSD increased during the equilibration phase (0–20 ns) but stabilized during the simulation, resulting in an average deviation of about 0.18 nm thereafter. Ligand RMSD remained slightly below 0.15 nm, while the active-site residues deviated by approximately 0.20 nm, indicating bounded deviation within the binding region. After the initial conformational adjustment, the RMSD profiles remained bounded over the remainder of the trajectory, indicating persistence of the modeled VEGFR1-Emodin-8-glucoside complex without progressive structural divergence.

**Fig 7:**
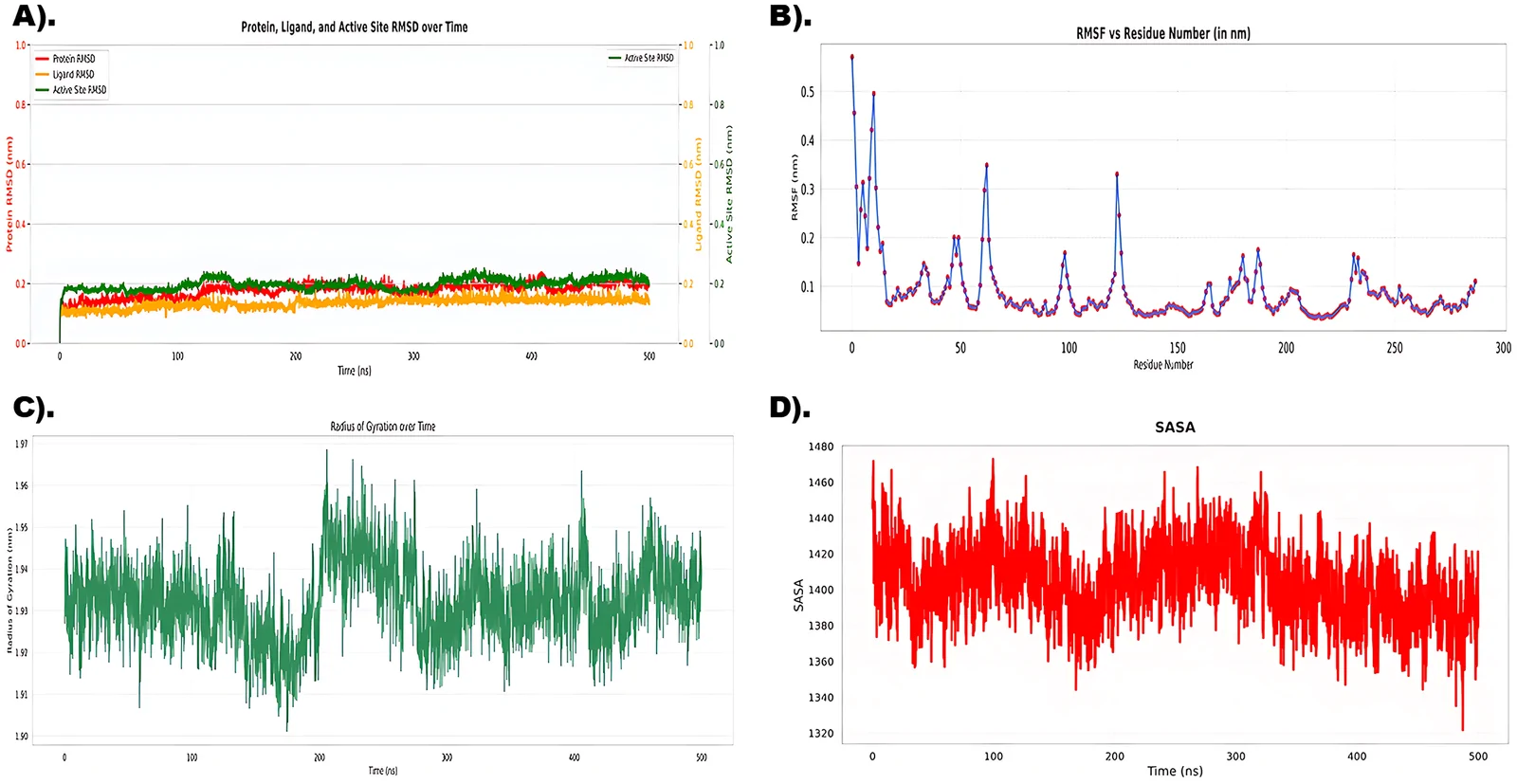
Structural descriptors of the VEGFR1-Emodin-8-glucoside complex during the 500 ns molecular dynamics simulation. (A) RMSD profiles of the protein backbone, ligand, and binding-site residues. (B) Residue-wise RMSF profile. (C) Radius-of-gyration profile describing global protein compactness. (D) Solvent-accessible surface area profile describing time-dependent protein surface exposure.

**RMSF Analysis:** The root mean square fluctuation (RMSF) plot (Fig 7B) describes residue-wise flexibility over the course of the whole simulation. The majority of residues showed fluctuations below 0.15 nm, which is indicative of the stability of secondary structural components. Only a few terminal and loop residues showed moderate fluctuations in the 0.3–0.5 nm range, characteristic of solvent-exposed flexible loops. The comparatively low fluctuation of the residues forming the binding pocket is consistent with retention of the predicted Emodin-8-glucoside interactions during the trajectory.

**Radius of Gyration (rGyr):** The radius of gyration (rGyr) analysis (Fig 7C) gave an assessment of the protein’s compactness over the course of the simulation. rGyr values fluctuated within a small range of 1.91–1.96 nm (average 1.93 nm), indicating that VEGFR1 maintained a tightly folded conformation and did not experience significant unfolding or expansion events. The absence of sustained directional drift in the radius-of-gyration profile indicates that VEGFR1 retained a broadly consistent global compactness during the Emodin-8-glucoside-bound trajectory.

**Solvent Accessible Surface Area (SASA):** The SASA profile (Fig 7D) fluctuated between approximately 1320 and 1470 Å^2^, with most values between about 1360 and 1440 Å^2^. Values were highest during the first few nanoseconds, settled at roughly 1390–1420 Å^2^ between approximately 50 and 320 ns, and were slightly lower, at roughly 1380–1400 Å^2^, from approximately 330 ns to the end of the trajectory. This modest late decrease reflected a small reduction in solvent-exposed surface area, although the shift was small relative to the frame-to-frame fluctuation and no continuous drift was evident. Overall, the bounded SASA profile indicates that protein surface exposure varied within a limited range during the simulated trajectory.

**Hydrogen Bond Dynamics:** The intermolecular hydrogen-bond analysis of the VEGFR1-Emodin-8-glucoside complex demonstrated persistent but dynamically varying polar interactions throughout the 500 ns molecular dynamics trajectory (Fig 8A). The hydrogen-bond profile showed recurrent formation and disruption of intermolecular contacts, with the 50-frame rolling mean remaining within a comparatively narrow range throughout most of the simulation. The raw hydrogen-bond profile fluctuated predominantly within approximately 0.5–2.5 hydrogen bonds, with occasional transient peaks reaching approximately 3.5. The 50-frame rolling mean remained generally between approximately 0.7 and 2.2 hydrogen bonds over the course of the simulation.

**Fig 8:**
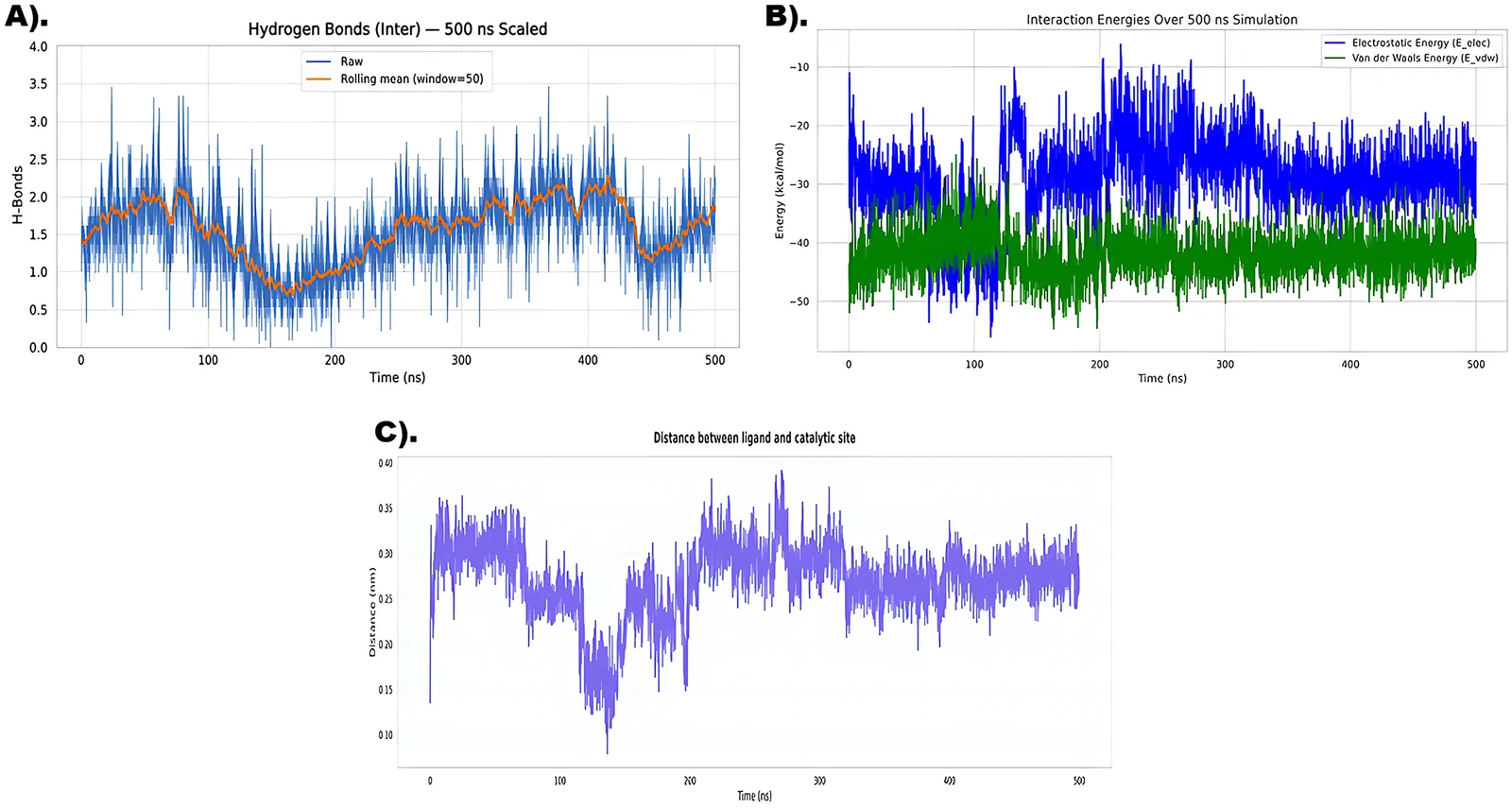
Energetic and hydrogen bonding analysis during 500 ns MD simulation. (A). Intermolecular hydrogen-bond count and 50-frame rolling-average profile obtained using the hydrogen-bond distance and angle criteria stated in the Methods. (B). van der Waals and electrostatic interaction-energy profiles showing time-dependent nonbonded interaction components. (C). Time-dependent distance between Emodin-8-glucoside and the catalytic-site region.

During the initial approximately 0−100 ns period, the rolling-average hydrogen-bond profile increased from approximately 1.4 to around 1.8–2.1, indicating recurrent intermolecular hydrogen-bond formation during the early trajectory. A temporary decrease was observed between approximately 120 and 190 ns, where the rolling mean declined to approximately 0.7–1.1. Hydrogen-bond interactions subsequently increased again after approximately 200 ns, with the rolling mean generally remaining between approximately 1.5 and 2.2 during much of the 250−430 ns interval. A second transient reduction occurred around 440−460 ns, followed by recovery toward approximately 1.8–1.9 near the end of the 500 ns trajectory.

Overall, the corrected profile indicates that VEGFR1 and Emodin-8-glucoside maintained recurrent intermolecular hydrogen-bond interactions throughout the simulation, although both the number and persistence of these interactions varied considerably with time. These fluctuations are consistent with continuous rearrangement of the ligand-protein hydrogen-bond network during conformational sampling. The hydrogen-bond count was therefore interpreted as a descriptor of time-dependent polar contacts and not as an independent measure of binding affinity, inhibitory potency, or complex stability.

**Interaction Energy Profile:** The van der Waals and electrostatic interaction energies were calculated to assess the energetic contributions that determined the receptor-ligand binding stability. As shown in Fig 8B the van der Waals component (EvdW) remained close to an average of approximately −45 kcal/mol, while the electrostatic component (Eelec) oscillated around −30 kcal/mol over the whole trajectory. Both interaction-energy components remained predominantly negative during the trajectory, indicating favorable direct nonbonded interactions under the simulated conditions. The persistently negative values indicate sustained intermolecular attraction, with dispersion and electrostatic contributions acting together within the modeled complex. These profiles characterize persistent van der Waals and electrostatic interactions between VEGFR1 and Emodin-8-glucoside but should not be interpreted as binding free energies or independent evidence of thermodynamic stability.

**Ligand-Catalytic Site Distance:** The distance between the geometric center of the ligand and the catalytic site residues of VEGFR1 was monitored to determine the spatial consistency and the binding stability during the simulation. As shown in Fig 8C, the distance oscillated between approximately 0.20 and 0.35 nm and settled near 0.28 nm after the first 80 ns of simulation. The bounded distance profile indicates that Emodin-8-glucoside remained spatially associated with the catalytic-site region throughout the analyzed trajectory. This positional persistence supports retention of the modeled ligand within the binding-site region but does not independently establish binding affinity or inhibitory potency.

**Principal Component Analysis (PCA):** A 500-ns molecular dynamics trajectory for the VEGFR1-Emodin-8-glucoside complex was subjected to Principal Component Analysis (PCA) to explore the large-scale conformational dynamics of the complex. The first two principal components (PC1 and PC2) were projected as a function of simulation time, which allowed visualizing the global motion of the protein along the trajectory (Fig 9A).

**Fig 9:**
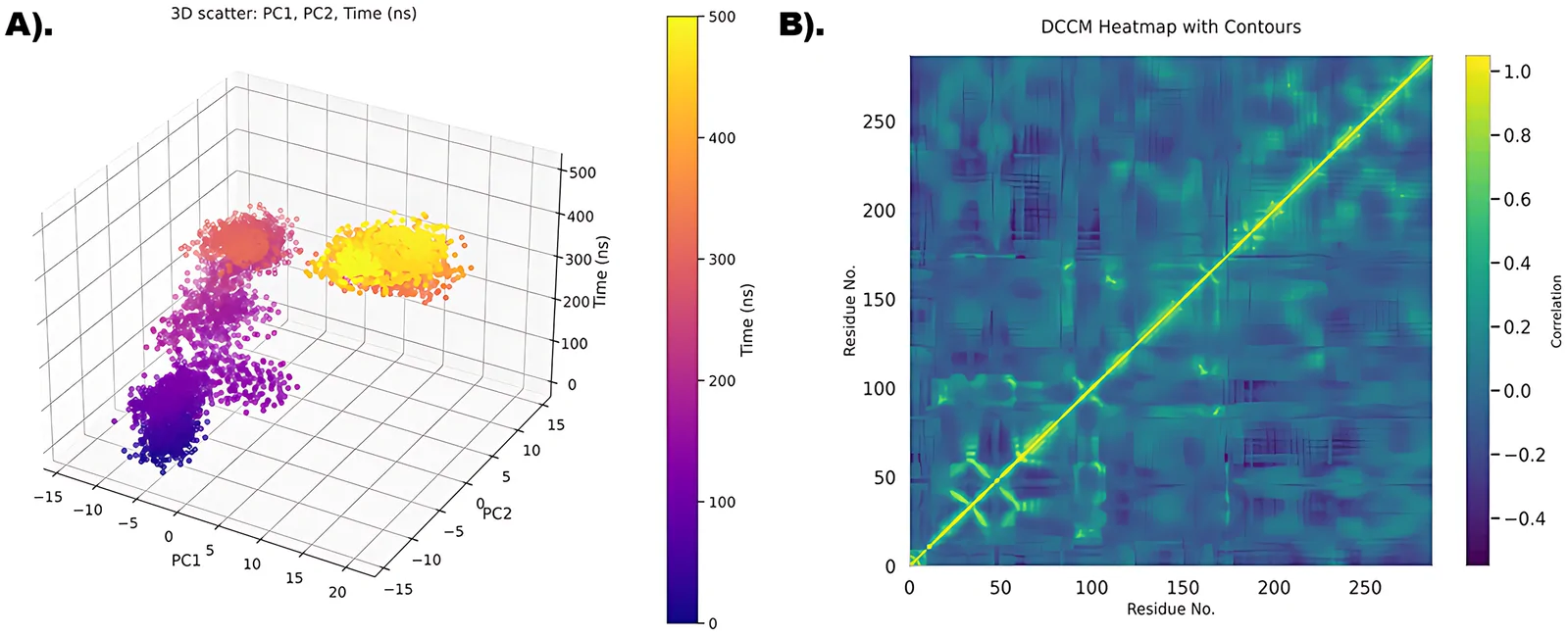
Collective-motion analyses of the VEGFR1-Emodin-8-glucoside trajectory. (A) Principal component analysis showing time-dependent distribution of trajectory frames across the conformational space defined by PC1 and PC2. (B) Dynamic cross-correlation matrix illustrating correlated and anticorrelated residue motions within VEGFR1.

The trajectory projection revealed three subpopulations of conformational trajectories lying along the PC1-PC2 plane. Crossovers between these clusters could be directly observed until around 200 ns when the system settled into a single, compact cluster at 250−500 ns. This temporal clustering behavior suggests that the VEGFR1-Emodin-8-glucoside complex reached a dynamic equilibrium after approximately 250 ns, with limited large-scale fluctuation thereafter. The decreased conformational spreading in the latter half of the simulation is consistent with the complex sampling a more restricted conformational region. The PCA profile indicates time-dependent conformational sampling followed by increased occupation of a comparatively restricted region of conformational space during the later trajectory. In the absence of an apo-receptor simulation and independent replicate trajectories, this pattern was not interpreted as evidence that ligand binding reduced VEGFR1 flexibility.

**Dynamic Cross-Correlation Matrix (DCCM) Analysis:** A Dynamic Cross-Correlation Matrix (DCCM), which describes the internal motion correlations among the residues over the course of the simulation, was also generated (Fig 9B). The correlated component heat map (DCCM) shows the extent of correlated (positive values) and anti-correlated (negative values) motions between pairs of residues, thus providing an indication of the inter-residue communication networks and dynamic coupling in the protein fold.

The DCCM plot showed a strong diagonal correlation (r ~ 1.0), which points to intra-domain coherence, whereas moderate positive correlations (r = 0.4–0.7) were detected among residues belonging to the kinase domain. A small number of anticorrelated patches (r = −0.3) were also found, mainly consisting of flexible loop regions and remote domain interfaces. The observed correlation pattern indicates coordinated and anticorrelated residue motions within the simulated VEGFR1-Emodin-8-glucoside complex. These correlations describe the dynamic coupling sampled during the trajectory but do not establish a ligand-induced stabilizing effect.

**MM-GBSA and MM-PBSA end-point binding-energy analysis of the Emodin-8-glucoside complex:** End-point energy decomposition of the VEGFR1-Emodin-8-glucoside complex produced substantially different total estimates under the generalized Born and Poisson-Boltzmann implicit-solvent models. The MM-GBSA calculation yielded a mean total binding-energy estimate of −43.11 ± 4.92 kcal/mol (mean ± standard deviation; Table 5). Favorable gas-phase contributions arose from van der Waals (−44.68 kcal/mol) and electrostatic (−51.98 kcal/mol) interactions, producing a combined gas-phase contribution of −96.66 kcal/mol. These favorable interactions were partially opposed by the generalized Born solvation contribution, resulting in a net solvation term of +53.54 kcal/mol.

**Table 5:** Molecular Mechanics Generalized Born Surface Area (MM-GBSA) energy decomposition of the VEGFR1–Emodin-8-glucoside complex from 500 ns trajectory.

Energy Component	Average	Std. Dev.	Std. Err. of Mean
VDWAALS	−44.6820	4.1096	1.2996
EEL	−51.9750	11.1595	3.5289
EGB	60.1133	7.4140	2.3445
ESURF	−6.5700	0.5775	0.1826
DELTA G gas	−96.6570	8.1953	2.5916
DELTA G solv	53.5434	7.2743	2.3003
DELTA TOTAL	−43.1137	4.9162	1.5546

In contrast, the MM-PBSA calculation yielded a mean total binding-energy estimate of −1.63 ± 9.70 kcal/mol (Table 6). Although the gas-phase contribution remained −96.66 kcal/mol, the Poisson-Boltzmann treatment produced a substantially larger net solvation penalty of +95.03 kcal/mol. Consequently, the favorable gas-phase interactions were almost completely offset by solvation effects. The comparatively large standard deviation relative to the MM-PBSA mean indicates substantial variability among the analyzed snapshots, with the sampled values extending across both negative and positive total-energy estimates. Therefore, the MM-PBSA result was not interpreted as independent evidence of favorable binding.

**Table 6:** MM-PBSA end-point energy decomposition of the VEGFR1-Emodin-8-glucoside complex calculated from the 500 ns molecular dynamics trajectory. Values represent complex – receptor – ligand energy differences and are reported in kcal/mol.

Energy Component	Average	Std. Dev.	Std. Err. of Mean
VDWAALS	−44.6820	4.1096	1.2996
EEL	−51.9750	11.1595	3.5289
EPB	67.1184	10.5383	3.3325
ENPOLAR	−35.5619	2.1172	0.6695
EDISPER	63.4724	2.8158	0.8904
DELTA G gas	−96.6570	8.1953	2.5916
DELTA G solv	95.0289	10.5512	3.3366
DELTA TOTAL	−1.6281	9.7012	3.0678

The marked difference between the MM-GBSA and MM-PBSA totals demonstrates sensitivity of the calculated end-point energy to the implicit-solvent model. Accordingly, these values were interpreted as method-dependent end-point binding-energy estimates rather than experimentally measured binding free energies or quantitative evidence of ligand affinity.

#### Molecular dynamics simulation of the VEGFR1–8ST complex.

A 500 ns molecular dynamics simulation of VEGFR1 in complex with its co-crystallized ligand 8ST was analyzed to characterize the time-dependent conformational behavior of the receptor, ligand, and ATP-binding site. The trajectory was evaluated sequentially using RMSD, RMSF, radius of gyration, and solvent-accessible surface area analyses.

**RMSD analysis:** The RMSD profiles of the VEGFR1 protein, 8ST ligand, and active-site residues showed bounded but distinct conformational behavior during the 500 ns trajectory (Fig 10A). The protein RMSD initially fluctuated at approximately 0.12–0.17 nm and subsequently sampled values predominantly between 0.15 and 0.20 nm. A temporary increase to approximately 0.22 nm occurred around 180–220 ns, after which the protein RMSD returned to a narrower range of approximately 0.14–0.18 nm for most of the remaining trajectory. This pattern indicates an intermediate conformational adjustment rather than continuous structural divergence.

**Fig 10:**
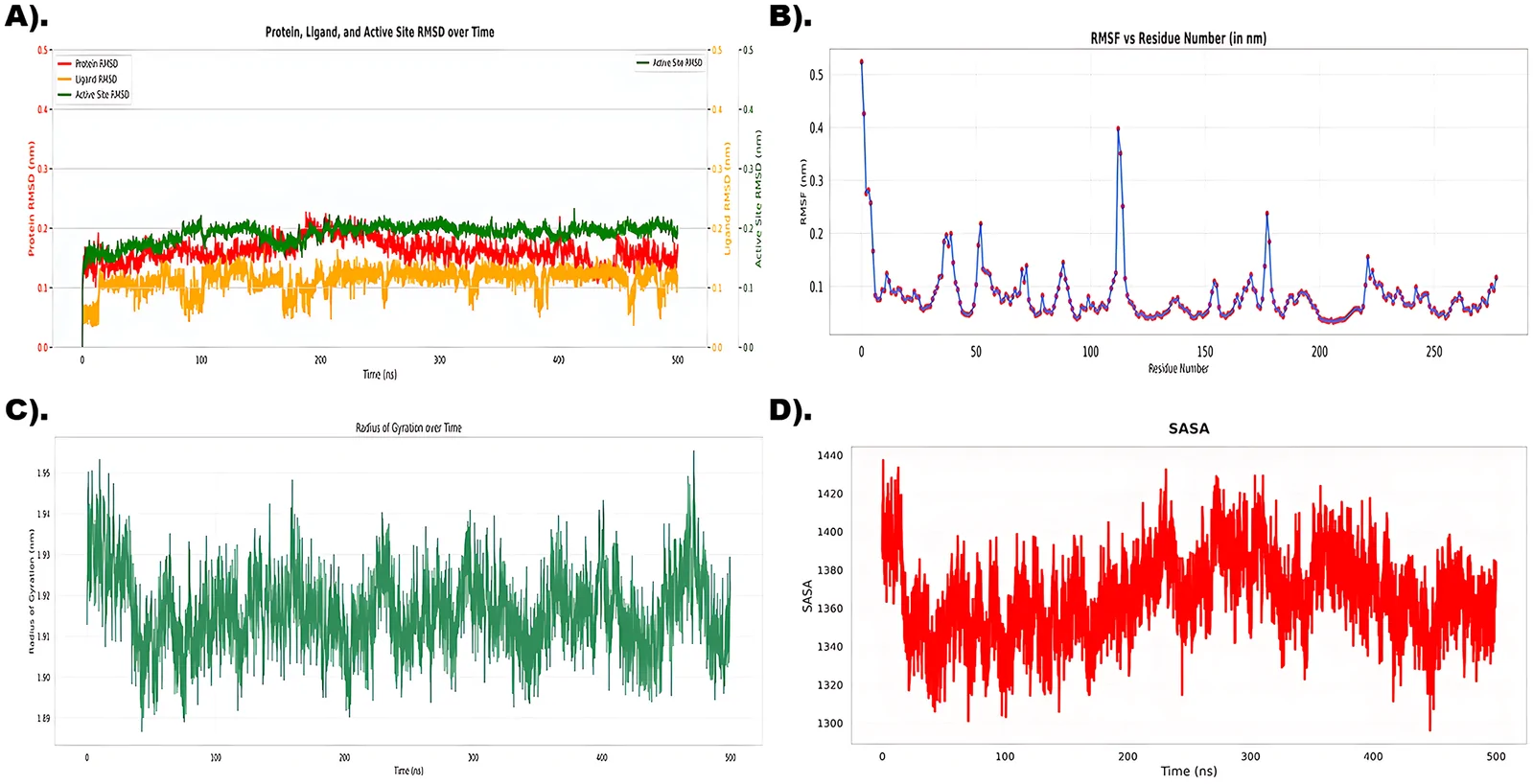
Structural dynamics of the VEGFR1–8ST complex during the 500 ns molecular dynamics simulation. (A). RMSD profiles of the VEGFR1 protein, co-crystallized ligand 8ST, and active-site residues. (B). Per-residue RMSF profile of VEGFR1 showing the distribution of localized residue-level fluctuations. (C). Radius-of-gyration profile describing the time-dependent global compactness of the receptor. (D). Solvent-accessible surface area profile showing changes in protein surface exposure during the trajectory.

The ligand RMSD increased from approximately 0.04–0.07 nm during the initial trajectory segment to approximately 0.10 nm within the first 20 ns. Thereafter, 8ST predominantly fluctuated between approximately 0.10 and 0.14 nm, with several short-lived downward excursions toward 0.05–0.09 nm. Importantly, the ligand trajectory did not show a sustained progressive increase in RMSD. The active-site RMSD gradually increased from approximately 0.13–0.16 nm during the initial segment and subsequently remained predominantly within approximately 0.18–0.21 nm. Collectively, these bounded RMSD profiles suggest that the VEGFR1–8ST system sampled receptor and binding-site conformational adjustments while retaining a comparatively restricted ligand positional deviation relative to the reference structure.

**RMSF analysis:** The residue-wise RMSF profile showed that most VEGFR1 residues fluctuated within approximately 0.04–0.10 nm during the 8ST-bound simulation (Fig 10B). Localized increases were observed in several discrete protein segments rather than across the complete receptor structure. The largest fluctuation occurred at the N-terminal region, where the maximum RMSF approached approximately 0.53 nm. Another pronounced peak of approximately 0.40 nm was observed around residue index 112, while secondary peaks of approximately 0.20–0.24 nm occurred near residue indices 38–40, 52, and 177. Additional smaller peaks generally remained below approximately 0.16 nm.

The concentration of higher RMSF values within a limited number of residue segments is consistent with localized mobility, particularly within terminal or loop-like regions, whereas most of the protein maintained comparatively low residue-level fluctuations. The RMSF profile therefore indicates that the 8ST-bound receptor retained a relatively constrained structural core while permitting localized conformational motion in selected regions.

**Radius of gyration analysis:** The radius of gyration of the VEGFR1–8ST complex remained within a narrow range of approximately 1.89–1.96 nm throughout the 500 ns trajectory, with most values centered near approximately 1.92 nm (Fig 10C). An initial decrease from approximately 1.93 nm to approximately 1.91 nm occurred during the early trajectory, followed by recurrent low-amplitude fluctuations around the central range. Short-lived increases approached approximately 1.95–1.96 nm, while temporary decreases reached approximately 1.89 nm.

No sustained upward or downward drift was observed across the trajectory. The bounded radius-of-gyration profile indicates that VEGFR1 maintained its overall global compactness during the 8ST-bound simulation, although small reversible changes in molecular packing occurred over time.

**Solvent-accessible surface area analysis:** The solvent-accessible surface area of the VEGFR1–8ST complex fluctuated approximately between 1300 and 1440 Å^2^ during the 500 ns simulation (Fig 10D). Higher values were observed during the initial trajectory segment, followed by a decrease toward approximately 1330–1380 Å^2^ during the early and intermediate portions of the simulation. The SASA subsequently increased during approximately 200–310 ns and sampled values frequently between approximately 1360 and 1410 Å^2^. Later portions of the trajectory continued to show reversible fluctuations without a sustained unidirectional trend.

These variations indicate time-dependent changes in protein surface exposure and molecular packing during the 8ST-bound trajectory. The absence of persistent progressive expansion or contraction suggests that the observed SASA changes represented conformational breathing and solvent-exposure rearrangements rather than continuous structural unfolding or collapse.

**Hydrogen-bond dynamics:** The intermolecular hydrogen-bond profile of the VEGFR1–8ST control complex varied from zero to six hydrogen bonds per frame during the 500 ns simulation (Fig 11A). The running-average profile was generally between approximately one and two hydrogen bonds, with intermittent increases approaching three hydrogen bonds. Periods of reduced hydrogen-bond occurrence were interspersed with recurrent contacts later in the trajectory. This pattern indicates intermittent polar interactions according to the applied hydrogen-bond definition; it should not be interpreted alone as a measure of binding affinity or complex stability.

**Fig 11:**
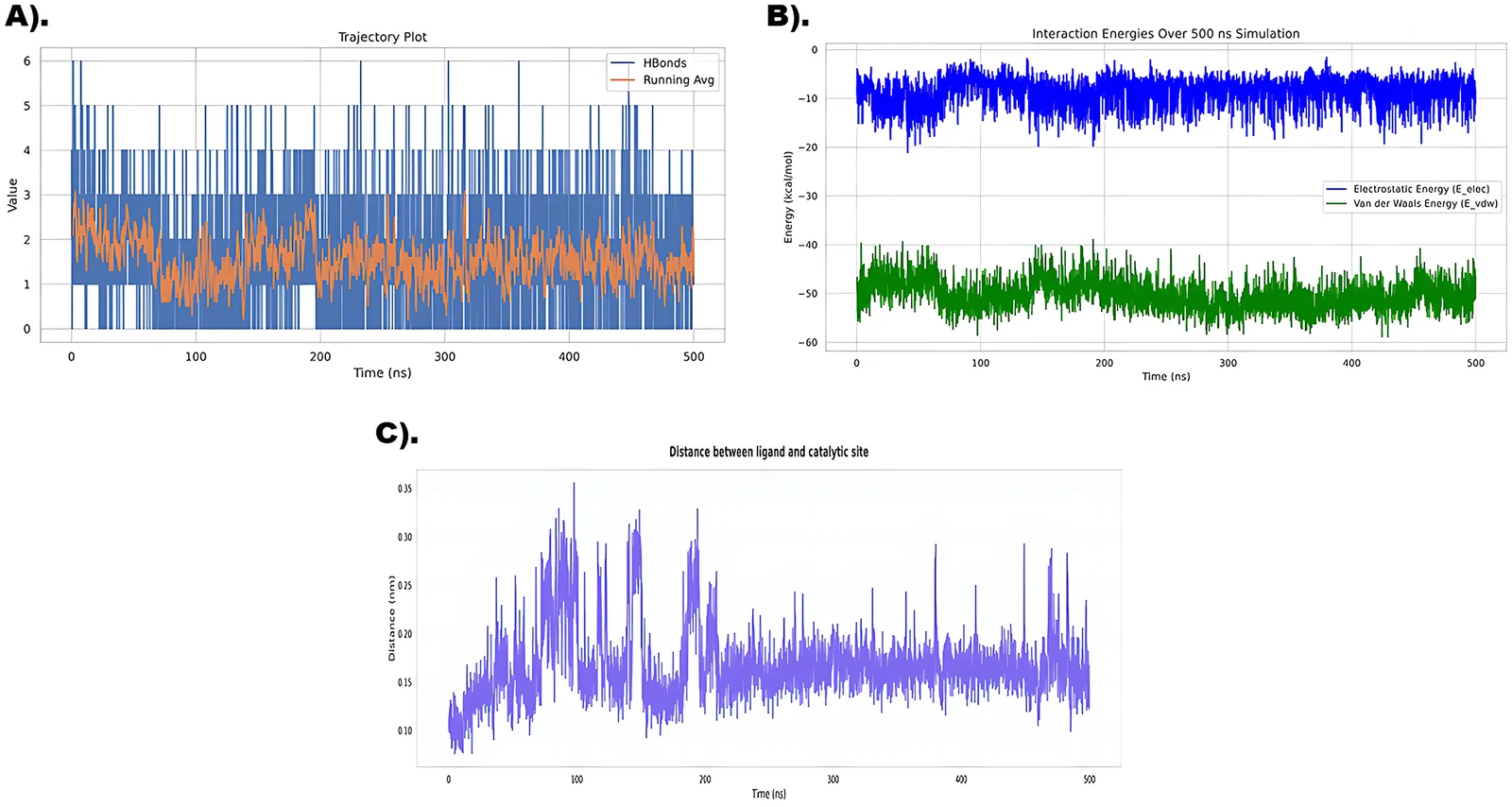
Time-dependent interaction behavior of the VEGFR1–8ST complex during the 500 ns molecular dynamics simulation. (A). Intermolecular hydrogen-bond count and 50-frame rolling-average profile obtained using the hydrogen-bond distance and angle criteria stated in the Methods. (B). Electrostatic and van der Waals interaction-energy components between VEGFR1 and 8ST, reported in kcal/mol. (C). Distance between 8ST and the VEGFR1 catalytic site during the simulation, reported in nm.

**Interaction-energy profile:** The electrostatic and van der Waals interaction-energy components between VEGFR1 and 8ST remained predominantly negative throughout the 500 ns trajectory (Fig 11B). The electrostatic contribution generally fluctuated between approximately −5 and −15 kcal/mol, with occasional short-lived excursions approaching approximately −20 kcal/mol. No sustained shift toward positive electrostatic-energy values was observed.

The van der Waals component was consistently more negative than the electrostatic component and generally fluctuated between approximately −45 and −55 kcal/mol. Brief excursions approached approximately −59 kcal/mol, whereas less-negative intervals remained near approximately −40 kcal/mol. The consistently negative profiles indicate favorable non-bonded interaction components under the simulated conditions, with van der Waals contacts providing the larger energetic contribution. These interaction energies describe direct non-bonded components and should not be interpreted as binding free energies because solvent, entropy, and other thermodynamic contributions are not included.

**Distance between 8ST and the catalytic site:** The measured distance between 8ST and the VEGFR1 catalytic site showed time-dependent positional rearrangements during the 500 ns trajectory (Fig 11C). During the initial approximately 20 ns, the distance remained near approximately 0.08–0.13 nm before increasing toward approximately 0.15–0.20 nm. Larger and more variable excursions occurred during approximately 70–100, 140–155, and 185–205 ns, with the maximum transient distance approaching approximately 0.36 nm.

After approximately 220 ns, the ligand–catalytic-site distance was predominantly maintained within approximately 0.13–0.20 nm. A small number of brief excursions reached approximately 0.25–0.29 nm, particularly during the later trajectory, but these increases were not sustained. The absence of progressive separation indicates that 8ST continued to sample positions near the catalytic region while undergoing localized positional adjustments. This distance profile supports retention within the catalytic-site region during the simulated trajectory but does not independently establish binding affinity.

### Principal component analysis

Principal component analysis (PCA) revealed a temporally organized redistribution of the VEGFR1–8ST trajectory across the conformational space defined by the first two principal components (Fig 12A). The early trajectory frames were predominantly distributed at positive PC1 and negative PC2 values. During the intermediate period, the sampled conformations extended toward negative PC1 values, indicating a transition between conformational regions. The later frames occupied an overlapping region closer to the central PC1–PC2 space, with comparatively greater representation at higher PC2 values. The overlap between successive time-colored regions indicates continuous conformational transitions rather than abrupt separation into completely independent states. Overall, the PCA profile demonstrates multistate conformational sampling followed by increased occupancy of a comparatively restricted conformational region during the later simulation period. However, in the absence of an apo-receptor simulation and replicate trajectories, this pattern should not be interpreted independently as evidence that 8ST stabilized the receptor.

**Fig 12:**
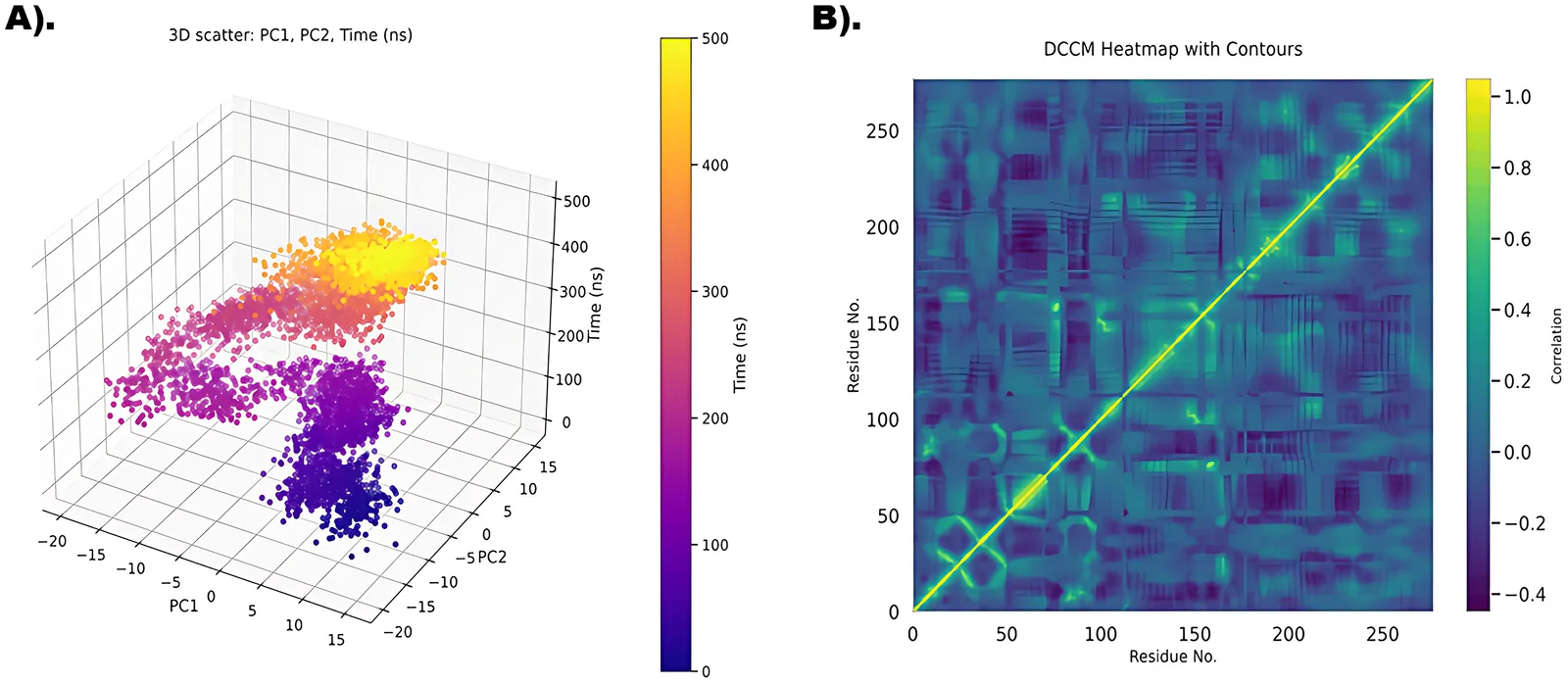
Principal component and dynamic cross-correlation analyses of the VEGFR1–8ST complex during the 500 ns molecular dynamics simulation. (A) Three-dimensional principal component analysis (PCA) projection showing the distribution of trajectory frames along PC1 and PC2, with simulation time represented by the vertical axis and color scale. (B) Dynamic cross-correlation matrix (DCCM) of VEGFR1 residue motions, where green-to-yellow regions represent positive correlations, blue regions indicate weak or near-zero correlations, purple regions represent anticorrelated motions, and the yellow diagonal represents residue self-correlation.

**Dynamic cross-correlation matrix analysis:** The dynamic cross-correlation matrix (DCCM) displayed heterogeneous patterns of correlated and anticorrelated residue motions throughout the VEGFR1 structure (Fig 12B). The strong positive correlation along the main diagonal represents the expected self-correlation of individual residues. Localized green-to-yellow off-diagonal regions indicate positively correlated motions between selected residue segments, whereas purple regions represent residues moving in opposing directions. Most off-diagonal regions exhibited weak-to-moderate correlations, demonstrating that the trajectory contained both coordinated local motions and long-range dynamic coupling. Thus, the DCCM profile indicates that VEGFR1 retained a dynamically coupled residue network during the 8ST-bound simulation, although these correlations do not by themselves establish a ligand-induced stabilizing effect.

**MM-GBSA and MM-PBSA binding-energy analyses of the VEGFR1–8ST control complex:** End-point energy decomposition was performed to estimate the binding energetics of the co-crystallized ligand, 8ST, within the VEGFR1 binding site. The MM-GBSA analysis produced a mean total binding-energy estimate of −49.53 ± 2.47 kcal/mol (mean ± standard deviation; Table 7). The gas-phase contribution was favorable at −70.39 kcal/mol and was dominated by the van der Waals contribution of −52.14 kcal/mol, together with an electrostatic contribution of −18.25 kcal/mol. The positive generalized Born polar-solvation term of 26.84 kcal/mol opposed complex formation, whereas the nonpolar surface contribution remained favorable at −5.98 kcal/mol. Consequently, the net solvation contribution was unfavorable at 20.86 kcal/mol but was insufficient to offset the favorable gas-phase interactions. These results indicate that the MM-GBSA estimate for 8ST was predominantly supported by van der Waals packing and favorable electrostatic interactions within the VEGFR1 binding site.

**Table 7:** MM-GBSA energy decomposition of the VEGFR1–8ST control complex calculated from the 500 ns trajectory. Values represent complex − receptor − ligand differences and are reported in kcal/mol.

Energy component	Average	Standard deviation	Standard error of mean
VDWAALS	−52.1414	2.0297	0.6419
EEL	−18.2454	5.6668	1.7920
EGB	26.8401	4.1349	1.3076
ESURF	−5.9794	0.1615	0.0511
DELTA G gas	−70.3868	6.0457	1.9118
DELTA G solv	20.8607	4.0720	1.2877
DELTA TOTAL	**−49.5261**	**2.4704**	**0.7812**

The MM-PBSA calculation yielded a less negative total binding-energy estimate of −9.98 ± 3.52 kcal/mol (mean ± standard deviation; Table 8). The favorable gas-phase contribution remained −70.39 kcal/mol because the same van der Waals and electrostatic components were used. However, this contribution was substantially opposed by a net solvation penalty of 60.41 kcal/mol. Within the solvation terms, the favorable nonpolar contribution of −30.15 kcal/mol was outweighed by the positive Poisson–Boltzmann polar-solvation and dispersion terms of 33.48 and 57.08 kcal/mol, respectively. The MM-PBSA calculation retained a negative mean total energy estimate; however, its substantially smaller magnitude relative to MM-GBSA demonstrates the strong dependence of the calculated end-point energy on the implicit-solvent treatment.

**Table 8:** MM-PBSA energy decomposition of the VEGFR1–8ST control complex calculated from the 500 ns trajectory. Values represent complex − receptor − ligand differences and are reported in kcal/mol.

Energy component	Average	Standard deviation	Standard error of mean
VDWAALS	−52.1414	2.0297	0.6419
EEL	−18.2454	5.6668	1.7920
EPB	33.4843	4.6456	1.4691
ENPOLAR	−30.1523	0.6029	0.1906
EDISPER	57.0784	1.2196	0.3857
DELTA G gas	−70.3868	6.0457	1.9118
DELTA G solv	60.4105	4.8291	1.5271
DELTA TOTAL	**−9.9763**	**3.5190**	**1.1128**

Compared with Emodin-8-glucoside, 8ST produced numerically more favorable total energy estimates with both methods. The MM-GBSA estimate for 8ST was −49.53 ± 2.47 kcal/mol, compared with −43.11 ± 4.92 kcal/mol for Emodin-8-glucoside, representing a difference of 6.42 kcal/mol in favor of the control. Similarly, the MM-PBSA estimate was −9.98 ± 3.52 kcal/mol for 8ST and −1.63 ± 9.70 kcal/mol for Emodin-8-glucoside, corresponding to a numerical difference of 8.35 kcal/mol. This behavior is reasonable for a co-crystallized reference ligand whose experimentally resolved pose demonstrates compatibility with the VEGFR1 binding site. Component-level comparison showed that Emodin-8-glucoside had a stronger electrostatic contribution, whereas 8ST exhibited more favorable van der Waals interactions and substantially smaller polar-solvation penalties. Thus, the overall energetic advantage of 8ST arose from a more favorable balance between intermolecular packing and desolvation rather than from electrostatic attraction alone. The control values provide an internally consistent reference for interpreting the computationally prioritized lead, but the numerical differences should not be regarded as statistically significant or equivalent to experimentally measured binding affinities.

### Comparative molecular dynamics analysis of Emodin-8-glucoside and the 8ST control

A side-by-side comparison was conducted to distinguish the dynamic and energetic behavior of the VEGFR1 complex containing the computationally prioritized lead, Emodin-8-glucoside, from that of the co-crystallized control ligand, 8ST. The comparison incorporated global protein behavior, residue flexibility, ligand retention, intermolecular interactions, collective conformational motions, and end-point binding-energy estimates obtained over the respective 500 ns trajectories (Table 9). The structural descriptors are presented in Figs 7A–D and 10A–D, the interfacial interaction profiles in Figs 8A–C and 11A–C, and the collective-motion analyses in Figs 9A–B and 12A–B.

**Table 9:** Side-by-side comparison of the 500 ns molecular dynamics and end-point binding-energy results for the VEGFR1 complexes with Emodin-8-glucoside and 8ST. Approximate values and ranges were obtained from the plotted profiles, whereas MM-GBSA and MM-PBSA totals are reported as mean ± standard deviation.

MD parameter	Emodin-8-glucoside–VEGFR1	8ST–VEGFR1 control
RMSD	Backbone RMSD ≈0.18 nm; ligand RMSD <0.15 nm; active-site RMSD ≈0.20 nm	Protein RMSD mainly ≈0.14–0.20 nm; ligand RMSD ≈0.10–0.14 nm; active-site RMSD ≈0.18–0.21 nm
RMSF	Most residues <0.15 nm; localized terminal and loop fluctuations ≈0.30–0.50 nm	Most residues ≈0.04–0.10 nm; localized terminal and loop peaks reached ≈0.53 nm
Radius of gyration	1.91–1.96 nm; average ≈1.93 nm	Approximately 1.89–1.96 nm; generally centered around ≈1.92 nm
SASA	Approximately 1320–1470 Å², without sustained directional drift	Approximately 1300–1440 Å²; predominantly 1340–1400 Å², without sustained directional drift
Intermolecular hydrogen bonds	Profile predominantly approximately 0.5–2.5; transient peaks approximately 3.5; 50-frame rolling mean approximately 0.7–2.2	Raw count 0–6; running average approximately 1–3
Interaction energies	van der Waals ≈−45 kcal/mol; electrostatic ≈−30 kcal/mol	van der Waals predominantly ≈−45 to −55 kcal/mol; electrostatic predominantly ≈−5 to −15 kcal/mol
Ligand–catalytic-site distance	Approximately 0.20–0.35 nm; centered at ≈0.28 nm after the initial adjustment	Approximately 0.08–0.35 nm during early sampling; predominantly ≈0.13–0.20 nm during the later trajectory
PCA	Multiple initial conformational populations followed by greater occupation of a comparatively compact region after ≈250 ns	Temporally ordered redistribution between conformational regions, followed by occupation of an overlapping later-state basin
DCCM	Moderate positive correlations of approximately 0.4–0.7 with localized anticorrelations around −0.3	Heterogeneous weak-to-moderate positive correlations with localized anticorrelated regions extending to approximately −0.4
MM-GBSA ΔTOTAL	−43.11 ± 4.92 kcal/mol	−49.53 ± 2.47 kcal/mol
MM-PBSA ΔTOTAL	−1.63 ± 9.70 kcal/mol	−9.98 ± 3.52 kcal/mol

The global structural descriptors showed that both complexes maintained bounded conformational behavior throughout the 500 ns simulations. Protein RMSD values remained within comparable ranges, while the ligand RMSD of 8ST was generally slightly lower than that of Emodin-8-glucoside. The active-site RMSD remained close to 0.20 nm in both systems, indicating that neither trajectory exhibited progressive structural displacement of the binding-site region. The RMSF profiles also showed relatively low fluctuations across most residues, although localized terminal and loop regions remained flexible in both complexes. The radius of gyration and SASA ranges substantially overlapped, suggesting that VEGFR1 retained comparable overall compactness and solvent exposure in both ligand-bound simulations (Figs 7A–D and 10A–D; Table 9). These observations support bounded receptor conformational behavior but do not independently demonstrate that either ligand stabilized VEGFR1 relative to an unbound receptor.

The interfacial analyses revealed different interaction patterns for the lead and control complexes. Emodin-8-glucoside showed recurrent hydrogen-bond interactions and a more negative direct electrostatic component, whereas 8ST showed more favorable van der Waals contributions. Hydrogen-bond counts were interpreted descriptively and were not used to rank binding strength. This electrostatic difference is chemically reasonable because the glycosylated structure of Emodin-8-glucoside contains more hydrogen-bond donors and acceptors than 8ST. However, the number of hydrogen bonds should not be interpreted as a direct measure of binding strength because it depends on ligand size, chemical composition, geometric criteria, and interaction persistence. Conversely, 8ST showed comparable or more favorable van der Waals interactions, consistent with effective steric and hydrophobic complementarity within the crystallographic binding site (Figs 8A–B and 11A–B; Table 9).

Both ligands remained spatially associated with the catalytic-site region, although their centroid-distance profiles differed. Emodin-8-glucoside was predominantly positioned at approximately 0.28 nm from the catalytic-site reference after its initial adjustment, whereas 8ST generally sampled a shorter distance of approximately 0.13–0.20 nm during the later trajectory (Figs 8C and 11C). The shorter distance observed for 8ST is consistent with its crystallographic binding geometry. Nevertheless, centroid-based distances are influenced by ligand dimensions, orientation, and the atoms used to define the catalytic-site center; therefore, they should be used to assess positional retention rather than to rank binding affinity directly.

PCA demonstrated that both complexes sampled multiple conformational regions before showing greater occupation of later-state conformational basins. Emodin-8-glucoside showed a comparatively compact late-stage population after approximately 250 ns, whereas the 8ST trajectory displayed temporally ordered redistribution into an overlapping later conformational region (Figs 9A and 12A). DCCM analysis showed that both systems retained networks of correlated and anticorrelated residue motions, with differences in the distribution and magnitude of individual correlation regions (Figs 9B and 12B). These analyses indicate distinct patterns of conformational sampling and residue coupling but cannot establish ligand-induced receptor stabilization without corresponding apo-receptor and replicate simulations.

The end-point energy calculations numerically favored the co-crystallized control. The MM-GBSA total was 6.42 kcal/mol more negative for 8ST than for Emodin-8-glucoside, while the MM-PBSA total was 8.35 kcal/mol more negative for 8ST (Tables 5–8). Given the standard deviations associated with these estimates and the small number of snapshots from which they were derived, the two numerical differences are not formally distinguishable from the spread of the data, and they are therefore interpreted only as a consistent directional trend favoring the crystallographic control rather than as a quantified energetic separation. Component decomposition showed that Emodin-8-glucoside possessed a stronger favorable electrostatic contribution, but this advantage was substantially opposed by larger polar-solvation penalties. In contrast, 8ST combined more favorable van der Waals interactions with lower desolvation costs, producing a more favorable overall energetic balance. The near-zero MM-PBSA estimate for Emodin-8-glucoside, together with its comparatively large standard deviation, indicates uncertainty under the Poisson–Boltzmann model and should not be presented as evidence of high affinity. Overall, Emodin-8-glucoside displayed global MD behavior broadly comparable to that of the crystallographic control and maintained persistent polar interactions, whereas 8ST remained the energetically stronger reference under both end-point models. These findings support Emodin-8-glucoside as a computationally prioritized candidate for experimental testing but do not demonstrate that it binds more strongly than the co-crystallized ligand.

### Pharmacophore characterization and DFT of Emodin-8-glucoside

Pharmacophore and electronic-structure analyses were performed to characterize the molecular features and quantum-mechanical properties of Emodin-8-glucoside. Pharmit identified hydrogen-bond donor, hydrogen-bond acceptor, aromatic, and hydrophobic features within the molecular structure (Fig 13A). The spatial distribution of these pharmacophoric elements defines interaction-capable regions of Emodin-8-glucoside that may contribute to molecular recognition.

**Fig 13:**
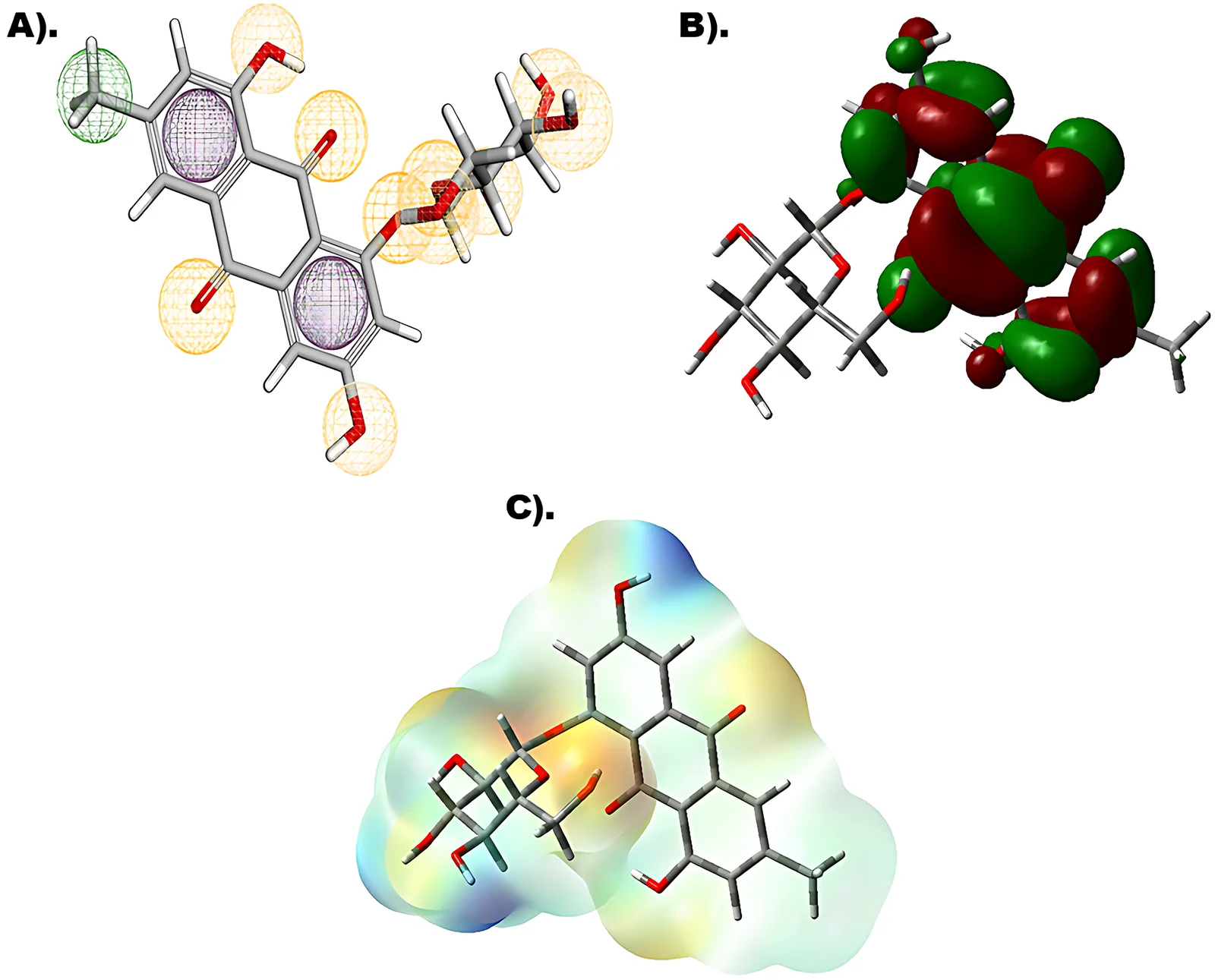
Pharmacophore and electronic-structure characterization of Emodin-8-glucoside. (A) Pharmacophore mapping showing hydrogen-bond donor, hydrogen-bond acceptor, aromatic, and hydrophobic features. (B) HOMO and LUMO orbital distributions obtained from the DFT calculation, with a calculated HOMO-LUMO energy gap of 0.13204 Hartree (approximately 3.59 eV). (C) Molecular electrostatic potential surface showing comparatively electron-rich and electron-deficient molecular regions.

The calculated HOMO and LUMO energies of Emodin-8-glucoside were −0.23991 and −0.10787 Hartree, respectively, corresponding to approximately −6.53 and −2.94 eV. The HOMO-LUMO energy gap, calculated as the difference between the LUMO and HOMO energies, was 0.13204 Hartree, equivalent to approximately 3.59 eV (Fig 13B). These values characterize the frontier-orbital energy separation of Emodin-8-glucoside under the applied DFT model and were not interpreted as direct measures of VEGFR1 binding affinity or biological activity.

The molecular electrostatic potential surface showed comparatively electron-rich regions around the oxygen-containing groups and electron-deficient regions around selected hydrogen atoms (Fig 13C). This distribution identifies potential sites for electrostatic and hydrogen-bond interactions but does not demonstrate that these interactions occur with particular VEGFR1 residues.

### ADMET analysis of Emodin-8-glucoside

SwissADME predicted a molecular weight of 432.38 g/mol, 10 hydrogen-bond acceptors, six hydrogen-bond donors, three rotatable bonds, a topological polar surface area (TPSA) of 173.98 Å^2^, and a consensus log P value of −0.07 for Emodin-8-glucoside (Table 10). The compound received a Lipinski classification of “Yes” and a predicted bioavailability score of 0.55. However, gastrointestinal absorption was predicted to be low, consistent with its high polar surface area, and Emodin-8-glucoside was predicted to be a P-glycoprotein substrate (Fig 14). Blood-brain barrier permeation was predicted to be absent (Fig 14). No inhibition was predicted for CYP1A2, CYP2C19, CYP2C9, CYP2D6, or CYP3A4. These results indicate substantial polarity and limited predicted gastrointestinal absorption despite its drug-likeness classification.

**Table 10:** SwissADME-predicted physicochemical, pharmacokinetic, and drug-likeness properties of Emodin-8-glucoside including lipophilicity, TPSA, solubility, and CYP inhibition profile.

ADME Parameters	
Physicochemical properties	
Formula	C21H20O10
Molecular weight	432.38 g/mol
Num. heavy atoms	31
Num. arom. heavy atoms	12
Fraction Csp3	0.33
Num. rotatable bonds	3
Num. H-bond acceptors	10
Num. H-bond donors	6
Molar Refractivity	102.91
TPSA (Å^2^)	173.98 Å²
Lipophilicity	
Log Po/w	−0.07
Water Solubility	
Log *S* (ESOL)	−3.18
Class	Soluble
Pharmacokinetics	
GI absorption	Low
BBB permeant	No
P-gp substrate	Yes
CYP1A2 inhibitor	No
CYP2C19 inhibitor	No
CYP2C9 inhibitor	No
CYP2D6 inhibitor	No
CYP3A4 inhibitor	No
Log *K*_p_ (skin permeation)	−8.29 cm/s
Drug Likeness	
Lipinski	Yes
Bioavailability Score	0.55

**Fig 14:**
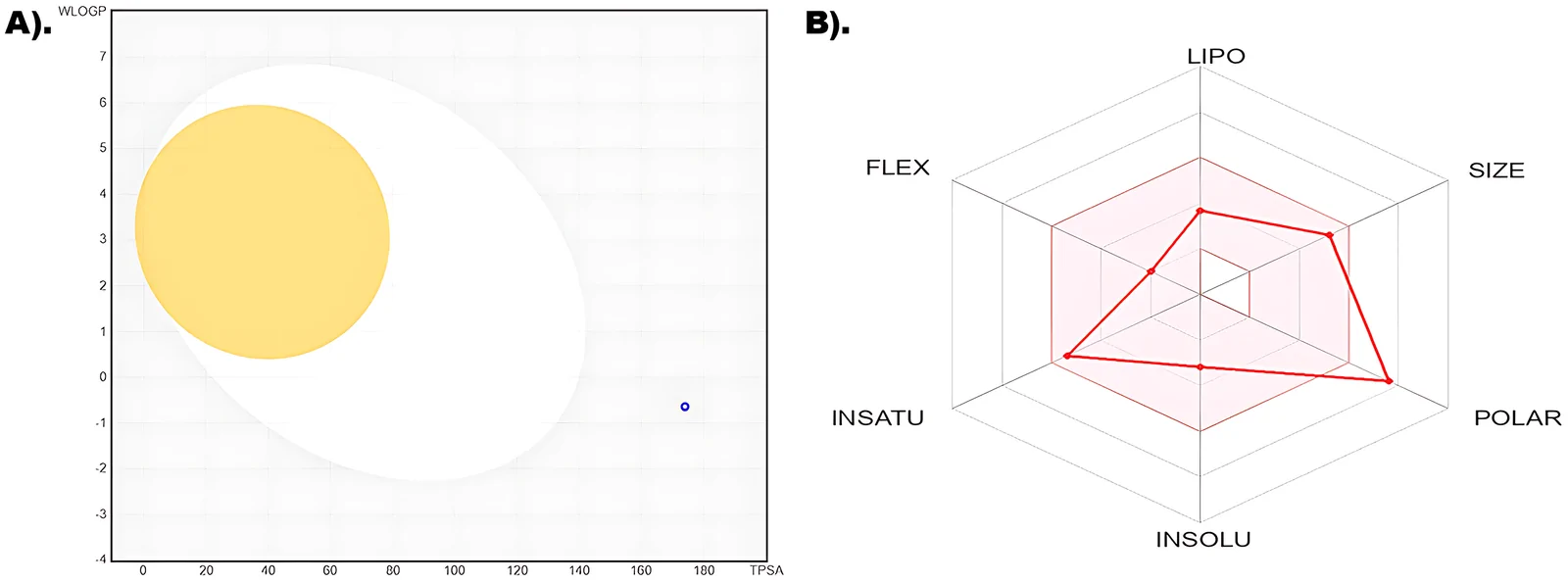
Predicted pharmacokinetic characteristics of Emodin-8-glucoside obtained using SwissADME. (A) BOILED-Egg representation of the compound according to lipophilicity and topological polar surface area. SwissADME predicted low gastrointestinal absorption and no blood-brain barrier permeation for Emodin-8-glucoside. (B) Graphical summary of physicochemical and pharmacokinetic properties predicted by SwissADME.

ProTox 3.0 assigned Emodin-8-glucoside to acute oral toxicity class 5, with an estimated LD50 of 3000 mg/kg (Table 11). The toxicity predictions showed a mixed profile. Nephrotoxicity was predicted active with a probability of 0.75, respiratory toxicity with a probability of 0.57, and cardiotoxicity with a probability of 0.76. Among the general toxicity endpoints, immunotoxicity and mutagenicity were predicted active with probabilities of 0.92 and 0.91, respectively, while clinical toxicity and nutritional toxicity were predicted active with probabilities of 0.62 and 0.54, respectively. In contrast, hepatotoxicity, neurotoxicity, carcinogenicity, and cytotoxicity were predicted inactive. Among the molecular initiating events, transthyretin (TTR) was the only endpoint predicted active, with a probability of 0.88. These model-based classifications identify potential toxicity liabilities requiring experimental evaluation. Teratogenicity, embryotoxicity, reproductive toxicity, fetal toxicity, placental transfer, and other pregnancy-specific safety endpoints were not evaluated.

**Table 11:** Toxicity classification of Emodin-8-glucoside from ProTox 3.0, summarizing organ toxicity, stress response pathways, nuclear receptor signaling, and molecular initiating event predictions.

Classification	Target	Prediction	Probability
Organ toxicity	Hepatotoxicity	Inactive	0.85
Organ toxicity	Neurotoxicity	Inactive	0.87
Organ toxicity	Nephrotoxicity	Active	0.75
Organ toxicity	Respiratory toxicity	Active	0.57
Organ toxicity	Cardiotoxicity	Active	0.76
Toxicity end points	Carcinogenicity	Inactive	0.84
Toxicity end points	Immunotoxicity	Active	0.92
Toxicity end points	Mutagenicity	Active	0.91
Toxicity end points	Cytotoxicity	Inactive	0.77
Toxicity end points	BBB-barrier	Inactive	0.57
Toxicity end points	Ecotoxicity	Inactive	0.68
Toxicity end points	Clinical toxicity	Active	0.62
Toxicity end points	Nutritional toxicity	Active	0.54
Tox21-Nuclear receptor signaling pathways	Aryl hydrocarbon Receptor (AhR)	Inactive	0.90
Tox21-Nuclear receptor signaling pathways	Androgen Receptor (AR)	Inactive	0.95
Tox21-Nuclear receptor signaling pathways	Androgen Receptor Ligand Binding Domain (AR-LBD)	Inactive	0.97
Tox21-Nuclear receptor signaling pathways	Aromatase	Inactive	0.98
Tox21-Nuclear receptor signaling pathways	Estrogen Receptor Alpha (ER)	Inactive	0.94
Tox21-Nuclear receptor signaling pathways	Estrogen Receptor Ligand Binding Domain (ER-LBD)	Inactive	0.99
Tox21-Nuclear receptor signaling pathways	Peroxisome Proliferator Activated Receptor Gamma (PPAR-Gamma)	Inactive	0.97
Tox21-Stress response pathways	Nuclear factor (erythroid-derived 2)-like 2/ antioxidant response element (ARE)	Inactive	0.97
Tox21-Stress response pathways	Heat shock factor response element (HSE)	Inactive	0.97
Tox21-Stress response pathways	Mitochondrial Membrane Potential (MMP)	Inactive	0.86
Tox21-Stress response pathways	Phosphoprotein (Tumor Suppressor) p53	Inactive	0.58
Tox21-Stress response pathways	ATPase family AAA protein 5 (ATAD5)	Inactive	0.99
Molecular Initiating Events	Thyroid hormone receptor alpha (THRα)	Inactive	0.64
Molecular Initiating Events	Thyroid hormone receptor beta (THRβ)	Inactive	0.74
Molecular Initiating Events	Transthyretin (TTR)	Active	0.88
Molecular Initiating Events	Ryanodine receptor (RYR)	Inactive	0.86
Molecular Initiating Events	GABA receptor (GABAR)	Inactive	0.67
Molecular Initiating Events	Glutamate NMDA receptor (NMDAR)	Inactive	0.99
Molecular Initiating Events	AMPA receptor (AMPAR)	Inactive	1.00
Molecular Initiating Events	Kainate receptor (KAR)	Inactive	1.00
Molecular Initiating Events	Acetylcholinesterase (AChE)	Inactive	0.60
Molecular Initiating Events	Constitutive androstane receptor (CAR)	Inactive	1.00
Molecular Initiating Events	Pregnane X receptor (PXR)	Inactive	0.62
Molecular Initiating Events	NADH-quinone oxidoreductase (NADHOX)	Inactive	0.8
Molecular Initiating Events	Voltage gated sodium channel (VGSC)	Inactive	0.93
Molecular Initiating Events	Na+/I- symporter (NIS)	Inactive	0.85
Metabolism	Cytochrome CYP1A2	Inactive	0.96
Metabolism	Cytochrome CYP2C19	Inactive	0.98
Metabolism	Cytochrome CYP2C9	Inactive	0.73
Metabolism	Cytochrome CYP2D6	Inactive	0.92
Metabolism	Cytochrome CYP3A4	Inactive	0.95
Metabolism	Cytochrome CYP2E1	Inactive	0.98

The *in silico* ADMET profile indicates notable pharmacokinetic and toxicity limitations for Emodin-8-glucoside. The high TPSA, low predicted gastrointestinal absorption, and P-glycoprotein substrate status may limit systemic exposure, while the active toxicity predictions identify several endpoints requiring further investigation. These computational predictions provide preliminary safety and pharmacokinetic characterization and require experimental validation.

## Discussion

Placenta previa is defined by placental implantation over or near the internal cervical os and is clinically distinct from placenta accreta spectrum (PAS), preeclampsia, and fetal growth restriction [1–7]. Although placenta previa may coexist with PAS, PAS is characterized by abnormal placental adherence or invasion into the uterine wall [2,4]. These distinctions are important because angiogenic observations from one obstetric condition cannot be directly extrapolated to another. Placenta-previa tissue studies have reported alterations in VEGF expression, inflammatory signaling, and vascular morphology, suggesting disturbed angiogenic regulation [10]. However, these observations do not establish increased kinase activity of membrane-bound VEGFR1 or demonstrate that pharmacological VEGFR1 inhibition would improve placenta previa. Accordingly, VEGFR1 was investigated here as an exploratory structure-based target within the angiogenic context of placenta previa rather than as an established therapeutic target.

VEGFR1 exists as full-length membrane-bound forms containing an intracellular tyrosine-kinase domain and as soluble sFlt-1 variants lacking the transmembrane and intracellular kinase regions [8,9]. Consequently, the ATP-binding pocket examined in the present study pertains to membrane-bound VEGFR1 signaling rather than the ligand-sequestering function of soluble sFlt-1. The computationally predicted kinase-domain interaction therefore cannot be interpreted as direct modulation of VEGF or PlGF sequestration by sFlt-1.

The present computational workflow prioritized Emodin-8-glucoside as a candidate capable of occupying the ATP-binding pocket of the intracellular VEGFR1 kinase domain. Emodin-8-glucoside ranked highly in both AutoDock Vina and MOE, although the numerical differences from several other compounds were small and the two programs showed only moderate rank concordance (Table 2). Docking was performed using a rigid-receptor approximation and therefore represents an initial pose-generation and prioritization step rather than a measurement of binding affinity. Redocking of the co-crystallized ligand 8ST reproduced its crystallographic orientation with a symmetry-corrected heavy-atom RMSD of 1.465 Å, supporting the pose-reproduction capability of the applied docking protocol. Subsequent molecular dynamics simulations showed that both Emodin-8-glucoside and the 8ST control remained associated with the VEGFR1 binding-site region during their respective 500 ns trajectories (Figs 7–12; Table 9). The two complexes displayed distinct interaction and conformational-sampling profiles, but these trajectory descriptors describe persistence of the modeled complexes and do not independently establish inhibitory potency or ligand-induced stabilization of VEGFR1.

End-point energy analysis also showed that interpretation depended strongly on the implicit-solvent model. For Emodin-8-glucoside, MM-GBSA yielded a mean total energy estimate of −43.11 ± 4.92 kcal/mol, whereas MM-PBSA produced a near-zero estimate of −1.63 ± 9.70 kcal/mol (Tables 5 and 6). The comparatively large variability of the MM-PBSA result prevents it from being regarded as independent confirmation of favorable binding. The co-crystallized 8ST control produced more negative mean estimates under both approaches, with MM-GBSA and MM-PBSA values of −49.53 ± 2.47 and −9.98 ± 3.52 kcal/mol, respectively (Tables 7 and 8). Component decomposition indicated that Emodin-8-glucoside benefited from stronger electrostatic contributions but incurred larger polar-solvation penalties, whereas 8ST showed a more favorable balance of van der Waals interactions and desolvation. The divergence between the GB and PB estimates emphasizes that these calculations should be interpreted as method-dependent end-point energy estimates rather than experimentally determined binding free energies.

Emodin-8-glucoside is a glycosylated anthraquinone related to emodin, a natural anthraquinone investigated in several non-obstetric biological systems [14–18]. Experimental studies have reported angiogenesis-related effects of emodin, including modulation of VEGF-receptor signaling and angiogenic processes in cancer-derived experimental models [19,59]. These observations provide biological context for investigating this chemical class but should not be treated as direct evidence that Emodin-8-glucoside inhibits VEGFR1. Differences in glycosylation, molecular structure, cellular context, receptor expression, and experimental conditions can substantially alter biological activity. The present structure-based results therefore extend the computational characterization of Emodin-8-glucoside but do not confirm anti-angiogenic activity, VEGFR1 inhibition, or modulation of placental angiogenesis. Direct biochemical and cellular experiments are required to determine whether the predicted kinase-pocket interaction translates into measurable VEGFR1 inhibition.

Pharmacological manipulation of VEGF-family signaling is well established in oncology and ophthalmology, including through agents that directly or indirectly inhibit VEGF-dependent angiogenic signaling [59–61]. Natural products and flavonoids have also been investigated experimentally as modulators of angiogenesis and VEGF-associated pathways [59–62]. However, these therapeutic and experimental contexts differ substantially from pregnancy. Natural origin does not provide evidence of superior maternal-fetal safety, and the present study does not establish that Emodin-8-glucoside is safer than conventional anti-angiogenic agents. Its prioritization is based on computational structural and molecular-property analyses and should therefore be regarded as hypothesis-generating.

Findings from preeclampsia and PAS illustrate the complexity and disease-specific nature of placental angiogenic regulation rather than providing direct therapeutic support for VEGFR1 inhibition in placenta previa. Alterations in VEGFR-family expression have been reported across normal and complicated pregnancies, while excessive placental sFlt-1 production is an established feature investigated in preeclampsia-related experimental systems, including studies evaluating regulation of placental sFlt-1 release [9,63]. PAS, in contrast, is characterized by abnormal placental adherence and invasion and involves a distinct pathological context [1,64]. These conditions therefore should not be combined under a single assumption of excessive VEGFR1 signaling. In particular, the present computational analysis does not demonstrate that membrane-bound VEGFR1 kinase activity is elevated in placenta previa. Experimental characterization of VEGFR1 phosphorylation and downstream signaling in disease-relevant placental models will be necessary before the biological significance of kinase-domain inhibition can be established.

The SwissADME and ProTox 3.0 predictions identify important pharmacokinetic and toxicological constraints that must be considered alongside the structural results. Emodin-8-glucoside showed a high topological polar surface area of 173.98 Å^2^, low predicted gastrointestinal absorption, and P-glycoprotein substrate behavior, indicating that oral systemic exposure may be limited (Table 10). ProTox 3.0 predicted active mutagenicity, immunotoxicity, nephrotoxicity, cardiotoxicity, respiratory toxicity, clinical-toxicity, and nutritional-toxicity endpoints, as well as transthyretin interaction (Table 11). These alerts do not demonstrate that the compound will produce the corresponding toxicities experimentally, but they preclude describing the computational safety profile as uniformly favorable. Moreover, teratogenicity, embryotoxicity, reproductive toxicity, fetal toxicity, placental transfer, and other pregnancy-specific outcomes were not evaluated. Consequently, the present ADMET predictions cannot establish maternal-fetal safety or suitability for administration during pregnancy.

Although the *in-silico* results are encouraging, it should be noted that our study has an exploratory nature and a number of limitations. First, all findings constitute computational predictions; we did not conduct in-vitro or in-vivo experiments to confirm that Emodin-8-glucoside indeed binds to VEGFR1 or modulates angiogenic signaling in placental cells. While computational docking and molecular dynamics are powerful tools, they remain approximations, and biological activity in vivo is influenced by factors not captured by these models, including the metabolic stability of the compound, its bioavailability to the uteroplacental unit and the complexity of cell-signaling interactions in vivo. Second, the use of an anti-angiogenic strategy during pregnancy raises important safety concerns. VEGF–VEGFR1 signaling is crucial for normal placental and fetal vascular development; excessive inhibition could jeopardize placental function or induce fetal growth restriction. Any therapeutic application would therefore involve identifying a safe therapeutic window, possibly through a localized or short-term intervention that reduces pathology without disrupting normal angiogenesis.

Only one 500 ns molecular dynamics trajectory was generated for each ligand-receptor complex, and independent replicate simulations with different initial velocity seeds were not performed. Consequently, the observed RMSD, RMSF, hydrogen-bond, interaction-energy, PCA, DCCM, and end-point energy profiles may partly reflect trajectory-specific conformational sampling. Although the co-crystallized 8ST complex provided a methodologically consistent reference for comparison, it does not substitute for independent replicate simulations. A further limitation concerns the end-point energy estimates, which were averaged over only ten snapshots taken at 50 ns intervals. Although this spacing makes appreciable correlation between successive snapshots unlikely, no block averaging or convergence analysis was carried out, so the reported standard deviations and standard errors should be read as the spread across a small sample rather than as converged uncertainty estimates. For the same reason, the numerical differences between the lead compound and the crystallographic control are not treated as formally distinguishable values. Future studies should perform multiple independently initialized MD simulations for each system to assess reproducibility, convergence, and the robustness of the observed conformational and energetic trends.

In future work, experimental validation should proceed in a stepwise manner to determine whether the predicted kinase-domain interaction has measurable biological consequences. First, a biochemical VEGFR1 kinase assay should be performed across a concentration range of Emodin-8-glucoside to generate a dose-response curve and determine an experimental inhibitory concentration, with a validated VEGFR1 inhibitor included as a positive control. A parallel VEGFR2 kinase counter-screen would be required to determine whether any observed activity shows selectivity for VEGFR1 rather than reflecting broader VEGFR-family kinase inhibition. Candidate concentrations supported by the biochemical assays should then be evaluated in placental trophoblast and endothelial cell models. These experiments should assess VEGFR1 phosphorylation, relevant downstream signaling responses, cell viability, proliferation, migration, and angiogenesis-related phenotypes. Such studies would establish whether the computationally predicted binding mode corresponds to target engagement and functional activity in biologically relevant systems.

## Conclusion

This study computationally prioritized Emodin-8-glucoside as an exploratory candidate for interaction with the intracellular kinase domain of VEGFR1 in the context of placenta previa. The integrated docking, molecular dynamics, end-point energy, and molecular-property analyses supported its selection for further investigation, while comparison with the co-crystallized 8ST control provided an internal reference for interpreting the computational findings. However, the present results do not establish VEGFR1 inhibition, therapeutic efficacy, selectivity, or pregnancy safety. Experimental validation through VEGFR1 kinase assays, VEGFR2 counter-screening, and relevant placental cell models is required to determine the biological significance of the predicted interaction.

## Supporting information

S1 Table List of twenty-one phytochemicals retrieved from the PubChem database with corresponding PubChem CIDs, 3D structures, and common plant sources used for VEGFR1 screening. (DOCX)
