# Supplementary material for: Structure-based computational prioritization of Emodin-8-glucoside as a candidate ligand for the VEGFR1 kinase domain in the context of placenta previa
Source: PLoS One. 2026 Oct 15;21(10):e0345201. doi: 10.1371/journal.pone.0345201 (PMC13671540; doi:10.1371/journal.pone.0345201)
Supplement: Supplementary file 1 [file pone.0345201.s001.docx]

**Table S1.** List of twenty-one phytochemicals retrieved from the PubChem database with corresponding PubChem CIDs, 3D structures, and common plant sources used for VEGFR1 screening.

| No. | Compound | PubChem CID | 3D Structures | Plant Source (common) |
| --- | --- | --- | --- | --- |
| 1. | Apigenin | 5280443 |  | *Parsley*, *celery*, *chamomile flowers* |
| 2. | Apigenin 7-O-glucoside | 5280704 |  | *Matricaria chamomilla*, *Apium graveolens*, *Petroselinum crispum* |
| 3. | Baicalein | 5281605 |  | *Scutellaria baicalensis* (Baikal skullcap) |
| 4. | Berberine | 2353 |  | *Berberis vulgaris* (barberry), *Mahonia aquifolium*, *Hydrastis canadensis*, *Coptis chinensis* |
| 5. | Beta Sitosterol | 222284 |  | Soybean, Avocado |
| 6. | Caffeic Acid Phenethyl Ester | 5281787 |  | A component of bee propolis (plant resin collected from various flowering plants) |
| 7. | Curcumin | 969516 |  | *Curcuma longa* (turmeric) |
| 8. | Daidzein | 5281708 |  | Soybean, *Cicer arietinum* |
| 9. | Ellagic acid | 5281855 |  | Pomegranate, strawberries, raspberries, walnuts |
| 10. | Emodin-8-glucoside | 99649 |  | Roots/rhizomes of *Rheum palmatum*, also *Rumex*, *Aloe vera* |
| 11. | Epigallocatechin | 72277 |  | *Green tea* (*Camellia sinensis*) leaves |
| 12. | Fisetin | 5281614 |  | *Strawberries*, *apples*, *grapes*, *onions*, *cucumbers* |
| 13. | Gallic Acid | 370 |  | Present in many fruits and herbs — e.g., gall nuts, tea, grapes, blueberries, *Eucalyptus bark* |
| 14. | Genistein | 5280961 |  | *Soy* (*Glycine max*), chickpeas, other legumes |
| 15. | Kaempferol | 5280863 |  | Found in kale, beans, tea, broccoli, grapefruit |
| 16. | Luteolin | 5280445 |  | *Parsley*, *celery*, *peppers*, *chamomile*, *thyme* |
| 17. | Quercetin | 5280343 |  | *Apples*, *onions*, *berries*, *capers*, *tea* |
| 18. | Resveratrol | 445154 |  | *Red grapes*, *peanuts*, *berries*, *Japanese knotweed* |
| 19. | Rosmarinic acid | 5281792 |  | *Rosmarinus officinalis* (rosemary) |
| 20. | Rutin | 5280805 |  | *Ruta graveolens* (rue) |
| 21. | Wogonin | 5281703 |  | *Scutellaria baicalensis*, *Scutellaria lateriflora* |
| Control Benchmarks | | | | |
| 22. | Co-crystallized ligand 8ST (N-(4-chlorophenyl)-2-[(pyridin-4-ylmethyl)amino]benzamide) | 9797919 |  | Synthetic |
| 23. | Lenvatinib | 9823820 |  | Synthetic |
| 24. | Sorafenib | 216239 |  | Synthetic |
