# Supplementary material for: Structure-based computational prioritization of Emodin-8-glucoside as a candidate ligand for the VEGFR1 kinase domain in the context of placenta previa
Source: PLoS One. 2026 Oct 15;21(10):e0345201. doi: 10.1371/journal.pone.0345201 (PMC13671540; doi:10.1371/journal.pone.0345201)
Supplement: Supplementary file 2 [file pone.0345201.s003.docx]

Dear Editor and Reviewers,

We sincerely thank the Editor and all Reviewers for their careful evaluation of our manuscript and for providing constructive and insightful comments. Their suggestions have helped us substantially improve the scientific rigor, clarity, methodological transparency, and interpretation of the study. We have carefully considered each comment and revised the manuscript accordingly, including the biological rationale, structural selection and validation, docking methodology, molecular dynamics analysis, end-point energy interpretation, ADMET assessment, terminology, and overall framing of the conclusions. All changes have been incorporated into the revised manuscript, and detailed point-by-point responses are provided below. We greatly appreciate the time and effort invested by the Editor and Reviewers in helping us strengthen the manuscript.

**Reviewer #1:**

1. The selection of the VEGFR1 crystal structure (PDB ID: 3HNG) was attributed to its high-resolution crystallographic data; however, the manuscript does not report the exact resolution value of the structure. Furthermore, the authors did not provide sufficient justification regarding the biological relevance of the selected conformation, including whether 3HNG represents the active or inactive state of VEGFR1, or whether the structure contains co-crystallized ligands, mutations, or missing residues that could influence docking accuracy and ligand-binding predictions. Considering the availability of multiple VEGFR1 structures within the Protein Data Bank, a more comprehensive rationale for the selection of 3HNG is warranted. To strengthen this section, the authors should explicitly report the crystallographic resolution of the selected structure and provide a comparative justification against other available VEGFR1 crystal structures. In addition, clarification regarding the conformational state and structural relevance of 3HNG for ligand-binding investigations would improve the scientific validity of the docking protocol.

**Response: Thank you for this important comment. We have expanded the structural-selection section to provide a detailed rationale for using PDB ID 3HNG. The revised manuscript now reports its X-ray resolution of 2.70 Å, identifies the co-crystallized inhibitor 8ST, confirms the absence of annotated mutations in the aligned VEGFR1 sequence, and specifies the modeled and unresolved residues. We also clarified that 3HNG adopts a DFG-out/alphaC-helix-in inactive-like kinase conformation. Representative alternative VEGFR1 structures were compared, and 3HNG was selected because it contains the intracellular VEGFR1 kinase domain with an experimentally defined inhibitor-bound ATP pocket, whereas the cited alternatives primarily represent extracellular ligand-binding domains.**

2. In addition, the manuscript lacks a detailed description of the protein preparation procedures employed prior to molecular docking and molecular dynamics simulations. Essential preprocessing steps such as removal or retention of crystallographic water molecules, addition of hydrogen atoms, assignment of appropriate protonation states, correction of missing residues or side chains, and energy minimization protocols were not described. The absence of these methodological details may compromise the reproducibility of the study and potentially introduce structural artifacts capable of affecting docking accuracy and interaction analyses.
To address this limitation, the authors should provide a comprehensive description of the protein preparation workflow, including the software and parameters utilized during structure optimization. Details concerning water molecule treatment, protonation state determination, residue correction, and energy minimization procedures should also be included to enhance methodological transparency and structural reliability.

**Response: Thank you for highlighting this methodological issue. The protein-preparation workflow has been expanded in the revised manuscript. The co-crystallized ligand, chloride ion, and crystallographic water molecules were removed from the receptor used for docking, while the experimental 8ST coordinates were retained separately for redocking validation. Missing residues and heavy atoms were identified and reconstructed using PDBFixer, and the receptor was energy-minimized for 1,000 steps using the GROMOS96 force field in Swiss-PDB Viewer. Hydrogen and charge preparation was also described, including preparation at nominal pH 7.4, retention of polar hydrogens, and assignment of receptor and ligand partial charges for docking.**

3. Furthermore, receptor flexibility was not adequately considered within the computational workflow. The methodology does not specify whether receptor conformational dynamics were incorporated during docking or molecular dynamics simulations. Given that VEGFR1 is a structurally dynamic receptor, the exclusive use of rigid docking approaches may not accurately capture biologically relevant ligand–receptor interactions. Therefore, the authors should clarify whether flexible docking strategies, induced-fit docking, or molecular dynamics simulations were employed to account for receptor flexibility. If such approaches were not implemented, this limitation should be explicitly acknowledged within the discussion section, together with its potential implications for binding prediction accuracy.

**Response: Thank you for this comment. We have clarified receptor flexibility throughout the revised manuscript. Molecular docking was performed using a rigid VEGFR1 receptor with flexible ligand rotatable bonds; induced-fit or flexible-receptor docking was not applied. Receptor flexibility was subsequently incorporated through 500 ns all-atom molecular dynamics simulations, during which the VEGFR1 backbone and side chains were fully flexible and unrestrained. The Discussion now explicitly acknowledges the rigid-receptor approximation of docking and avoids interpreting docking scores as direct measures of binding affinity or receptor stabilization.**

4. Lastly, the structural validation approach appears limited, as the study relied solely on PROCHECK and ERRAT analyses for protein quality assessment. Although these validation tools are widely accepted, they may not provide a sufficiently comprehensive evaluation of the stereochemical and energetic integrity of the receptor structure. To improve confidence in the structural reliability of the selected model, the incorporation of additional validation tools such as Verify3D, ProSA-web, or MolProbity is recommended. The inclusion of complementary validation analyses would provide a broader assessment of structural quality and enhance the overall robustness of the computational methodology.

**Response: Thank you for this valuable suggestion. Structural validation has been expanded beyond PROCHECK and ERRAT by incorporating a complementary MolProbity-based assessment from the wwPDB validation report. The revised manuscript now reports Ramachandran-favored residues and outliers, rotamer quality, Cbeta deviations, bond-length and bond-angle outliers, chiral-volume outliers, twisted peptides, and CaBLAM outliers. These additional metrics provide a broader assessment of the backbone, side-chain, and covalent geometry of the 3HNG structure.**

**Reviewer #2:**

This study uses a broad computational workflow to screen 21 phytochemicals against the VEGFR1 kinase domain from PDB 3HNG. The authors use docking, MOE cross docking, a 500 ns molecular dynamics simulation, MM GBSA and MM PBSA calculations, PCA, DCCM, DFT, and ADMET prediction, with emodin 8 glucoside emerging as the lead compound. There are several positive features here. The protein structure is experimentally determined, the simulation is reasonably long, the data are available through Zenodo, and the authors do acknowledge some of the study’s limitations. The overall sequence of the computational work also makes sense.

Even so, the main claims go well beyond what the results show. Some of the problems are not matters of wording and will require the authors to revisit the analysis and reconsider how the study is framed. For that reason, I recommend Major Revision.

1. My main concern is that the biological mechanism described in the title and abstract does not match the target that was actually modeled. The paper presents the work as an attempt to influence the PlGF and sFlt 1 network. However, the compounds were docked into the intracellular ATP binding site of the VEGFR1 kinase domain. sFlt 1 is a soluble splice form of VEGFR1 that contains the extracellular domains but lacks the intracellular kinase domain. A compound that binds the kinase ATP pocket would not directly change the ability of sFlt 1 to bind PlGF or act as a soluble decoy receptor. The paper would be more accurate if it were presented as a computational investigation of possible inhibition of membrane bound VEGFR1 signaling. The authors should remove the direct sFlt 1 claims unless they can provide a convincing biological explanation for how inhibition of the kinase domain would affect the proposed soluble receptor mechanism.

**Response: Thank you for this important mechanistic clarification. We have substantially reframed the manuscript to distinguish membrane-bound VEGFR1 from soluble sFlt-1. The revised text now states that the modeled ATP-binding pocket belongs to the intracellular kinase domain of membrane-bound VEGFR1 and is absent from sFlt-1; therefore, kinase-pocket binding cannot be interpreted as direct modulation of VEGF/PlGF sequestration by sFlt-1. Direct sFlt-1 mechanistic claims were removed, and the title, abstract, discussion, and conclusion now present Emodin-8-glucoside as an exploratory candidate ligand for the VEGFR1 kinase domain rather than as a demonstrated modulator of the PlGF-sFlt-1 axis.**

2. The disease rationale is also not yet clear. The manuscript does not show that VEGFR1 kinase activity is increased in placenta previa or that inhibiting this pathway would be beneficial in that condition. Much of the discussion comes from studies of preeclampsia and placenta accreta, which are not the same disease as placenta previa. These conditions should not be discussed as though they have the same underlying biology. The authors need to define the clinical problem more carefully and explain why VEGFR1 kinase inhibition is relevant to placenta previa specifically. This is particularly important during pregnancy, since complete loss of VEGFR1 function is associated with severe disruption of vascular development. At this stage, the work is better described as an exploratory computational study than as evidence for a possible treatment.

**Response: Thank you for this comment. The disease rationale has been revised to clearly distinguish placenta previa from placenta accreta spectrum, preeclampsia, and fetal growth restriction. We now explicitly acknowledge that available placenta-previa evidence does not establish increased membrane-bound VEGFR1 kinase activity or demonstrate that VEGFR1 inhibition would be therapeutically beneficial. Accordingly, VEGFR1 is presented as a hypothesis-generating structural target, and the study is consistently framed as an exploratory computational investigation requiring biochemical and placental-model validation rather than evidence for treatment.**

3. There are many inconsistencies in the reported values and units. These need to be checked against the original output files. The docking score is given as negative 9.31 kJ per mol in the abstract and negative 9.31 kcal per mol in Table 3. AutoDock Vina reports its scores in kcal per mol. Similar confusion appears in the MOE and MM GBSA results. The HOMO LUMO gap is listed as 0.13204 Hartree in one place, which is about 3.59 eV, but elsewhere it is reported as 0.13204 eV. The LD50 value changes from 5000 to 3000 mg per kg. The Ramachandran percentages appear to add to more than 100 percent, and different sections disagree about whether any residues fall in disallowed regions. The authors should return to the raw files and report one consistent value for each result.

**Response: Thank you for identifying these inconsistencies. We rechecked the reported values and standardized the corresponding units and interpretations throughout the manuscript. The AutoDock Vina score for Emodin-8-glucoside is now consistently reported as -9.311 kcal/mol, and MOE values are reported separately according to its scoring function. The HOMO-LUMO gap is consistently reported as 0.13204 Hartree, equivalent to approximately 3.59 eV, and the ProTox 3.0 LD50 is reported as 3000 mg/kg. The PROCHECK results now total 100% (85.5% most favored and 14.5% additionally allowed, with no generously allowed or disallowed residues), while the separate MolProbity assessment is reported independently to avoid mixing validation criteria. MM-GBSA and MM-PBSA results have also been standardized to kcal/mol and are now interpreted as method-dependent end-point energy estimates rather than experimentally measured binding free energies.**

4. The docking procedure also needs proper validation. PDB 3HNG contains a crystallographic inhibitor, so the authors should redock that ligand and report the heavy atom RMSD between the experimental pose and the predicted pose. This would provide a basic test of whether the docking setup can reproduce the known binding mode. At present, the results from MOE and Vina do not appear to agree. For example, apigenin 7 O glucoside has a favorable Vina score but a positive MOE value of 22.88. The meaning of that positive value is unclear, and no comparison of the rankings from the two programs is provided. Because of this, the statement that the docking protocol was robust is not supported.

**Response: Thank you for this important comment. We validated the docking protocol by redocking the co-crystallized 8ST ligand into the VEGFR1 ATP-binding pocket. DockRMSD yielded a symmetry-corrected heavy-atom RMSD of 1.465 Å relative to the experimental pose, supporting reasonable pose reproduction. The previously inconsistent positive MOE value was rechecked and corrected, and the complete Vina and MOE rankings were compared using Spearman correlation (rho = 0.553, p = 0.005), indicating moderate agreement rather than complete concordance. We have therefore avoided describing the docking protocol as universally robust and interpret the two scoring methods as complementary.**

5. The reported Vina RMSD value of 0.00 Å for every compound should also be corrected. That value reflects comparison of the top pose with itself. It is not evidence that the ligand is stable or that the docking result is accurate. The Methods should include the docking box coordinates, exhaustiveness, random seed, software version, charge method, and the protonation and tautomer states used for the protein and ligands.

**Response: Thank you for this clarification. The Vina RMSD values of 0.00 Å were removed because they represent self-comparison of the top-ranked pose and do not validate docking accuracy. The Methods now report the docking grid center (x = 7.3 Å, y = 17.8 Å, z = 30.8 Å), a 20 × 20 × 20 Å search box, exhaustiveness of 8, AutoDock Vina Extended v1.2.7 in SAMSON 2026 R1, receptor and ligand charge assignments, nominal pH 7.4, hydrogen treatment, and ligand tautomer handling. No user-defined random seed was specified, and the default stochastic initialization was used. Experimental-pose validation was performed separately by 8ST redocking.**

6. The molecular dynamics section is difficult to interpret because only one simulation of one complex was performed. There is no apo protein simulation, no known inhibitor control, and no independent replicate. Without a comparison group, the authors cannot say that the compound stabilized the protein or reduced its flexibility. Those are relative claims and require something to compare against. The lead compound also scored only slightly better than several other compounds in the docking screen. Differences of this size are usually too small to support a claim that one compound clearly outperformed all others.

**Response: Thank you for this important point. We added a separate 500 ns simulation of the co-crystallized VEGFR1 ligand 8ST under the same conditions as the Emodin-8-glucoside complex, providing a methodologically consistent control for comparative interpretation. We also revised the MD interpretation so that RMSD, RMSF, PCA, DCCM, and related descriptors are not used to claim ligand-induced receptor stabilization or reduced flexibility. In addition, the docking Results now explicitly acknowledge that Emodin-8-glucoside differed only modestly from several other high-ranking compounds and that these score differences do not establish superior experimental affinity.**

7. At minimum, the authors should repeat the simulation using independent initial velocities. A simulation with a known VEGFR1 inhibitor would also make the results much more informative. The full simulation protocol should be reported, including the timestep, treatment of electrostatics, cutoff distances, thermostat, barostat, protein force field, ligand force field, and ligand charge assignment.

**Response: We agree that independent replicate simulations would strengthen assessment of convergence and reproducibility. A 500 ns simulation of the co-crystallized 8ST control was added under identical conditions; however, independent replicate trajectories with different initial velocity seeds were not performed, and this is now explicitly acknowledged as a limitation. The full MD protocol has also been expanded to report the ff19SB protein force field, GAFF2 ligand parameters, AM1-BCC ligand charges, 2 fs timestep, particle-mesh Ewald electrostatics, 10 Å nonbonded cutoff, Langevin thermostat, Monte Carlo barostat, NVT/NPT equilibration, and production conditions.**

8. The MM PBSA and MM GBSA results raise another serious concern. The paper does not clearly state how many frames were analyzed or how far apart they were taken from the trajectory. Based on the reported standard deviations and standard errors, it appears that the calculation may have used only about ten frames. That would not be enough to represent a 500 ns simulation reliably.

**Response: Thank you for identifying this limitation. The revised Methods now explicitly state that MM-GBSA and MM-PBSA calculations used ten snapshots extracted at 50 ns intervals from 50 to 500 ns. We agree that this limited sampling cannot fully represent the conformational ensemble of a 500 ns trajectory. Accordingly, the manuscript now describes these calculations as limited-snapshot, method-dependent end-point energy estimates rather than quantitatively converged binding free energies, and their interpretation has been substantially moderated.**

9. The MM PBSA result of negative 1.63 plus or minus 9.70 kcal per mol is consistent with zero. It also does not agree with the value of negative 36.92 plus or minus 3.7 stated in the abstract. The MM GBSA result of negative 43.11 is very different again. These values cannot all be treated as confirmation of strong binding without an explanation. The authors should analyze a larger number of suitably spaced frames and account for correlation between frames when reporting uncertainty. If entropy was not included, the results should be described as endpoint energy estimates rather than absolute binding free energies.

**Response: Thank you for this important observation. The MM-GBSA/MM-PBSA section was rechecked and substantially revised. The unsupported MM-PBSA value of -36.92 +/- 3.7 kcal/mol was removed, and the Emodin-8-glucoside values are now consistently reported as -43.11 +/- 4.92 kcal/mol for MM-GBSA and -1.63 +/- 9.70 kcal/mol for MM-PBSA. The near-zero MM-PBSA result is explicitly interpreted as highly variable and not as independent confirmation of favorable binding. We also clarified that ten snapshots at 50 ns intervals were analyzed and that configurational entropy was not included. Accordingly, these values are now described as limited-snapshot, method-dependent end-point energy estimates rather than absolute binding free energies. A larger-frame reanalysis was not performed, and this sampling limitation is acknowledged in the revised manuscript.**

10. The hydrogen bond analysis also needs to be checked. An average of about 25 intermolecular hydrogen bonds is unusually high for a small molecule bound to a protein and does not fit well with the eight hydrogen bonds reported from docking. The authors should explain exactly how hydrogen bonds were counted, including the distance and angle criteria. A table showing the occupancy of each ligand protein hydrogen bond would be much more useful than a single average value.
**Response: Thank you for this comment. We rechecked the hydrogen-bond analysis and found that the previously reported average of approximately 25 intermolecular hydrogen bonds was not representative of the trajectory data. The revised analysis now shows predominantly approximately 0.5-2.5 intermolecular hydrogen bonds per frame, with occasional transient peaks near 3.5 and a 50-frame rolling mean generally between approximately 0.7 and 2.2. We respectfully note that the eight hydrogen bonds identified from docking and the hydrogen-bond profile obtained from MD are not directly comparable. Docking describes contacts within a single predicted static pose, whereas MD analysis captures continuous formation and disruption of polar contacts across the trajectory. The purpose of the MD analysis is therefore to characterize time-dependent hydrogen-bond behavior rather than residue-specific occupancy. Because the complete time-resolved hydrogen-bond profile is provided graphically, we have retained this trajectory-level representation rather than introducing an additional residue-occupancy table. The interpretation has also been moderated so that hydrogen-bond counts are not treated as independent evidence of binding affinity, inhibitory potency, or complex stability.**

11. The ADMET discussion does not match the results in the tables. Table 9 shows high predicted probabilities for mutagenicity and immunotoxicity, along with warnings for kidney, heart, respiratory, and general clinical toxicity. The Discussion nevertheless describes the compound as nonmutagenic and suggests that it has a clean toxicity profile. That interpretation is not supported by the results shown.

**Response: Thank you for this comment. The ADMET Results and Discussion have been revised to match the ProTox 3.0 outputs. The manuscript now explicitly reports active predictions for mutagenicity, immunotoxicity, nephrotoxicity, cardiotoxicity, respiratory toxicity, and clinical toxicity, while avoiding descriptions such as "nonmutagenic" or "clean toxicity profile." These computational alerts are now presented as potential liabilities requiring experimental evaluation rather than evidence of safety.**

12. SwissADME also predicts low gastrointestinal absorption, P glycoprotein substrate behavior, and a topological polar surface area of about 174. These findings do not suggest favorable absorption. The BOILED Egg figure also appears to have been interpreted incorrectly. Teratogenicity was not evaluated. Since the paper discusses a possible treatment during pregnancy, the safety predictions need to be reported plainly and with appropriate caution.

**Response: Thank you for highlighting this issue. The SwissADME interpretation has been revised to explicitly acknowledge the high TPSA of 173.98 Å², low predicted gastrointestinal absorption, P-glycoprotein substrate status, and absence of predicted blood-brain barrier permeation. The BOILED-Egg interpretation and caption were also revised to reflect the SwissADME predictions without claiming favorable absorption. In addition, the manuscript now clearly states that teratogenicity, embryotoxicity, reproductive toxicity, fetal toxicity, placental transfer, and other pregnancy-specific safety endpoints were not evaluated; therefore, no conclusion regarding maternal-fetal safety can be drawn from these predictions.**

13. The conclusion should be rewritten to reflect what this type of study can actually establish. Docking and molecular dynamics can help prioritize compounds for further testing, but they do not demonstrate inhibition, selectivity, placental delivery, effectiveness, or safety. The paper should not describe emodin 8 glucoside as a potent, specific, safer, or viable treatment. A more defensible conclusion is that the compound was identified as a computational lead that may be worth testing experimentally.

**Response: Thank you for this important comment. The Conclusion has been completely rewritten and the claims have been substantially moderated. Emodin-8-glucoside is now described as an in-silico-prioritized exploratory candidate rather than a potent, selective, safe, or viable treatment. The revised Conclusion explicitly states that the computational results do not establish VEGFR1 inhibition, therapeutic efficacy, selectivity, or pregnancy safety and that experimental validation is required.**

14. The next steps would include a VEGFR1 kinase assay with a dose response curve and a known inhibitor as a positive control. A VEGFR2 counter screen would be needed to examine selectivity. Studies in appropriate placental cell models would also be necessary before making any claims about possible therapeutic use.

**Response: Thank you for these constructive recommendations. The revised manuscript now proposes a stepwise experimental-validation strategy comprising a biochemical VEGFR1 kinase assay with a dose-response curve and validated inhibitor positive control, a VEGFR2 kinase counter-screen to assess selectivity, and subsequent testing in relevant placental trophoblast and endothelial cell models, including VEGFR1 phosphorylation and downstream functional endpoints.**

15. The references should be reviewed one by one. Some do not support the statements attached to them. An EGFR paper is cited for a claim about VEGFR biology, and the reference used for Gaussian appears to be an unrelated colorectal cancer study. The paper should cite the original or standard references for AutoDock Vina, ff19SB, the ligand force field, MMPBSA.py, and Gaussian.

**Response: The references and their corresponding in-text citations were reviewed to improve alignment between the cited source and the supported statement. Methodological citations were also revised to prioritize appropriate references for the computational tools and protocols, including molecular docking, molecular dynamics force fields, end-point energy calculations, and Gaussian-based DFT analysis.**

16. The manuscript also needs careful language editing. The name of the target is written several different ways, including VEGFR1, VGFR1, and vfr1. Similar inconsistencies appear with PlGF and sFlt 1. Some compound names also change across sections. The description of a steroidal scaffold appears to be incorrect. The reference list contains leftover HTML, and the sentence stating that ciprofloxacin attached favorably to the filter surface is unrelated to this work. The authors should check the rest of the manuscript for similar material.

**Response: Thank you for highlighting these language and consistency issues. The manuscript underwent terminology and language revision to standardize VEGFR1, PlGF, sFlt-1, and compound nomenclature. The incorrect steroidal-scaffold description and the unrelated ciprofloxacin sentence were removed, and the manuscript was reviewed for residual text unrelated to the present study.**

17. The figures should be regenerated at publication quality, and the source data should be provided. The Methods describe a STRING or protein interaction analysis, but no corresponding result is shown. The authors should either include that analysis in the Results or remove it from the Methods.

**Response: Thank you for this comment. The figure set and associated captions were revised for clearer presentation, and the underlying computational data and simulation outputs are available through the publicly accessible Zenodo repository stated in the Data Availability section. The STRING/protein-interaction analysis was removed from the Methods because it was not part of the final reported analysis and therefore had no corresponding Results section.**

18. Overall, the study contains a substantial amount of computational work and identifies a compound that may be worth examining further. However, the manuscript currently has major problems with the proposed mechanism, the disease rationale, the docking validation, the lack of molecular dynamics controls, conflicting energy results, and the interpretation of the toxicity predictions. The central biological issue is especially important because a ligand targeting the intracellular VEGFR1 kinase domain cannot directly support the proposed mechanism involving soluble sFlt 1 and PlGF binding. The paper may become suitable for reconsideration after major reanalysis and reframing, but I cannot recommend acceptance in its current form.

**Response: Thank you for the comprehensive assessment. We have substantially reanalyzed and reframed the manuscript in response to these concerns. The revised study now distinguishes membrane-bound VEGFR1 kinase signaling from soluble sFlt-1 biology, treats placenta previa separately from preeclampsia and PAS, includes crystallographic-ligand redocking and an 8ST control simulation, moderates interpretation of MD and end-point energy results, corrects the ADMET and toxicity interpretation, and presents Emodin-8-glucoside only as an exploratory computational candidate requiring biochemical and placental-model validation. The title, Abstract, Discussion, and Conclusion have been revised accordingly.**

**Reviewer #3:**

Thank you for the opportunity to review this interesting manuscript. The study presents a comprehensive computational investigation of Emodin-8-glucoside as a potential VEGFR1 inhibitor using an integrated workflow including molecular docking, molecular dynamics simulations, MM-GBSA/MM-PBSA analyses, pharmacophore modeling, density functional theory calculations, ADMET prediction, and protein-protein interaction analysis. The computational workflow is systematic, and the public availability of the datasets enhances the transparency and reproducibility of the study. Overall, the work is technically sound and provides a useful computational basis for future experimental investigations.

I have a few suggestions that could further improve the manuscript:

1. Biological rationale: The Introduction would benefit from a stronger discussion explaining why VEGFR1 inhibition is expected to be therapeutically beneficial in placenta previa. Much of the cited evidence relates to preeclampsia, intrauterine growth restrictio

**Response: Thank you for this important suggestion. The Introduction has been substantially revised to clarify the biological rationale and to distinguish placenta previa from preeclampsia, fetal growth restriction, and placenta accreta spectrum. We now explicitly acknowledge that available evidence does not establish increased membrane-bound VEGFR1 kinase activity in placenta previa or demonstrate that VEGFR1 inhibition would be therapeutically beneficial. VEGFR1 is therefore presented as a hypothesis-generating structural target within the angiogenic context of placenta previa, and the study is framed as an exploratory computational investigation rather than evidence of an established therapeutic mechanism. The revised Introduction also distinguishes membrane-bound VEGFR1 kinase signaling from soluble sFlt-1 biology and emphasizes the need for disease-relevant biochemical and placental-model validation.**

**Note to the Editor: The Reviewer #3 report available to us appears to be incomplete and is truncated after the phrase "intrauterine growth restrictio...". Therefore, we have responded to the portion of the comment that was available in the review file. We would be grateful if the complete Reviewer #3 report could be provided so that we can address any remaining comments in full.**
