# Supplementary material for: Structure-based computational prioritization of Emodin-8-glucoside as a candidate ligand for the VEGFR1 kinase domain in the context of placenta previa
Source: PLoS One. 2026 Oct 15;21(10):e0345201. doi: 10.1371/journal.pone.0345201 (PMC13671540; doi:10.1371/journal.pone.0345201)
Supplement: Supplementary file 3 [file pone.0345201.s004.docx]

We thank the Academic Editor and both reviewers for the time and care they have given to this manuscript across two rounds of review. The comments have been consistently constructive and have led to substantive improvements in the framing of the biological target, the validation and reporting of the computational protocols, the handling of uncertainty in the end-point energy estimates, the accuracy of the reference list and the clarity of the text. We are grateful for the opportunity to submit a further revision. Each comment is reproduced below and followed by our response, and the corresponding revisions have been made in the manuscript and marked so that they can be located readily.

**Reviewer #1:**

I have no any other comment because the author has addressed all needed information requested previously.

**Response:** We thank Reviewer 1 for the positive assessment and for the earlier comments, which guided substantial improvements to the analysis and the presentation. No further changes were required in response to this review.

**Reviewer #2:**

I thank the authors for a careful and substantial revision. The three concerns that drove my previous recommendation have been addressed in the analysis itself rather than only in the wording, and the manuscript is much improved. My remaining comments are smaller and can be handled without new calculations. Technical soundness and support for the conclusions.
The reframing of the biological target is now correct. The Introduction (lines 82 to 92) distinguishes membrane bound VEGFR1 from soluble sFlt-1 and states plainly that a ligand occupying the intracellular ATP site would not alter sequestration of VEGF-A or PlGF by sFlt-1. The same distinction is carried through the Methods, Discussion and Conclusion. The disease rationale is also much clearer. The new paragraph at lines 93 to 110 separates placenta previa from placenta accreta spectrum, preeclampsia and fetal growth restriction, and acknowledges that the available evidence does not establish increased VEGFR1 kinase activity in placenta previa. Presenting VEGFR1 as a hypothesis generating structural target is an honest and defensible framing.

The docking protocol is now validated. Redocking of the co-crystallized ligand 8ST gives a symmetry corrected heavy atom RMSD of 1.465 Å, which is a reasonable demonstration that the setup reproduces the known binding mode. The previously unexplained positive MOE value has been corrected, and the two scoring functions are now compared using Spearman rank correlation with the result described as moderate agreement rather than as evidence of robustness. The self comparison RMSD values of 0.00 Å have been removed with an appropriate footnote. The Methods now report the grid center, box dimensions, spacing, exhaustiveness, software version, charge assignment and tautomer handling, which makes the docking reproducible from the text.
The addition of a 500 ns simulation of the VEGFR1 to 8ST complex under identical conditions is the single most valuable change in this revision. It gives the comparative reference that was missing, and the relative claims that previously had no comparator have been removed rather than defended. The statement at line 961 that the analyses cannot establish ligand induced stabilization without apo receptor and replicate simulations is the correct reading of what these data can show.
I also want to acknowledge the handling of the end point energies. The unsupported value has been removed, the remaining values are reported consistently, entropy is stated as excluded, and the calculations are correctly relabeled as end point estimates rather than binding free energies. The authors now report that the co-crystallized control is energetically more favorable than the lead compound under both methods, and they say so directly at lines 962 to 973. Reporting a result that runs against one's own lead compound is good practice and increases my confidence in the revision as a whole.

The ADMET section now matches the underlying tables. The active predictions for mutagenicity, immunotoxicity, nephrotoxicity, cardiotoxicity, respiratory toxicity and clinical toxicity are stated, the earlier language about a clean profile is gone, and the SwissADME findings of high polar surface area, low predicted gastrointestinal absorption and P-glycoprotein substrate status are reported plainly. The explicit statement that teratogenicity, embryotoxicity, reproductive toxicity, fetal toxicity and placental transfer were not evaluated is important given the clinical setting, and I am glad to see it included. The Conclusion has been rewritten appropriately and the proposed experimental path, including a VEGFR1 kinase assay with a positive control, a VEGFR2 counter screen and placental cell models, is sensible.

Statistical analysis: The Spearman rank correlation is the appropriate method for comparing two scoring functions that are not on a common scale, and it is reported with the sample size, the direction convention and the p value. Uncertainties on the energy terms are now reported and, importantly, interpreted. The near zero MM-PBSA value with a large standard deviation is correctly described as uncertain rather than as confirmation of binding. The frame count is now disclosed.

**Response:** We are grateful to Reviewer 2 for a detailed and constructive reading of both the original and the revised manuscript, and for acknowledging the changes made to the framing of the biological target, the disease rationale, the docking validation, the addition of the comparative 500 ns simulation of the VEGFR1 to 8ST complex, the reporting of the end-point energies, the ADMET section and the statistical treatment. The remaining points are addressed individually below.

One point remains open. Correlation between frames is still not addressed in the reported uncertainty. In practice the 50 ns spacing makes these snapshots close to independent, so I do not think the standard deviations are seriously optimistic, but ten snapshots is a small sample and no convergence or block analysis is shown. I would also note that the numerical differences between the lead and control energies, 6.41 and 8.35 kcal per mol, are presented without any assessment of whether they are distinguishable given the spread. Since these differences do not favor the lead compound, this omission does not inflate any claim, but a brief statement would be helpful.

**Response:** We accept this point and have addressed it explicitly rather than by implication. The Methods now state that the ten snapshots used for the end-point calculations were taken at 50 ns intervals between 50 and 500 ns, that correlation between snapshots at this spacing is expected to be limited, and that no block averaging or formal convergence assessment was performed; the reported means, standard deviations and standard errors are accordingly presented as descriptive of the ten sampled snapshots rather than as converged estimates of the underlying distributions. The Results now describe the differences of 6.41 and 8.35 kcal/mol between the lead compound and the co-crystallized control as not formally distinguishable given the standard deviations and the small number of snapshots, and the limitations paragraph repeats that a single 500 ns trajectory per complex was run, without replicates, and that no block averaging or convergence analysis was carried out. Consistent with the reviewer's observation that these differences do not favor the lead compound, no inference is drawn from their magnitude.

Remaining items to address

Hydrogen bond analysis. This is the one item I would ask to be resolved before acceptance. The reported average has changed from approximately 25 intermolecular hydrogen bonds to approximately 0.5 to 2.5 per frame. The new values are physically reasonable and I accept them. However, the distance and angle criteria I asked for are still not given. The Methods at line 378 list protein to ligand hydrogen bonds among the trajectory analyses but define nothing, and no software is named for the trajectory analysis. The caption to Figure 8A refers to the stated hydrogen bond analysis protocol, but no protocol is stated anywhere in the manuscript. Please add the donor to acceptor distance cutoff, the angle criterion and the analysis program. A sentence explaining what produced the earlier value would also be appropriate, since a tenfold change in a reported quantity should be accounted for. I accept the authors' reasoning for presenting the trajectory level profile rather than a residue occupancy table.

**Response:** We apologize for leaving this unspecified and have now stated the protocol in full. The Methods report that all trajectory analyses, including the receptor to ligand hydrogen bond analysis, were performed with the CPPTRAJ module of AmberTools25, cited as reference 52, and that a hydrogen bond was counted when the donor to acceptor heavy-atom distance was at or below 3.0 Å and the donor to hydrogen to acceptor angle was at or above 135°, with the search restricted to direct intermolecular receptor to ligand donor and acceptor pairs so that intramolecular hydrogen bonds and water-bridged contacts are excluded. A sentence has also been added accounting for the change in the reported average, which arose because the earlier count was not restricted to direct intermolecular pairs and consequently aggregated intramolecular and water-mediated contacts; the recomputed values obtained under the criteria above supersede it. The captions to Figures 8 and 11 now refer to the hydrogen bond distance and angle criteria stated in the Methods rather than to an unstated protocol. We thank the reviewer for accepting the trajectory-level profile in place of a residue occupancy table.

Replication and sampling. I accept the authors' decision not to run replicate trajectories, given that the claims have been narrowed accordingly and the limitation is clearly stated at lines 1138 to 1145. If it is convenient, recomputing MM-GBSA and MM-PBSA over the existing trajectories at closer frame spacing would require no additional simulation time and would strengthen this section, but I do not regard it as necessary for acceptance.

**Response:** We thank the reviewer for accepting this decision. The claims remain narrowed accordingly, and the limitations paragraph continues to state that a single 500 ns trajectory was run per complex, that no replicate trajectories were performed, and that the end-point estimates rest on ten snapshots without block averaging or a formal convergence assessment. We have not recomputed the end-point energies at closer frame spacing for this revision. So that this can be pursued directly by any interested reader, the per-snapshot energy values behind the reported means, standard deviations and standard errors are included in the deposited archive.

References. My previous comment on citation accuracy has only been partly addressed, and one of the specific problems has moved rather than been fixed. a. Reference 10 is a paper on the epidermal growth factor receptor in pregnancy and preeclampsia. It is still cited at line 102 in support of a statement about FLT1 and sFlt-1 expression. Reference 9 supports that statement; reference 10 does not. b. At line 106, reference 53 is a study of phytochemicals in spinocerebellar ataxia type 3 and is cited in support of placenta previa tissue studies. Reference 11 is the appropriate source. The bracket formatting there, "(53) (11)", is also malformed. c. The primary AutoDock Vina reference is still absent. Reference 39 is a tutorial and reference 40 is a colorectal cancer study by the same authors. The original description of the program should be cited. Similarly, reference 48 is a force field benchmarking study rather than the original description of the ligand force field. The references for Gaussian, MMPBSA.py and ff19SB are now correct. d. Reference 3 still contains a leftover HTML entity.

**Response:** We are grateful for the continued attention to the reference list. The incorrect reference has been removed from the statement on FLT1 and sFlt-1 expression, which now cites references 8 and 9. The malformed bracket in the sentence on "pernicious" placenta previa has been replaced by a single, correct citation to the biomarker source, reference 1. The primary description of AutoDock Vina by Trott and Olson, published in 2010, has been added as reference 40 alongside the earlier references, so that references 38 to 40 are now cited together for the docking program. The original description of the general ligand force field by Wang and co-workers, published in 2004, is now cited as reference 48 in place of the force field benchmarking study, and the trajectory analysis program is cited as reference 52, alongside the existing citations for the semi-empirical charge model and the end-point energy implementation at references 47 and 53. The stray character in reference 3 has been removed and the reference list has been regenerated. Each of these citation changes is marked in the revised file so that it can be located readily.

Language and presentation. Several duplications appear to have been introduced during revision. Because PLOS ONE does not copyedit accepted manuscripts, these should be corrected now.
Abstract, lines 40 to 41: a clause is repeated within one sentence, reading "End-point energy calculations were method dependent, end-point energy estimates were method dependent". This text also appears in the abstract field of the submission system and should be corrected in both places.

Discussion, lines 933 to 934: the sentence beginning "The interfacial analyses revealed different interaction patterns" appears twice consecutively.

Methods, lines 297 to 307: the sentence describing placement of the docking grid over Pocket 1 appears twice.

Line 271: "uploaded in verse to the PDB format" appears to be a garbled phrase.
Funding statement, line 1182: refers to "the completion of this review". This is a research article.

Figure 4 caption gives the Vina score as -9.31 while the text and Table 2 give -9.311. Table 2 gives the MOE score as -8.32291 while the text gives -8.3229.

The short title still reads "In Silico Identification of Emodin-8-glucoside as a VEGFR1 Drug Candidate", which is stronger than the revised main title and the revised conclusions. "Candidate ligand" would be consistent.

Because three separate duplications were found, I suggest a full read through for any others.

**Response:** We apologize for these duplications, which were introduced while consolidating text during the previous revision, and all have been removed. The repeated clause in the Abstract has been deleted, and the corrected abstract will be entered in the submission system so that the two versions agree. The duplicated sentence in the Discussion concerning the interfacial interaction patterns and the duplicated sentence in the Methods describing placement of the docking grid over Pocket 1 have each been reduced to a single occurrence. The garbled phrase in the Methods has been rewritten and now reads that the curated VEGFR1 model was uploaded in PDB format. The funding statement has been corrected and now refers to a research article. The docking scores are reported consistently, with the Vina score for Emodin-8-glucoside given as -9.311 kcal/mol in the Results text and in the Figure 4 caption and the MOE GBVI/WSA score given as -8.3229 in the Results text and in Table 2; Figure 4 is now also cited in the Results text. The short title flagged by the reviewer does not appear in the manuscript file and exists only in the submission system; it has been changed there to read candidate ligand rather than drug candidate, so that it is consistent with the revised main title and the revised conclusions. Following the reviewer's suggestion, we have read the whole manuscript through once more for any further duplications.

Data availability. The Zenodo deposit is appropriate and no restrictions are claimed. Please confirm that the archive contains the per snapshot energy values behind the reported means and standard deviations and the raw docking output files, and not only summary tables, since the data points behind means and variance measures fall within the journal's policy.

**Response:** We confirm that the public Zenodo record contains the per-snapshot MM-GBSA and MM-PBSA energy values underlying every reported mean, standard deviation and standard error, together with the raw docking output files, so that the individual data points behind the reported means and variance measures are available rather than summary tables alone. The record, which is available at <https://doi.org/10.5281/zenodo.17528796>, also holds the ligand structure files in SDF and PDB format, the receptor structure files, the active-site prediction output and the density functional theory output, and the manuscript's Data Availability statement lists these datasets together with the deposited video of the molecular dynamics trajectories. One limitation should be stated openly rather than left for the reviewer to infer. Each simulated complex was run for 500 ns, and the raw trajectory files therefore amount to several gigabytes. What the authors currently have deposited is the video record of those trajectories; the full raw trajectory files exceeded the upload capacity available at the time of resubmission, a temporary internet connectivity limitation at the authors' location, and they are consequently not yet in the repository. We will add the complete trajectory files to the same Zenodo record once upload becomes possible, and in the meantime we can supply them directly to the editorial office or to the reviewer on request.

Figures. The figures are supplied as a Word file. Please ensure that final versions meet the journal's format and resolution requirements.

**Response:** We confirm that the final figures will be supplied as individual files meeting the journal's format and resolution requirements, rather than embedded in a Word document.

Recommendation

The central problems I raised previously have been resolved, and the conclusions are now appropriately matched to what a computational study of this kind can support. The remaining items are matters of documentation, citation accuracy and language, none of which require new computation or are likely to alter any finding. I recommend Minor Revision, with the hydrogen bond methods specification treated as a condition of acceptance.

**Response:** We thank Reviewer 2 for the careful evaluation across both rounds and for the recommendation. The hydrogen bond methods specification identified as a condition of acceptance has been implemented in full, with the distance and angle criteria, the restriction to direct intermolecular receptor to ligand pairs and the analysis program all stated in the Methods, and the remaining items of documentation, citation accuracy and language have been addressed as described above. No finding has changed as a result of these revisions.
