# Supplementary material for: Structure-based computational prioritization of Emodin-8-glucoside as a candidate ligand for the VEGFR1 kinase domain in the context of placenta previa
Source: PLoS One. 2026 Oct 15;21(10):e0345201. doi: 10.1371/journal.pone.0345201 (PMC13671540; doi:10.1371/journal.pone.0345201)
Supplement: Peer review history

# Peer review: Decision Letter 0

**DOI:** 10.1371/journal.pone.0345201.r001

**Authors:** Yusuf Ayipo

23 Jul 2026

Dear Dr. Ali,

Thank you for submitting your manuscript to PLOS ONE. After careful consideration, we feel that it has merit but does not fully meet PLOS ONE’s publication criteria as it currently stands. Therefore, we invite you to submit a revised version of the manuscript that addresses the points raised during the review process.

==============================

**ACADEMIC EDITOR:** The submission reflects scientific relevance as adjudged by the reviewers. However, some fundamental issues limit its quality for publication in the current form. For instance, the absence of adequate discussion of the results with appropriate references has watered down the reproducibility of the methodologies, results and the overall chance for publication of the current submission. Additionally, the authors need to justify the significance of the study, relate it to the literature and identify the gap in the existing knowledge that this study aims to address, and ensure an adequate validation of all analyses reported. Again, what are the limitations of this study, and how can the authors recommend future research on the study? Moreover, some other major concerns have been raised by the reviewers affecting pivotal sections of the study. Kindly pay close attention to these and address them critically before resubmission.

==============================

Please submit your revised manuscript by Sep 05 2026 11:59PM. If you will need more time than this to complete your revisions, please reply to this message or contact the journal office at plosone@plos.org. When you're ready to submit your revision, log on to https://www.editorialmanager.com/pone/ and select the 'Submissions Needing Revision' folder to locate your manuscript file.

- A letter that responds to each point raised by the academic editor and reviewer(s). You should upload this letter as a separate file labeled 'Response to Reviewers'.
- A marked-up copy of your manuscript that highlights changes made to the original version. You should upload this as a separate file labeled 'Revised Manuscript with Track Changes'.
- An unmarked version of your revised paper without tracked changes. You should upload this as a separate file labeled 'Manuscript'.

If you would like to make changes to your financial disclosure, please include your updated statement in your cover letter. Guidelines for resubmitting your figure files are available below the reviewer comments at the end of this letter.

If applicable, we recommend that you deposit your laboratory protocols in protocols.io to enhance the reproducibility of your results. Protocols.io assigns your protocol its own identifier (DOI) so that it can be cited independently in the future. For instructions see: https://journals.plos.org/plosone/s/submission-guidelines#loc-laboratory-protocols. Additionally, PLOS ONE offers an option for publishing peer-reviewed Lab Protocol articles, which describe protocols hosted on protocols.io. Read more information on sharing protocols at https://plos.org/protocols?utm_medium=editorial-email&utm_source=authorletters&utm_campaign=protocols.

As the corresponding author, your ORCID iD is verified in the submission system and will appear in the published article. PLOS supports the use of ORCID, and we encourage all coauthors to register for an ORCID iD and use it as well. Please encourage your coauthors to verify their ORCID iD within the submission system before final acceptance, as unverified ORCID iDs will not appear in the published article. *Only*  the individual author can complete the verification step; PLOS staff *cannot*  verify ORCID iDs on behalf of authors.

We look forward to receiving your revised manuscript.

Kind regards,

Yusuf Oloruntoyin Ayipo, Ph.D

Academic Editor

PLOS One

Journal requirements:

When submitting your revision, we need you to address these additional requirements.

1. Please ensure that your manuscript meets PLOS ONE's style requirements, including those for file naming. The PLOS ONE style templates can be found at

https://journals.plos.org/plosone/s/file?id=wjVg/PLOSOne_formatting_sample_main_body.pdf and

https://journals.plos.org/plosone/s/file?id=ba62/PLOSOne_formatting_sample_title_authors_affiliations.pdf

2. We note that you have declared the use of generative AI in the preparation of your manuscript. Please note that contributions by artificial intelligence (AI) tools and technologies to a study or to an article’s contents must be clearly reported in a dedicated section of the Methods, or in the Acknowledgements section for article types lacking a Methods section. This section should include the name(s) of any tools used, a description of how the authors used the tool(s) and evaluated the validity of the tool’s outputs, and a clear statement of which aspects of the study, article contents, data, or supporting files were affected/generated by AI tool usage (https://journals.plos.org/plosone/s/ethical-publishing-practice#loc-artificial-intelligence-tools-and-technologies).

Please therefore revise your manuscript to provide all necessary information to meet these requirements. We appreciate your attention to these queries and look forward to your response.

If the reviewer comments include a recommendation to cite specific previously published works, please review and evaluate these publications to determine whether they are relevant and should be cited. There is no requirement to cite these works unless the editor has indicated otherwise.

Additional Editor Comments:

The submission reflects scientific relevance as adjudged by the reviewers. However, some fundamental issues limit its quality for publication in the current form. For instance, the absence of adequate discussion of the results with appropriate references has watered down the reproducibility of the methodologies, results and the overall chance for publication of the current submission. Additionally, the authors need to justify the significance of the study, relate it to the literature and identify the gap in the existing knowledge that this study aims to address, and ensure an adequate validation of all analyses reported. Again, what are the limitations of this study, and how can the authors recommend future research on the study? Moreover, some other major concerns have been raised by the reviewers affecting pivotal sections of the study. Kindly pay close attention to these and address them critically before resubmission.

[Note: HTML markup is below. Please do not edit.]

Reviewers' comments:

Reviewer's Responses to Questions

**Comments to the Author**

1. Is the manuscript technically sound, and do the data support the conclusions?

Reviewer #1: Yes

Reviewer #2: No

Reviewer #3: Yes

**********

2. Has the statistical analysis been performed appropriately and rigorously?

Reviewer #1: N/A

Reviewer #2: No

Reviewer #3: Yes

**********

3. Have the authors made all data underlying the findings in their manuscript fully available?

The PLOS Data policy

Reviewer #1: Yes

Reviewer #2: Yes

Reviewer #3: Yes

**********

4. Is the manuscript presented in an intelligible fashion and written in standard English?

Reviewer #1: Yes

Reviewer #2: No

Reviewer #3: Yes

**********

Reviewer #1: The selection of the VEGFR1 crystal structure (PDB ID: 3HNG) was attributed to its high-resolution crystallographic data; however, the manuscript does not report the exact resolution value of the structure. Furthermore, the authors did not provide sufficient justification regarding the biological relevance of the selected conformation, including whether 3HNG represents the active or inactive state of VEGFR1, or whether the structure contains co-crystallized ligands, mutations, or missing residues that could influence docking accuracy and ligand-binding predictions. Considering the availability of multiple VEGFR1 structures within the Protein Data Bank, a more comprehensive rationale for the selection of 3HNG is warranted.

To strengthen this section, the authors should explicitly report the crystallographic resolution of the selected structure and provide a comparative justification against other available VEGFR1 crystal structures. In addition, clarification regarding the conformational state and structural relevance of 3HNG for ligand-binding investigations would improve the scientific validity of the docking protocol.

In addition, the manuscript lacks a detailed description of the protein preparation procedures employed prior to molecular docking and molecular dynamics simulations. Essential preprocessing steps such as removal or retention of crystallographic water molecules, addition of hydrogen atoms, assignment of appropriate protonation states, correction of missing residues or side chains, and energy minimization protocols were not described. The absence of these methodological details may compromise the reproducibility of the study and potentially introduce structural artifacts capable of affecting docking accuracy and interaction analyses.

To address this limitation, the authors should provide a comprehensive description of the protein preparation workflow, including the software and parameters utilized during structure optimization. Details concerning water molecule treatment, protonation state determination, residue correction, and energy minimization procedures should also be included to enhance methodological transparency and structural reliability.

Furthermore, receptor flexibility was not adequately considered within the computational workflow. The methodology does not specify whether receptor conformational dynamics were incorporated during docking or molecular dynamics simulations. Given that VEGFR1 is a structurally dynamic receptor, the exclusive use of rigid docking approaches may not accurately capture biologically relevant ligand–receptor interactions.

Therefore, the authors should clarify whether flexible docking strategies, induced-fit docking, or molecular dynamics simulations were employed to account for receptor flexibility. If such approaches were not implemented, this limitation should be explicitly acknowledged within the discussion section, together with its potential implications for binding prediction accuracy.

Lastly, the structural validation approach appears limited, as the study relied solely on PROCHECK and ERRAT analyses for protein quality assessment. Although these validation tools are widely accepted, they may not provide a sufficiently comprehensive evaluation of the stereochemical and energetic integrity of the receptor structure.

To improve confidence in the structural reliability of the selected model, the incorporation of additional validation tools such as Verify3D, ProSA-web, or MolProbity is recommended. The inclusion of complementary validation analyses would provide a broader assessment of structural quality and enhance the overall robustness of the computational methodology.

Reviewer #2: This study uses a broad computational workflow to screen 21 phytochemicals against the VEGFR1 kinase domain from PDB 3HNG. The authors use docking, MOE cross docking, a 500 ns molecular dynamics simulation, MM GBSA and MM PBSA calculations, PCA, DCCM, DFT, and ADMET prediction, with emodin 8 glucoside emerging as the lead compound. There are several positive features here. The protein structure is experimentally determined, the simulation is reasonably long, the data are available through Zenodo, and the authors do acknowledge some of the study’s limitations. The overall sequence of the computational work also makes sense.

Even so, the main claims go well beyond what the results show. Some of the problems are not matters of wording and will require the authors to revisit the analysis and reconsider how the study is framed. For that reason, I recommend Major Revision.

My main concern is that the biological mechanism described in the title and abstract does not match the target that was actually modeled. The paper presents the work as an attempt to influence the PlGF and sFlt 1 network. However, the compounds were docked into the intracellular ATP binding site of the VEGFR1 kinase domain. sFlt 1 is a soluble splice form of VEGFR1 that contains the extracellular domains but lacks the intracellular kinase domain. A compound that binds the kinase ATP pocket would not directly change the ability of sFlt 1 to bind PlGF or act as a soluble decoy receptor. The paper would be more accurate if it were presented as a computational investigation of possible inhibition of membrane bound VEGFR1 signaling. The authors should remove the direct sFlt 1 claims unless they can provide a convincing biological explanation for how inhibition of the kinase domain would affect the proposed soluble receptor mechanism.

The disease rationale is also not yet clear. The manuscript does not show that VEGFR1 kinase activity is increased in placenta previa or that inhibiting this pathway would be beneficial in that condition. Much of the discussion comes from studies of preeclampsia and placenta accreta, which are not the same disease as placenta previa. These conditions should not be discussed as though they have the same underlying biology. The authors need to define the clinical problem more carefully and explain why VEGFR1 kinase inhibition is relevant to placenta previa specifically. This is particularly important during pregnancy, since complete loss of VEGFR1 function is associated with severe disruption of vascular development. At this stage, the work is better described as an exploratory computational study than as evidence for a possible treatment.

There are many inconsistencies in the reported values and units. These need to be checked against the original output files. The docking score is given as negative 9.31 kJ per mol in the abstract and negative 9.31 kcal per mol in Table 3. AutoDock Vina reports its scores in kcal per mol. Similar confusion appears in the MOE and MM GBSA results. The HOMO LUMO gap is listed as 0.13204 Hartree in one place, which is about 3.59 eV, but elsewhere it is reported as 0.13204 eV. The LD50 value changes from 5000 to 3000 mg per kg. The Ramachandran percentages appear to add to more than 100 percent, and different sections disagree about whether any residues fall in disallowed regions. The authors should return to the raw files and report one consistent value for each result.

The docking procedure also needs proper validation. PDB 3HNG contains a crystallographic inhibitor, so the authors should redock that ligand and report the heavy atom RMSD between the experimental pose and the predicted pose. This would provide a basic test of whether the docking setup can reproduce the known binding mode. At present, the results from MOE and Vina do not appear to agree. For example, apigenin 7 O glucoside has a favorable Vina score but a positive MOE value of 22.88. The meaning of that positive value is unclear, and no comparison of the rankings from the two programs is provided. Because of this, the statement that the docking protocol was robust is not supported.

The reported Vina RMSD value of 0.00 Å for every compound should also be corrected. That value reflects comparison of the top pose with itself. It is not evidence that the ligand is stable or that the docking result is accurate. The Methods should include the docking box coordinates, exhaustiveness, random seed, software version, charge method, and the protonation and tautomer states used for the protein and ligands.

The molecular dynamics section is difficult to interpret because only one simulation of one complex was performed. There is no apo protein simulation, no known inhibitor control, and no independent replicate. Without a comparison group, the authors cannot say that the compound stabilized the protein or reduced its flexibility. Those are relative claims and require something to compare against. The lead compound also scored only slightly better than several other compounds in the docking screen. Differences of this size are usually too small to support a claim that one compound clearly outperformed all others.

At minimum, the authors should repeat the simulation using independent initial velocities. A simulation with a known VEGFR1 inhibitor would also make the results much more informative. The full simulation protocol should be reported, including the timestep, treatment of electrostatics, cutoff distances, thermostat, barostat, protein force field, ligand force field, and ligand charge assignment.

The MM PBSA and MM GBSA results raise another serious concern. The paper does not clearly state how many frames were analyzed or how far apart they were taken from the trajectory. Based on the reported standard deviations and standard errors, it appears that the calculation may have used only about ten frames. That would not be enough to represent a 500 ns simulation reliably.

The MM PBSA result of negative 1.63 plus or minus 9.70 kcal per mol is consistent with zero. It also does not agree with the value of negative 36.92 plus or minus 3.7 stated in the abstract. The MM GBSA result of negative 43.11 is very different again. These values cannot all be treated as confirmation of strong binding without an explanation. The authors should analyze a larger number of suitably spaced frames and account for correlation between frames when reporting uncertainty. If entropy was not included, the results should be described as endpoint energy estimates rather than absolute binding free energies.

The hydrogen bond analysis also needs to be checked. An average of about 25 intermolecular hydrogen bonds is unusually high for a small molecule bound to a protein and does not fit well with the eight hydrogen bonds reported from docking. The authors should explain exactly how hydrogen bonds were counted, including the distance and angle criteria. A table showing the occupancy of each ligand protein hydrogen bond would be much more useful than a single average value.

The ADMET discussion does not match the results in the tables. Table 9 shows high predicted probabilities for mutagenicity and immunotoxicity, along with warnings for kidney, heart, respiratory, and general clinical toxicity. The Discussion nevertheless describes the compound as nonmutagenic and suggests that it has a clean toxicity profile. That interpretation is not supported by the results shown.

SwissADME also predicts low gastrointestinal absorption, P glycoprotein substrate behavior, and a topological polar surface area of about 174. These findings do not suggest favorable absorption. The BOILED Egg figure also appears to have been interpreted incorrectly. Teratogenicity was not evaluated. Since the paper discusses a possible treatment during pregnancy, the safety predictions need to be reported plainly and with appropriate caution.

The conclusion should be rewritten to reflect what this type of study can actually establish. Docking and molecular dynamics can help prioritize compounds for further testing, but they do not demonstrate inhibition, selectivity, placental delivery, effectiveness, or safety. The paper should not describe emodin 8 glucoside as a potent, specific, safer, or viable treatment. A more defensible conclusion is that the compound was identified as a computational lead that may be worth testing experimentally.

The next steps would include a VEGFR1 kinase assay with a dose response curve and a known inhibitor as a positive control. A VEGFR2 counter screen would be needed to examine selectivity. Studies in appropriate placental cell models would also be necessary before making any claims about possible therapeutic use.

The references should be reviewed one by one. Some do not support the statements attached to them. An EGFR paper is cited for a claim about VEGFR biology, and the reference used for Gaussian appears to be an unrelated colorectal cancer study. The paper should cite the original or standard references for AutoDock Vina, ff19SB, the ligand force field, MMPBSA.py, and Gaussian.

The manuscript also needs careful language editing. The name of the target is written several different ways, including VEGFR1, VGFR1, and vfr1. Similar inconsistencies appear with PlGF and sFlt 1. Some compound names also change across sections. The description of a steroidal scaffold appears to be incorrect. The reference list contains leftover HTML, and the sentence stating that ciprofloxacin attached favorably to the filter surface is unrelated to this work. The authors should check the rest of the manuscript for similar material.

The figures should be regenerated at publication quality, and the source data should be provided. The Methods describe a STRING or protein interaction analysis, but no corresponding result is shown. The authors should either include that analysis in the Results or remove it from the Methods.

Overall, the study contains a substantial amount of computational work and identifies a compound that may be worth examining further. However, the manuscript currently has major problems with the proposed mechanism, the disease rationale, the docking validation, the lack of molecular dynamics controls, conflicting energy results, and the interpretation of the toxicity predictions. The central biological issue is especially important because a ligand targeting the intracellular VEGFR1 kinase domain cannot directly support the proposed mechanism involving soluble sFlt 1 and PlGF binding. The paper may become suitable for reconsideration after major reanalysis and reframing, but I cannot recommend acceptance in its current form.

Reviewer #3: Thank you for the opportunity to review this interesting manuscript. The study presents a comprehensive computational investigation of Emodin-8-glucoside as a potential VEGFR1 inhibitor using an integrated workflow including molecular docking, molecular dynamics simulations, MM-GBSA/MM-PBSA analyses, pharmacophore modeling, density functional theory calculations, ADMET prediction, and protein-protein interaction analysis. The computational workflow is systematic, and the public availability of the datasets enhances the transparency and reproducibility of the study. Overall, the work is technically sound and provides a useful computational basis for future experimental investigations.

I have a few suggestions that could further improve the manuscript:

1. Biological rationale: The Introduction would benefit from a stronger discussion explaining why VEGFR1 inhibition is expected to be therapeutically beneficial in placenta previa. Much of the cited evidence relates to preeclampsia, intrauterine growth restrictio

**********

what does this mean?). If published, this will include your full peer review and any attached files.

If you choose “no”, your identity will remain anonymous but your review may still be made public.

**Do you want your identity to be public for this peer review?**  For information about this choice, including consent withdrawal, please see our Privacy Policy

Reviewer #1: No

Reviewer #2: No

Reviewer #3: No

**********

[NOTE: If reviewer comments were submitted as an attachment file, they will be attached to this email and accessible via the submission site. Please log into your account, locate the manuscript record, and check for the action link "View Attachments". If this link does not appear, there are no attachment files.]

To ensure your figures meet our technical requirements, please review our figure guidelines: https://journals.plos.org/plosone/s/figures

You may also use PLOS’s free figure tool, NAAS, to help you prepare publication quality figures: https://journals.plos.org/plosone/s/figures#loc-tools-for-figure-preparation.

NAAS will assess whether your figures meet our technical requirements by comparing each figure against our figure specifications.

# Author comment: Author response to Decision Letter 1

**DOI:** 10.1371/journal.pone.0345201.r002

18 Aug 2026

Dear Editor and Reviewers,

We sincerely thank the Editor and all Reviewers for their careful evaluation of our manuscript and for providing constructive and insightful comments. Their suggestions have helped us substantially improve the scientific rigor, clarity, methodological transparency, and interpretation of the study. We have carefully considered each comment and revised the manuscript accordingly, including the biological rationale, structural selection and validation, docking methodology, molecular dynamics analysis, end-point energy interpretation, ADMET assessment, terminology, and overall framing of the conclusions. All changes have been incorporated into the revised manuscript, and detailed point-by-point responses are provided below. We greatly appreciate the time and effort invested by the Editor and Reviewers in helping us strengthen the manuscript.

Reviewer #1:

1. The selection of the VEGFR1 crystal structure (PDB ID: 3HNG) was attributed to its high-resolution crystallographic data; however, the manuscript does not report the exact resolution value of the structure. Furthermore, the authors did not provide sufficient justification regarding the biological relevance of the selected conformation, including whether 3HNG represents the active or inactive state of VEGFR1, or whether the structure contains co-crystallized ligands, mutations, or missing residues that could influence docking accuracy and ligand-binding predictions. Considering the availability of multiple VEGFR1 structures within the Protein Data Bank, a more comprehensive rationale for the selection of 3HNG is warranted. To strengthen this section, the authors should explicitly report the crystallographic resolution of the selected structure and provide a comparative justification against other available VEGFR1 crystal structures. In addition, clarification regarding the conformational state and structural relevance of 3HNG for ligand-binding investigations would improve the scientific validity of the docking protocol.

Response: Thank you for this important comment. We have expanded the structural-selection section to provide a detailed rationale for using PDB ID 3HNG. The revised manuscript now reports its X-ray resolution of 2.70 Å, identifies the co-crystallized inhibitor 8ST, confirms the absence of annotated mutations in the aligned VEGFR1 sequence, and specifies the modeled and unresolved residues. We also clarified that 3HNG adopts a DFG-out/alphaC-helix-in inactive-like kinase conformation. Representative alternative VEGFR1 structures were compared, and 3HNG was selected because it contains the intracellular VEGFR1 kinase domain with an experimentally defined inhibitor-bound ATP pocket, whereas the cited alternatives primarily represent extracellular ligand-binding domains.

2. In addition, the manuscript lacks a detailed description of the protein preparation procedures employed prior to molecular docking and molecular dynamics simulations. Essential preprocessing steps such as removal or retention of crystallographic water molecules, addition of hydrogen atoms, assignment of appropriate protonation states, correction of missing residues or side chains, and energy minimization protocols were not described. The absence of these methodological details may compromise the reproducibility of the study and potentially introduce structural artifacts capable of affecting docking accuracy and interaction analyses.

To address this limitation, the authors should provide a comprehensive description of the protein preparation workflow, including the software and parameters utilized during structure optimization. Details concerning water molecule treatment, protonation state determination, residue correction, and energy minimization procedures should also be included to enhance methodological transparency and structural reliability.

Response: Thank you for highlighting this methodological issue. The protein-preparation workflow has been expanded in the revised manuscript. The co-crystallized ligand, chloride ion, and crystallographic water molecules were removed from the receptor used for docking, while the experimental 8ST coordinates were retained separately for redocking validation. Missing residues and heavy atoms were identified and reconstructed using PDBFixer, and the receptor was energy-minimized for 1,000 steps using the GROMOS96 force field in Swiss-PDB Viewer. Hydrogen and charge preparation was also described, including preparation at nominal pH 7.4, retention of polar hydrogens, and assignment of receptor and ligand partial charges for docking.

3. Furthermore, receptor flexibility was not adequately considered within the computational workflow. The methodology does not specify whether receptor conformational dynamics were incorporated during docking or molecular dynamics simulations. Given that VEGFR1 is a structurally dynamic receptor, the exclusive use of rigid docking approaches may not accurately capture biologically relevant ligand–receptor interactions. Therefore, the authors should clarify whether flexible docking strategies, induced-fit docking, or molecular dynamics simulations were employed to account for receptor flexibility. If such approaches were not implemented, this limitation should be explicitly acknowledged within the discussion section, together with its potential implications for binding prediction accuracy.

Response: Thank you for this comment. We have clarified receptor flexibility throughout the revised manuscript. Molecular docking was performed using a rigid VEGFR1 receptor with flexible ligand rotatable bonds; induced-fit or flexible-receptor docking was not applied. Receptor flexibility was subsequently incorporated through 500 ns all-atom molecular dynamics simulations, during which the VEGFR1 backbone and side chains were fully flexible and unrestrained. The Discussion now explicitly acknowledges the rigid-receptor approximation of docking and avoids interpreting docking scores as direct measures of binding affinity or receptor stabilization.

4. Lastly, the structural validation approach appears limited, as the study relied solely on PROCHECK and ERRAT analyses for protein quality assessment. Although these validation tools are widely accepted, they may not provide a sufficiently comprehensive evaluation of the stereochemical and energetic integrity of the receptor structure. To improve confidence in the structural reliability of the selected model, the incorporation of additional validation tools such as Verify3D, ProSA-web, or MolProbity is recommended. The inclusion of complementary validation analyses would provide a broader assessment of structural quality and enhance the overall robustness of the computational methodology.

Response: Thank you for this valuable suggestion. Structural validation has been expanded beyond PROCHECK and ERRAT by incorporating a complementary MolProbity-based assessment from the wwPDB validation report. The revised manuscript now reports Ramachandran-favored residues and outliers, rotamer quality, Cbeta deviations, bond-length and bond-angle outliers, chiral-volume outliers, twisted peptides, and CaBLAM outliers. These additional metrics provide a broader assessment of the backbone, side-chain, and covalent geometry of the 3HNG structure.

Reviewer #2:

This study uses a broad computational workflow to screen 21 phytochemicals against the VEGFR1 kinase domain from PDB 3HNG. The authors use docking, MOE cross docking, a 500 ns molecular dynamics simulation, MM GBSA and MM PBSA calculations, PCA, DCCM, DFT, and ADMET prediction, with emodin 8 glucoside emerging as the lead compound. There are several positive features here. The protein structure is experimentally determined, the simulation is reasonably long, the data are available through Zenodo, and the authors do acknowledge some of the study’s limitations. The overall sequence of the computational work also makes sense.

Even so, the main claims go well beyond what the results show. Some of the problems are not matters of wording and will require the authors to revisit the analysis and reconsider how the study is framed. For that reason, I recommend Major Revision.

1. My main concern is that the biological mechanism described in the title and abstract does not match the target that was actually modeled. The paper presents the work as an attempt to influence the PlGF and sFlt 1 network. However, the compounds were docked into the intracellular ATP binding site of the VEGFR1 kinase domain. sFlt 1 is a soluble splice form of VEGFR1 that contains the extracellular domains but lacks the intracellular kinase domain. A compound that binds the kinase ATP pocket would not directly change the ability of sFlt 1 to bind PlGF or act as a soluble decoy receptor. The paper would be more accurate if it were presented as a computational investigation of possible inhibition of membrane bound VEGFR1 signaling. The authors should remove the direct sFlt 1 claims unless they can provide a convincing biological explanation for how inhibition of the kinase domain would affect the proposed soluble receptor mechanism.

Response: Thank you for this important mechanistic clarification. We have substantially reframed the manuscript to distinguish membrane-bound VEGFR1 from soluble sFlt-1. The revised text now states that the modeled ATP-binding pocket belongs to the intracellular kinase domain of membrane-bound VEGFR1 and is absent from sFlt-1; therefore, kinase-pocket binding cannot be interpreted as direct modulation of VEGF/PlGF sequestration by sFlt-1. Direct sFlt-1 mechanistic claims were removed, and the title, abstract, discussion, and conclusion now present Emodin-8-glucoside as an exploratory candidate ligand for the VEGFR1 kinase domain rather than as a demonstrated modulator of the PlGF-sFlt-1 axis.

2. The disease rationale is also not yet clear. The manuscript does not show that VEGFR1 kinase activity is increased in placenta previa or that inhibiting this pathway would be beneficial in that condition. Much of the discussion comes from studies of preeclampsia and placenta accreta, which are not the same disease as placenta previa. These conditions should not be discussed as though they have the same underlying biology. The authors need to define the clinical problem more carefully and explain why VEGFR1 kinase inhibition is relevant to placenta previa specifically. This is particularly important during pregnancy, since complete loss of VEGFR1 function is associated with severe disruption of vascular development. At this stage, the work is better described as an exploratory computational study than as evidence for a possible treatment.

Response: Thank you for this comment. The disease rationale has been revised to clearly distinguish placenta previa from placenta accreta spectrum, preeclampsia, and fetal growth restriction. We now explicitly acknowledge that available placenta-previa evidence does not establish increased membrane-bound VEGFR1 kinase activity or demonstrate that VEGFR1 inhibition would be therapeutically beneficial. Accordingly, VEGFR1 is presented as a hypothesis-generating structural target, and the study is consistently framed as an exploratory computational investigation requiring biochemical and placental-model validation rather than evidence for treatment.

3. There are many inconsistencies in the reported values and units. These need to be checked against the original output files. The docking score is given as negative 9.31 kJ per mol in the abstract and negative 9.31 kcal per mol in Table 3. AutoDock Vina reports its scores in kcal per mol. Similar confusion appears in the MOE and MM GBSA results. The HOMO LUMO gap is listed as 0.13204 Hartree in one place, which is about 3.59 eV, but elsewhere it is reported as 0.13204 eV. The LD50 value changes from 5000 to 3000 mg per kg. The Ramachandran percentages appear to add to more than 100 percent, and different sections disagree about whether any residues fall in disallowed regions. The authors should return to the raw files and report one consistent value for each result.

Response: Thank you for identifying these inconsistencies. We rechecked the reported values and standardized the corresponding units and interpretations throughout the manuscript. The AutoDock Vina score for Emodin-8-glucoside is now consistently reported as -9.311 kcal/mol, and MOE values are reported separately according to its scoring function. The HOMO-LUMO gap is consistently reported as 0.13204 Hartree, equivalent to approximately 3.59 eV, and the ProTox 3.0 LD50 is reported as 3000 mg/kg. The PROCHECK results now total 100% (85.5% most favored and 14.5% additionally allowed, with no generously allowed or disallowed residues), while the separate MolProbity assessment is reported independently to avoid mixing validation criteria. MM-GBSA and MM-PBSA results have also been standardized to kcal/mol and are now interpreted as method-dependent end-point energy estimates rather than experimentally measured binding free energies.

4. The docking procedure also needs proper validation. PDB 3HNG contains a crystallographic inhibitor, so the authors should redock that ligand and report the heavy atom RMSD between the experimental pose and the predicted pose. This would provide a basic test of whether the docking setup can reproduce the known binding mode. At present, the results from MOE and Vina do not appear to agree. For example, apigenin 7 O glucoside has a favorable Vina score but a positive MOE value of 22.88. The meaning of that positive value is unclear, and no comparison of the rankings from the two programs is provided. Because of this, the statement that the docking protocol was robust is not supported.

Response: Thank you for this important comment. We validated the docking protocol by redocking the co-crystallized 8ST ligand into the VEGFR1 ATP-binding pocket. DockRMSD yielded a symmetry-corrected heavy-atom RMSD of 1.465 Å relative to the experimental pose, supporting reasonable pose reproduction. The previously inconsistent positive MOE value was rechecked and corrected, and the complete Vina and MOE rankings were compared using Spearman correlation (rho = 0.553, p = 0.005), indicating moderate agreement rather than complete concordance. We have therefore avoided describing the docking protocol as universally robust and interpret the two scoring methods as complementary.

5. The reported Vina RMSD value of 0.00 Å for every compound should also be corrected. That value reflects comparison of the top pose with itself. It is not evidence that the ligand is stable or that the docking result is accurate. The Methods should include the docking box coordinates, exhaustiveness, random seed, software version, charge method, and the protonation and tautomer states used for the protein and ligands.

Response: Thank you for this clarification. The Vina RMSD values of 0.00 Å were removed because they represent self-comparison of the top-ranked pose and do not validate docking accuracy. The Methods now report the docking grid center (x = 7.3 Å, y = 17.8 Å, z = 30.8 Å), a 20 × 20 × 20 Å search box, exhaustiveness of 8, AutoDock Vina Extended v1.2.7 in SAMSON 2026 R1, receptor and ligand charge assignments, nominal pH 7.4, hydrogen treatment, and ligand tautomer handling. No user-defined random seed was specified, and the default stochastic initialization was used. Experimental-pose validation was performed separately by 8ST redocking.

6. The molecular dynamics section is difficult to interpret because only one simulation of one complex was performed. There is no apo protein simulation, no known inhibitor control, and no independent replicate. Without a comparison group, the authors cannot say that the compound stabilized the protein or reduced its flexibility. Those are relative claims and require something to compare against. The lead compound also scored only slightly better than several other compounds in the docking screen. Differences of this size are usually too small to support a claim that one compound clearly outperformed all others.

Response: Thank you for this important point. We added a separate 500 ns simulation of the co-crystallized VEGFR1 ligand 8ST under the same conditions as the Emodin-8-glucoside complex, providing a methodologically consistent control for comparative interpretation. We also revised the MD interpretation so that RMSD, RMSF, PCA, DCCM, and related descriptors are not used to claim ligand-induced receptor stabilization or reduced flexibility. In addition, the docking Results now explicitly acknowledge that Emodin-8-glucoside differed only modestly from several other high-ranking compounds and that these score differences do not establish superior experimental affinity.

7. At minimum, the authors should repeat the simulation using independent initial velocities. A simulation with a known VEGFR1 inhibitor would also make the results much more informative. The full simulation protocol should be reported, including the timestep, treatment of electrostatics, cutoff distances, thermostat, barostat, protein force field, ligand force field, and ligand charge assignment.

Response: We agree that independent replicate simulations would strengthen assessment of convergence and reproducibility. A 500 ns simulation of the co-crystallized 8ST control was added under identical conditions; however, independent replicate trajectories with different initial velocity seeds were not performed, and this is now explicitly acknowledged as a limitation. The full MD protocol has also been expanded to report the ff19SB protein force field, GAFF2 ligand parameters, AM1-BCC ligand charges, 2 fs timestep, particle-mesh Ewald electrostatics, 10 Å nonbonded cutoff, Langevin thermostat, Monte Carlo barostat, NVT/NPT equilibration, and production conditions.

8. The MM PBSA and MM GBSA results raise another serious concern. The paper does not clearly state how many frames were analyzed or how far apart they were taken from the trajectory. Based on the reported standard deviations and standard errors, it appears that the calculation may have used only about ten frames. That would not be enough to represent a 500 ns simulation reliably.

Response: Thank you for identifying this limitation. The revised Methods now explicitly state that MM-GBSA and MM-PBSA calculations used ten snapshots extracted at 50 ns intervals from 50 to 500 ns. We agree that this limited sampling cannot fully represent the conformational ensemble of a 500 ns trajectory. Accordingly, the manuscript now describes these calculations as limited-snapshot, method-dependent end-point energy estimates rather than quantitatively converged binding free energies, and their interpretation has been substantially moderated.

9. The MM PBSA result of negative 1.63 plus or minus 9.70 kcal per mol is consistent with zero. It also does not agree with the value of negative 36.92 plus or minus 3.7 stated in the abstract. The MM GBSA result of negative 43.11 is very different again. These values cannot all be treated as confirmation of strong binding without an explanation. The authors should analyze a larger number of suitably spaced frames and account for correlation between frames when reporting uncertainty. If entropy was not included, the results should be described as endpoint energy estimates rather than absolute binding free energies.

Response: Thank you for this important observation. The MM-GBSA/MM-PBSA section was rechecked and substantially revised. The unsupported MM-PBSA value of -36.92 +/- 3.7 kcal/mol was removed, and the Emodin-8-glucoside values are now consistently reported as -43.11 +/- 4.92 kcal/mol for MM-GBSA and -1.63 +/- 9.70 kcal/mol for MM-PBSA. The near-zero MM-PBSA result is explicitly interpreted as highly variable and not as independent confirmation of favorable binding. We also clarified that ten snapshots at 50 ns intervals were analyzed and that configurational entropy was not included. Accordingly, these values are now described as limited-snapshot, method-dependent end-point energy estimates rather than absolute binding free energies. A larger-frame reanalysis was not performed, and this sampling limitation is acknowledged in the revised manuscript.

10. The hydrogen bond analysis also needs to be checked. An average of about 25 intermolecular hydrogen bonds is unusually high for a small molecule bound to a protein and does not fit well with the eight hydrogen bonds reported from docking. The authors should explain exactly how hydrogen bonds were counted, including the distance and angle criteria. A table showing the occupancy of each ligand protein hydrogen bond would be much more useful than a single average value.

Response: Thank you for this comment. We rechecked the hydrogen-bond analysis and found that the previously reported average of approximately 25 intermolecular hydrogen bonds was not representative of the trajectory data. The revised analysis now shows predominantly approximately 0.5-2.5 intermolecular hydrogen bonds per frame, with occasional transient peaks near 3.5 and a 50-frame rolling mean generally between approximately 0.7 and 2.2. We respectfully note that the eight hydrogen bonds identified from docking and the hydrogen-bond profile obtained from MD are not directly comparable. Docking describes contacts within a single predicted static pose, whereas MD analysis captures continuous formation and disruption of polar contacts across the trajectory. The purpose of the MD analysis is therefore to characterize time-dependent hydrogen-bond behavior rather than residue-specific occupancy. Because the complete time-resolved hydrogen-bond profile is provided graphically, we have retained this trajectory-level representation rather than introducing an additional residue-occupancy table. The interpretation has also been moderated so that hydrogen-bond counts are not treated as independent evidence of binding affinity, inhibitory potency, or complex stability.

11. The ADMET discussion does not match the results in the tables. Table 9 shows high predicted probabilities for mutagenicity and immunotoxicity, along with warnings for kidney, heart, respiratory, and general clinical toxicity. The Discussion nevertheless describes the compound as nonmutagenic and suggests that it has a clean toxicity profile. That interpretation is not supported by the results shown.

Response: Thank you for this comment. The ADMET Results and Discussion have been revised to match the ProTox 3.0 outputs. The manuscript now explicitly reports active predictions for mutagenicity, immunotoxicity, nephrotoxicity, cardiotoxicity, respiratory toxicity, and clinical toxicity, while avoiding descriptions such as "nonmutagenic" or "clean toxicity profile." These computational alerts are now presented as potential liabilities requiring experimental evaluation rather than evidence of safety.

12. SwissADME also predicts low gastrointestinal absorption, P glycoprotein substrate behavior, and a topological polar surface area of about 174. These findings do not suggest favorable absorption. The BOILED Egg figure also appears to have been interpreted incorrectly. Teratogenicity was not evaluated. Since the paper discusses a possible treatment during pregnancy, the safety predictions need to be reported plainly and with appropriate caution.

Response: Thank you for highlighting this issue. The SwissADME interpretation has been revised to explicitly acknowledge the high TPSA of 173.98 Å², low predicted gastrointestinal absorption, P-glycoprotein substrate status, and absence of predicted blood-brain barrier permeation. The BOILED-Egg interpretation and caption were also revised to reflect the SwissADME predictions without claiming favorable absorption. In addition, the manuscript now clearly states that teratogenicity, embryotoxicity, reproductive toxicity, fetal toxicity, placental transfer, and other pregnancy-specific safety endpoints were not evaluated; therefore, no conclusion regarding maternal-fetal safety can be drawn from these predictions.

13. The conclusion should be rewritten to reflect what this type of study can actually establish. Docking and molecular dynamics can help prioritize compounds for further testing, but they do not demonstrate inhibition, selectivity, placental delivery, effectiveness, or safety. The paper should not describe emodin 8 glucoside as a potent, specific, safer, or viable treatment. A more defensible conclusion is that the compound was identified as a computational lead that may be worth testing experimentally.

Response: Thank you for this important comment. The Conclusion has been completely rewritten and the claims have been substantially moderated. Emodin-8-glucoside is now described as an in-silico-prioritized exploratory candidate rather than a potent, selective, safe, or viable treatment. The revised Conclusion explicitly states that the computational results do not establish VEGFR1 inhibition, therapeutic efficacy, selectivity, or pregnancy safety and that experimental validation is required.

14. The next steps would include a VEGFR1 kinase assay with a dose response curve and a known inhibitor as a positive control. A VEGFR2 counter screen would be needed to examine selectivity. Studies in appropriate placental cell models would also be necessary before making any claims about possible therapeutic use.

Response: Thank you for these constructive recommendations. The revised manuscript now proposes a stepwise experimental-validation strategy comprising a biochemical VEGFR1 kinase assay with a dose-response curve and validated inhibitor positive control, a VEGFR2 kinase counter-screen to assess selectivity, and subsequent testing in relevant placental trophoblast and endothelial cell models, including VEGFR1 phosphorylation and downstream functional endpoints.

15. The references should be reviewed one by one. Some do not support the statements attached to them. An EGFR paper is cited for a claim about VEGFR biology, and the reference used for Gaussian appears to be an unrelated colorectal cancer study. The paper should cite the original or standard references for AutoDock Vina, ff19SB, the ligand force field, MMPBSA.py, and Gaussian.

Response: The references and their corresponding in-text citations were reviewed to improve alignment between the cited source and the supported statement. Methodological citations were also revised to prioritize appropriate references for the computational tools and protocols, including molecular docking, molecular dynamics force fields, end-point energy calculations, and Gaussian-based DFT analysis.

16. The manuscript also needs careful language editing. The name of the target is written several different ways, including VEGFR1, VGFR1, and vfr1. Similar inconsistencies appear with PlGF and sFlt 1. Some compound names also change across sections. The description of a steroidal scaffold appears to be incorrect. The reference list contains leftover HTML, and the sentence stating that ciprofloxacin attached favorably to the filter surface is unrelated to this work. The authors should check the rest of the manuscript for similar material.

Response: Thank you for highlighting these language and consistency issues. The manuscript underwent terminology and language revision to standardize VEGFR1, PlGF, sFlt-1, and compound nomenclature. The incorrect steroidal-scaffold description and the unrelated ciprofloxacin sentence were removed, and the manuscript was reviewed for residual text unrelated to the present study.

17. The figures should be regenerated at publication quality, and the source data should be provided. The Methods describe a STRING or protein interaction analysis, but no corresponding result is shown. The authors should either include that analysis in the Results or remove it from the Methods.

Response: Thank you for this comment. The figure set and associated captions were revised for clearer presentation, and the underlying computational data and simulation outputs are available through the publicly accessible Zenodo repository stated in the Data Availability section. The STRING/protein-interaction analysis was removed from the Methods because it was not part of the final reported analysis and therefore had no corresponding Results section.

18. Overall, the study contains a substantial amount of computational work and identifies a compound that may be worth examining further. However, the manuscript currently has major problems with the proposed mechanism, the disease rationale, the docking validation, the lack of molecular dynamics controls, conflicting energy results, and the interpretation of the toxicity predictions. The central biological issue is especially important because a ligand targeting the intracellular VEGFR1 kinase domain cannot directly support the proposed mechanism involving soluble sFlt 1 and PlGF binding. The paper may become suitable for reconsideration after major reanalysis and reframing, but I cannot recommend acceptance in its current form.

Response: Thank you for the comprehensive assessment. We have substantially reanalyzed and reframed the manuscript in response to these concerns. The revised study now distinguishes membrane-bound VEGFR1 kinase signaling from soluble sFlt-1 biology, treats placenta previa separately from preeclampsia and PAS, includes crystallographic-ligand redocking and an 8ST control simulation, moderates interpretation of MD and end-point energy results, corrects the ADMET and toxicity interpretation, and presents Emodin-8-glucoside only as an exploratory computational candidate requiring biochemical and placental-model validation. The title, Abstract, Discussion, and Conclusion have been revised accordingly.

Reviewer #3:

Thank you for the opportunity to review this interesting manuscript. The study presents a comprehensive computational investigation of Emodin-8-glucoside as a potential VEGFR1 inhibitor using an integrated workflow including molecular docking, molecular dynamics simulations, MM-GBSA/MM-PBSA analyses, pharmacophore modeling, density functional theory calculations, ADMET prediction, and protein-protein interaction analysis. The computational workflow is systematic, and the public availability of the datasets enhances the transparency and reproducibility of the study. Overall, the work is technically sound and provides a useful computational basis for future experimental investigations.

I have a few suggestions that could further improve the manuscript:

1. Biological rationale: The Introduction would benefit from a stronger discussion explaining why VEGFR1 inhibition is expected to be therapeutically beneficial in placenta previa. Much of the cited evidence relates to preeclampsia, intrauterine growth restrictio

Response: Thank you for this important suggestion. The Introduction has been substantially revised to clarify the biological rationale and to distinguish placenta previa from preeclampsia, fetal growth restriction, and placenta accreta spectrum. We now explicitly acknowledge that available evidence does not establish increased membrane-bound VEGFR1 kinase activity in placenta previa or demonstrate that VEGFR1 inhibition would be therapeutically beneficial. VEGFR1 is therefore presented as a hypothesis-generating structural target within the angiogenic context of placenta previa, and the study is framed as an exploratory computational investigation rather than evidence of an established therapeutic mechanism. The revised Introduction also distinguishes membrane-bound VEGFR1 kinase signaling from soluble sFlt-1 biology and emphasizes the need for disease-relevant biochemical and placental-model validation.

Note to the Editor: The Reviewer #3 report available to us appears to be incomplete and is truncated after the phrase "intrauterine growth restrictio...". Therefore, we have responded to the portion of the comment that was available in the review file. We would be grateful if the complete Reviewer #3 report could be provided so that we can address any remaining comments in full.

Attachment Submitted filename: Reviewer Response R1.docx

# Peer review: Decision Letter 1

**DOI:** 10.1371/journal.pone.0345201.r003

**Authors:** Yusuf Ayipo

28 Sep 2026

Dear Dr. Ali,

Thank you for submitting your manuscript to PLOS One. After careful consideration, we feel that it has merit but does not fully meet PLOS One’s publication criteria as it currently stands. Therefore, we invite you to submit a revised version of the manuscript that addresses the points raised during the review process.

==============================

**ACADEMIC EDITOR:** Many thanks to the authors for responding positively to the previous concerns. The revision has improved the quality of the submission. However, some grey areas still exist, as highlighted by the reviewer, and these require the authors’ significant attention through another round of revision.

==============================

Please submit your revised manuscript by Nov 12 2026 11:59PM. If you will need more time than this to complete your revisions, please reply to this message or contact the journal office at plosone@plos.org. When you're ready to submit your revision, log on to https://www.editorialmanager.com/pone/ and select the 'Submissions Needing Revision' folder to locate your manuscript file.

- A letter that responds to each point raised by the academic editor and reviewer(s). You should upload this letter as a separate file labeled 'Response to Reviewers'.
- A marked-up copy of your manuscript that highlights changes made to the original version. You should upload this as a separate file labeled 'Revised Manuscript with Track Changes'.
- An unmarked version of your revised paper without tracked changes. You should upload this as a separate file labeled 'Manuscript'.

If you would like to make changes to your financial disclosure, please include your updated statement in your cover letter. Guidelines for resubmitting your figure files are available below the reviewer comments at the end of this letter.

If applicable, we recommend that you deposit your laboratory protocols in protocols.io to enhance the reproducibility of your results. Protocols.io assigns your protocol its own identifier (DOI) so that it can be cited independently in the future. For instructions see: https://journals.plos.org/plosone/s/submission-guidelines#loc-laboratory-protocols. Additionally, PLOS One offers an option for publishing peer-reviewed Lab Protocol articles, which describe protocols hosted on protocols.io. Read more information on sharing protocols at https://plos.org/protocols?utm_medium=editorial-email&utm_source=authorletters&utm_campaign=protocols.

As the corresponding author, your ORCID iD is verified in the submission system and will appear in the published article. PLOS supports the use of ORCID, and we encourage all coauthors to register for an ORCID iD and use it as well. Please encourage your coauthors to verify their ORCID iD within the submission system before final acceptance, as unverified ORCID iDs will not appear in the published article. *Only*  the individual author can complete the verification step; PLOS staff *cannot*  verify ORCID iDs on behalf of authors.

We look forward to receiving your revised manuscript.

Kind regards,

Yusuf Oloruntoyin Ayipo, Ph.D

Academic Editor

PLOS One

Journal Requirements:

1. If the reviewer comments include a recommendation to cite specific previously published works, please review and evaluate these publications to determine whether they are relevant and should be cited. There is no requirement to cite these works unless the editor has indicated otherwise.

2. Please review your reference list to ensure that it is complete and correct. If you have cited papers that have been retracted, please include the rationale for doing so in the manuscript text, or remove these references and replace them with relevant current references. Any changes to the reference list should be mentioned in the rebuttal letter that accompanies your revised manuscript. If you need to cite a retracted article, indicate the article’s retracted status in the References list and also include a citation and full reference for the retraction notice.

**Additional Editor Comments:**

Many thanks to the authors for responding positively to the previous concerns. The revision has improved the quality of the submission. However, some grey areas still exist, as highlighted by the reviewer, and these require the authors’ significant attention through another round of revision.

[Note: HTML markup is below. Please do not edit.]

Reviewers' comments:

Reviewer's Responses to Questions

**Comments to the Author**

Reviewer #1: All comments have been addressed

Reviewer #2: All comments have been addressed

Reviewer #3: All comments have been addressed

**********

2. Is the manuscript technically sound, and do the data support the conclusions?

Reviewer #1: Partly

Reviewer #2: Partly

Reviewer #3: Yes

**********

3. Has the statistical analysis been performed appropriately and rigorously?

Reviewer #1: N/A

Reviewer #2: Yes

Reviewer #3: Yes

**********

4. Have the authors made all data underlying the findings in their manuscript fully available?

The PLOS Data policy

Reviewer #1: Yes

Reviewer #2: Yes

Reviewer #3: Yes

**********

5. Is the manuscript presented in an intelligible fashion and written in standard English?

Reviewer #1: Yes

Reviewer #2: No

Reviewer #3: Yes

**********

Reviewer #1: I have no any other comment because the author has adress all needed information requested previously.

Reviewer #2: I thank the authors for a careful and substantial revision. The three concerns that drove my previous recommendation have been addressed in the analysis itself rather than only in the wording, and the manuscript is much improved. My remaining comments are smaller and can be handled without new calculations.

Technical soundness and support for the conclusions

The reframing of the biological target is now correct. The Introduction (lines 82 to 92) distinguishes membrane bound VEGFR1 from soluble sFlt-1 and states plainly that a ligand occupying the intracellular ATP site would not alter sequestration of VEGF-A or PlGF by sFlt-1. The same distinction is carried through the Methods, Discussion and Conclusion. The disease rationale is also much clearer. The new paragraph at lines 93 to 110 separates placenta previa from placenta accreta spectrum, preeclampsia and fetal growth restriction, and acknowledges that the available evidence does not establish increased VEGFR1 kinase activity in placenta previa. Presenting VEGFR1 as a hypothesis generating structural target is an honest and defensible framing.

The docking protocol is now validated. Redocking of the co-crystallized ligand 8ST gives a symmetry corrected heavy atom RMSD of 1.465 Å, which is a reasonable demonstration that the setup reproduces the known binding mode. The previously unexplained positive MOE value has been corrected, and the two scoring functions are now compared using Spearman rank correlation with the result described as moderate agreement rather than as evidence of robustness. The self comparison RMSD values of 0.00 Å have been removed with an appropriate footnote. The Methods now report the grid center, box dimensions, spacing, exhaustiveness, software version, charge assignment and tautomer handling, which makes the docking reproducible from the text.

The addition of a 500 ns simulation of the VEGFR1 to 8ST complex under identical conditions is the single most valuable change in this revision. It gives the comparative reference that was missing, and the relative claims that previously had no comparator have been removed rather than defended. The statement at line 961 that the analyses cannot establish ligand induced stabilization without apo receptor and replicate simulations is the correct reading of what these data can show.

I also want to acknowledge the handling of the end point energies. The unsupported value has been removed, the remaining values are reported consistently, entropy is stated as excluded, and the calculations are correctly relabeled as end point estimates rather than binding free energies. The authors now report that the co-crystallized control is energetically more favorable than the lead compound under both methods, and they say so directly at lines 962 to 973. Reporting a result that runs against one's own lead compound is good practice and increases my confidence in the revision as a whole.

The ADMET section now matches the underlying tables. The active predictions for mutagenicity, immunotoxicity, nephrotoxicity, cardiotoxicity, respiratory toxicity and clinical toxicity are stated, the earlier language about a clean profile is gone, and the SwissADME findings of high polar surface area, low predicted gastrointestinal absorption and P-glycoprotein substrate status are reported plainly. The explicit statement that teratogenicity, embryotoxicity, reproductive toxicity, fetal toxicity and placental transfer were not evaluated is important given the clinical setting, and I am glad to see it included. The Conclusion has been rewritten appropriately and the proposed experimental path, including a VEGFR1 kinase assay with a positive control, a VEGFR2 counter screen and placental cell models, is sensible.

Statistical analysis

The Spearman rank correlation is the appropriate method for comparing two scoring functions that are not on a common scale, and it is reported with the sample size, the direction convention and the p value. Uncertainties on the energy terms are now reported and, importantly, interpreted. The near zero MM-PBSA value with a large standard deviation is correctly described as uncertain rather than as confirmation of binding. The frame count is now disclosed.

One point remains open. Correlation between frames is still not addressed in the reported uncertainty. In practice the 50 ns spacing makes these snapshots close to independent, so I do not think the standard deviations are seriously optimistic, but ten snapshots is a small sample and no convergence or block analysis is shown. I would also note that the numerical differences between the lead and control energies, 6.41 and 8.35 kcal per mol, are presented without any assessment of whether they are distinguishable given the spread. Since these differences do not favor the lead compound, this omission does not inflate any claim, but a brief statement would be helpful.

Remaining items to address

Hydrogen bond analysis. This is the one item I would ask to be resolved before acceptance. The reported average has changed from approximately 25 intermolecular hydrogen bonds to approximately 0.5 to 2.5 per frame. The new values are physically reasonable and I accept them. However, the distance and angle criteria I asked for are still not given. The Methods at line 378 list protein to ligand hydrogen bonds among the trajectory analyses but define nothing, and no software is named for the trajectory analysis. The caption to Figure 8A refers to the stated hydrogen bond analysis protocol, but no protocol is stated anywhere in the manuscript. Please add the donor to acceptor distance cutoff, the angle criterion and the analysis program. A sentence explaining what produced the earlier value would also be appropriate, since a tenfold change in a reported quantity should be accounted for. I accept the authors' reasoning for presenting the trajectory level profile rather than a residue occupancy table.

Replication and sampling. I accept the authors' decision not to run replicate trajectories, given that the claims have been narrowed accordingly and the limitation is clearly stated at lines 1138 to 1145. If it is convenient, recomputing MM-GBSA and MM-PBSA over the existing trajectories at closer frame spacing would require no additional simulation time and would strengthen this section, but I do not regard it as necessary for acceptance.

References. My previous comment on citation accuracy has only been partly addressed, and one of the specific problems has moved rather than been fixed. a. Reference 10 is a paper on the epidermal growth factor receptor in pregnancy and preeclampsia. It is still cited at line 102 in support of a statement about FLT1 and sFlt-1 expression. Reference 9 supports that statement; reference 10 does not. b. At line 106, reference 53 is a study of phytochemicals in spinocerebellar ataxia type 3 and is cited in support of placenta previa tissue studies. Reference 11 is the appropriate source. The bracket formatting there, "(53) (11)", is also malformed. c. The primary AutoDock Vina reference is still absent. Reference 39 is a tutorial and reference 40 is a colorectal cancer study by the same authors. The original description of the program should be cited. Similarly, reference 48 is a force field benchmarking study rather than the original description of the ligand force field. The references for Gaussian, MMPBSA.py and ff19SB are now correct. d. Reference 3 still contains a leftover HTML entity.

Language and presentation. Several duplications appear to have been introduced during revision. Because PLOS ONE does not copyedit accepted manuscripts, these should be corrected now.

Abstract, lines 40 to 41: a clause is repeated within one sentence, reading "End-point energy calculations were method dependent, end-point energy estimates were method dependent". This text also appears in the abstract field of the submission system and should be corrected in both places.

Discussion, lines 933 to 934: the sentence beginning "The interfacial analyses revealed different interaction patterns" appears twice consecutively.

Methods, lines 297 to 307: the sentence describing placement of the docking grid over Pocket 1 appears twice.

Line 271: "uploaded in verse to the PDB format" appears to be a garbled phrase.

Funding statement, line 1182: refers to "the completion of this review". This is a research article.

Figure 4 caption gives the Vina score as -9.31 while the text and Table 2 give -9.311. Table 2 gives the MOE score as -8.32291 while the text gives -8.3229.

The short title still reads "In Silico Identification of Emodin-8-glucoside as a VEGFR1 Drug Candidate", which is stronger than the revised main title and the revised conclusions. "Candidate ligand" would be consistent.

Because three separate duplications were found, I suggest a full read through for any others.

Data availability. The Zenodo deposit is appropriate and no restrictions are claimed. Please confirm that the archive contains the per snapshot energy values behind the reported means and standard deviations and the raw docking output files, and not only summary tables, since the data points behind means and variance measures fall within the journal's policy.

Figures. The figures are supplied as a Word file. Please ensure that final versions meet the journal's format and resolution requirements.

Recommendation

The central problems I raised previously have been resolved, and the conclusions are now appropriately matched to what a computational study of this kind can support. The remaining items are matters of documentation, citation accuracy and language, none of which require new computation or are likely to alter any finding. I recommend Minor Revision, with the hydrogen bond methods specification treated as a condition of acceptance.

Reviewer #3: (No Response)

**********

what does this mean?). If published, this will include your full peer review and any attached files.

If you choose “no”, your identity will remain anonymous but your review may still be made public.

**Do you want your identity to be public for this peer review?**  For information about this choice, including consent withdrawal, please see our Privacy Policy

Reviewer #1: **Yes:**  Abdullahi Ashimi

Reviewer #2: No

Reviewer #3: **Yes:**  Amina Jafaru Ocheinehi

**********

[NOTE: If reviewer comments were submitted as an attachment file, they will be attached to this email and accessible via the submission site. Please log into your account, locate the manuscript record, and check for the action link "View Attachments". If this link does not appear, there are no attachment files.]

To ensure your figures meet our technical requirements, please review our figure guidelines: https://journals.plos.org/plosone/s/figures

You may also use PLOS’s free figure tool, NAAS, to help you prepare publication quality figures: https://journals.plos.org/plosone/s/figures#loc-tools-for-figure-preparation.

NAAS will assess whether your figures meet our technical requirements by comparing each figure against our figure specifications.

# Author comment: Author response to Decision Letter 2

**DOI:** 10.1371/journal.pone.0345201.r004

30 Sep 2026

We thank the Academic Editor and both reviewers for the time and care they have given to this manuscript across two rounds of review. The comments have been consistently constructive and have led to substantive improvements in the framing of the biological target, the validation and reporting of the computational protocols, the handling of uncertainty in the end-point energy estimates, the accuracy of the reference list and the clarity of the text. We are grateful for the opportunity to submit a further revision. Each comment is reproduced below and followed by our response, and the corresponding revisions have been made in the manuscript and marked so that they can be located readily.

Reviewer #1:

I have no any other comment because the author has addressed all needed information requested previously.

Response: We thank Reviewer 1 for the positive assessment and for the earlier comments, which guided substantial improvements to the analysis and the presentation. No further changes were required in response to this review.

Reviewer #2:

I thank the authors for a careful and substantial revision. The three concerns that drove my previous recommendation have been addressed in the analysis itself rather than only in the wording, and the manuscript is much improved. My remaining comments are smaller and can be handled without new calculations. Technical soundness and support for the conclusions.

The reframing of the biological target is now correct. The Introduction (lines 82 to 92) distinguishes membrane bound VEGFR1 from soluble sFlt-1 and states plainly that a ligand occupying the intracellular ATP site would not alter sequestration of VEGF-A or PlGF by sFlt-1. The same distinction is carried through the Methods, Discussion and Conclusion. The disease rationale is also much clearer. The new paragraph at lines 93 to 110 separates placenta previa from placenta accreta spectrum, preeclampsia and fetal growth restriction, and acknowledges that the available evidence does not establish increased VEGFR1 kinase activity in placenta previa. Presenting VEGFR1 as a hypothesis generating structural target is an honest and defensible framing.

The docking protocol is now validated. Redocking of the co-crystallized ligand 8ST gives a symmetry corrected heavy atom RMSD of 1.465 Å, which is a reasonable demonstration that the setup reproduces the known binding mode. The previously unexplained positive MOE value has been corrected, and the two scoring functions are now compared using Spearman rank correlation with the result described as moderate agreement rather than as evidence of robustness. The self comparison RMSD values of 0.00 Å have been removed with an appropriate footnote. The Methods now report the grid center, box dimensions, spacing, exhaustiveness, software version, charge assignment and tautomer handling, which makes the docking reproducible from the text.

The addition of a 500 ns simulation of the VEGFR1 to 8ST complex under identical conditions is the single most valuable change in this revision. It gives the comparative reference that was missing, and the relative claims that previously had no comparator have been removed rather than defended. The statement at line 961 that the analyses cannot establish ligand induced stabilization without apo receptor and replicate simulations is the correct reading of what these data can show.

I also want to acknowledge the handling of the end point energies. The unsupported value has been removed, the remaining values are reported consistently, entropy is stated as excluded, and the calculations are correctly relabeled as end point estimates rather than binding free energies. The authors now report that the co-crystallized control is energetically more favorable than the lead compound under both methods, and they say so directly at lines 962 to 973. Reporting a result that runs against one's own lead compound is good practice and increases my confidence in the revision as a whole.

The ADMET section now matches the underlying tables. The active predictions for mutagenicity, immunotoxicity, nephrotoxicity, cardiotoxicity, respiratory toxicity and clinical toxicity are stated, the earlier language about a clean profile is gone, and the SwissADME findings of high polar surface area, low predicted gastrointestinal absorption and P-glycoprotein substrate status are reported plainly. The explicit statement that teratogenicity, embryotoxicity, reproductive toxicity, fetal toxicity and placental transfer were not evaluated is important given the clinical setting, and I am glad to see it included. The Conclusion has been rewritten appropriately and the proposed experimental path, including a VEGFR1 kinase assay with a positive control, a VEGFR2 counter screen and placental cell models, is sensible.

Statistical analysis: The Spearman rank correlation is the appropriate method for comparing two scoring functions that are not on a common scale, and it is reported with the sample size, the direction convention and the p value. Uncertainties on the energy terms are now reported and, importantly, interpreted. The near zero MM-PBSA value with a large standard deviation is correctly described as uncertain rather than as confirmation of binding. The frame count is now disclosed.

Response: We are grateful to Reviewer 2 for a detailed and constructive reading of both the original and the revised manuscript, and for acknowledging the changes made to the framing of the biological target, the disease rationale, the docking validation, the addition of the comparative 500 ns simulation of the VEGFR1 to 8ST complex, the reporting of the end-point energies, the ADMET section and the statistical treatment. The remaining points are addressed individually below.

One point remains open. Correlation between frames is still not addressed in the reported uncertainty. In practice the 50 ns spacing makes these snapshots close to independent, so I do not think the standard deviations are seriously optimistic, but ten snapshots is a small sample and no convergence or block analysis is shown. I would also note that the numerical differences between the lead and control energies, 6.41 and 8.35 kcal per mol, are presented without any assessment of whether they are distinguishable given the spread. Since these differences do not favor the lead compound, this omission does not inflate any claim, but a brief statement would be helpful.

Response: We accept this point and have addressed it explicitly rather than by implication. The Methods now state that the ten snapshots used for the end-point calculations were taken at 50 ns intervals between 50 and 500 ns, that correlation between snapshots at this spacing is expected to be limited, and that no block averaging or formal convergence assessment was performed; the reported means, standard deviations and standard errors are accordingly presented as descriptive of the ten sampled snapshots rather than as converged estimates of the underlying distributions. The Results now describe the differences of 6.41 and 8.35 kcal/mol between the lead compound and the co-crystallized control as not formally distinguishable given the standard deviations and the small number of snapshots, and the limitations paragraph repeats that a single 500 ns trajectory per complex was run, without replicates, and that no block averaging or convergence analysis was carried out. Consistent with the reviewer's observation that these differences do not favor the lead compound, no inference is drawn from their magnitude.

Remaining items to address

Hydrogen bond analysis. This is the one item I would ask to be resolved before acceptance. The reported average has changed from approximately 25 intermolecular hydrogen bonds to approximately 0.5 to 2.5 per frame. The new values are physically reasonable and I accept them. However, the distance and angle criteria I asked for are still not given. The Methods at line 378 list protein to ligand hydrogen bonds among the trajectory analyses but define nothing, and no software is named for the trajectory analysis. The caption to Figure 8A refers to the stated hydrogen bond analysis protocol, but no protocol is stated anywhere in the manuscript. Please add the donor to acceptor distance cutoff, the angle criterion and the analysis program. A sentence explaining what produced the earlier value would also be appropriate, since a tenfold change in a reported quantity should be accounted for. I accept the authors' reasoning for presenting the trajectory level profile rather than a residue occupancy table.

Response: We apologize for leaving this unspecified and have now stated the protocol in full. The Methods report that all trajectory analyses, including the receptor to ligand hydrogen bond analysis, were performed with the CPPTRAJ module of AmberTools25, cited as reference 52, and that a hydrogen bond was counted when the donor to acceptor heavy-atom distance was at or below 3.0 Å and the donor to hydrogen to acceptor angle was at or above 135°, with the search restricted to direct intermolecular receptor to ligand donor and acceptor pairs so that intramolecular hydrogen bonds and water-bridged contacts are excluded. A sentence has also been added accounting for the change in the reported average, which arose because the earlier count was not restricted to direct intermolecular pairs and consequently aggregated intramolecular and water-mediated contacts; the recomputed values obtained under the criteria above supersede it. The captions to Figures 8 and 11 now refer to the hydrogen bond distance and angle criteria stated in the Methods rather than to an unstated protocol. We thank the reviewer for accepting the trajectory-level profile in place of a residue occupancy table.

Replication and sampling. I accept the authors' decision not to run replicate trajectories, given that the claims have been narrowed accordingly and the limitation is clearly stated at lines 1138 to 1145. If it is convenient, recomputing MM-GBSA and MM-PBSA over the existing trajectories at closer frame spacing would require no additional simulation time and would strengthen this section, but I do not regard it as necessary for acceptance.

Response: We thank the reviewer for accepting this decision. The claims remain narrowed accordingly, and the limitations paragraph continues to state that a single 500 ns trajectory was run per complex, that no replicate trajectories were performed, and that the end-point estimates rest on ten snapshots without block averaging or a formal convergence assessment. We have not recomputed the end-point energies at closer frame spacing for this revision. So that this can be pursued directly by any interested reader, the per-snapshot energy values behind the reported means, standard deviations and standard errors are included in the deposited archive.

References. My previous comment on citation accuracy has only been partly addressed, and one of the specific problems has moved rather than been fixed. a. Reference 10 is a paper on the epidermal growth factor receptor in pregnancy and preeclampsia. It is still cited at line 102 in support of a statement about FLT1 and sFlt-1 expression. Reference 9 supports that statement; reference 10 does not. b. At line 106, reference 53 is a study of phytochemicals in spinocerebellar ataxia type 3 and is cited in support of placenta previa tissue studies. Reference 11 is the appropriate source. The bracket formatting there, "(53) (11)", is also malformed. c. The primary AutoDock Vina reference is still absent. Reference 39 is a tutorial and reference 40 is a colorectal cancer study by the same authors. The original description of the program should be cited. Similarly, reference 48 is a force field benchmarking study rather than the original description of the ligand force field. The references for Gaussian, MMPBSA.py and ff19SB are now correct. d. Reference 3 still contains a leftover HTML entity.

Response: We are grateful for the continued attention to the reference list. The incorrect reference has been removed from the statement on FLT1 and sFlt-1 expression, which now cites references 8 and 9. The malformed bracket in the sentence on "pernicious" placenta previa has been replaced by a single, correct citation to the biomarker source, reference 1. The primary description of AutoDock Vina by Trott and Olson, published in 2010, has been added as reference 40 alongside the earlier references, so that references 38 to 40 are now cited together for the docking program. The original description of the general ligand force field by Wang and co-workers, published in 2004, is now cited as reference 48 in place of the force field benchmarking study, and the trajectory analysis program is cited as reference 52, alongside the existing citations for the semi-empirical charge model and the end-point energy implementation at references 47 and 53. The stray character in reference 3 has been removed and the reference list has been regenerated. Each of these citation changes is marked in the revised file so that it can be located readily.

Language and presentation. Several duplications appear to have been introduced during revision. Because PLOS ONE does not copyedit accepted manuscripts, these should be corrected now.

Abstract, lines 40 to 41: a clause is repeated within one sentence, reading "End-point energy calculations were method dependent, end-point energy estimates were method dependent". This text also appears in the abstract field of the submission system and should be corrected in both places.

Discussion, lines 933 to 934: the sentence beginning "The interfacial analyses revealed different interaction patterns" appears twice consecutively.

Methods, lines 297 to 307: the sentence describing placement of the docking grid over Pocket 1 appears twice.

Line 271: "uploaded in verse to the PDB format" appears to be a garbled phrase.

Funding statement, line 1182: refers to "the completion of this review". This is a research article.

Figure 4 caption gives the Vina score as -9.31 while the text and Table 2 give -9.311. Table 2 gives the MOE score as -8.32291 while the text gives -8.3229.

The short title still reads "In Silico Identification of Emodin-8-glucoside as a VEGFR1 Drug Candidate", which is stronger than the revised main title and the revised conclusions. "Candidate ligand" would be consistent.

Because three separate duplications were found, I suggest a full read through for any others.

Response: We apologize for these duplications, which were introduced while consolidating text during the previous revision, and all have been removed. The repeated clause in the Abstract has been deleted, and the corrected abstract will be entered in the submission system so that the two versions agree. The duplicated sentence in the Discussion concerning the interfacial interaction patterns and the duplicated sentence in the Methods describing placement of the docking grid over Pocket 1 have each been reduced to a single occurrence. The garbled phrase in the Methods has been rewritten and now reads that the curated VEGFR1 model was uploaded in PDB format. The funding statement has been corrected and now refers to a research article. The docking scores are reported consistently, with the Vina score for Emodin-8-glucoside given as -9.311 kcal/mol in the Results text and in the Figure 4 caption and the MOE GBVI/WSA score given as -8.3229 in the Results text and in Table 2; Figure 4 is now also cited in the Results text. The short title flagged by the reviewer does not appear in the manuscript file and exists only in the submission system; it has been changed there to read candidate ligand rather than drug candidate, so that it is consistent with the revised main title and the revised conclusions. Following the reviewer's suggestion, we have read the whole manuscript through once more for any further duplications.

Data availability. The Zenodo deposit is appropriate and no restrictions are claimed. Please confirm that the archive contains the per snapshot energy values behind the reported means and standard deviations and the raw docking output files, and not only summary tables, since the data points behind means and variance measures fall within the journal's policy.

Response: We confirm that the public Zenodo record contains the per-snapshot MM-GBSA and MM-PBSA energy values underlying every reported mean, standard deviation and standard error, together with the raw docking output files, so that the individual data points behind the reported means and variance measures are available rather than summary tables alone. The record, which is available at https://doi.org/10.5281/zenodo.17528796, also holds the ligand structure files in SDF and PDB format, the receptor structure files, the active-site prediction output and the density functional theory output, and the manuscript's Data Availability statement lists these datasets together with the deposited video of the molecular dynamics trajectories. One limitation should be stated openly rather than left for the reviewer to infer. Each simulated complex was run for 500 ns, and the raw trajectory files therefore amount to several gigabytes. What the authors currently have deposited is the video record of those trajectories; the full raw trajectory files exceeded the upload capacity available at the time of resubmission, a temporary internet connectivity limitation at the authors' location, and they are consequently not yet in the repository. We will add the complete trajectory files to the same Zenodo record once upload becomes possible, and in the meantime we can supply them directly to the editorial office or to the reviewer on request.

Figures. The figures are supplied as a Word file. Please ensure that final versions meet the journal's format and resolution requirements.

Response: We confirm that the final figures will be supplied as individual files meeting the journal's format and resolution requirements, rather than embedded in a Word document.

Recommendation

The central problems I raised previously have been resolved, and the conclusions are now appropriately matched to what a computational study of this kind can support. The remaining items are matters of documentation, citation accuracy and language, none of which require new computation or are likely to alter any finding. I recommend Minor Revision, with the hydrogen bond methods specification treated as a condition of acceptance.

Response: We thank Reviewer 2 for the careful evaluation across both rounds and for the recommendation. The hydrogen bond methods specification identified as a condition of acceptance has been implemented in full, with the distance and angle criteria, the restriction to direct intermolecular receptor to ligand pairs and the analysis program all stated in the Methods, and the remaining items of documentation, citation accuracy and language have been addressed as described above. No finding has changed as a result of these revisions.

Attachment Submitted filename: Reviewer Response R2.docx

# Editor report: Decision Letter 2

**DOI:** 10.1371/journal.pone.0345201.r005

**Authors:** Yusuf Ayipo

6 Oct 2026

Structure-Based Computational Prioritization of Emodin-8-glucoside as a Candidate Ligand for the VEGFR1 Kinase Domain in the Context of Placenta Previa

PONE-D-26-09825R2

Dear Dr. Ali,

We’re pleased to inform you that your manuscript has been judged scientifically suitable for publication and will be formally accepted for publication once it meets all outstanding technical requirements.

Within one week, you’ll receive an e-mail detailing the required amendments. When these have been addressed, you’ll receive a formal acceptance letter and your manuscript will be scheduled for publication.

An invoice will be generated when your article is formally accepted. Please note, if your institution has a publishing partnership with PLOS and your article meets the relevant criteria, all or part of your publication costs will be covered. Please make sure your user information is up-to-date by logging into Editorial Manager at Editorial Manager® and clicking the ‘Update My Information' link at the top of the page. For questions related to billing, please contact billing support.

If your institution or institutions have a press office, please notify them about your upcoming paper to help maximize its impact. If they’ll be preparing press materials, please inform our press team as soon as possible -- no later than 48 hours after receiving the formal acceptance. Your manuscript will remain under strict press embargo until 2 pm Eastern Time on the date of publication. For more information, please contact onepress@plos.org.

Kind regards,

Yusuf Oloruntoyin Ayipo, Ph.D

Academic Editor

PLOS One

Additional Editor Comments (optional):

The submission is scientifically sound for publication in this title, and all the concerns raised by the respective reviewers regarding the manuscript quality have been satisfactorily addressed. I hereby recommend the manuscript for publication in the current version.

Reviewers' comments:

# Editor report: Acceptance letter

**DOI:** 10.1371/journal.pone.0345201.r006

**Authors:** Yusuf Ayipo

PONE-D-26-09825R2

PLOS One

Dear Dr. Ali,

I'm pleased to inform you that your manuscript has been deemed suitable for publication in PLOS One. Congratulations! Your manuscript is now being handed over to our production team.

At this stage, our production department will prepare your paper for publication. This includes ensuring the following:

* All references, tables, and figures are properly cited

* All relevant supporting information is included in the manuscript submission,

* There are no issues that prevent the paper from being properly typeset

You will receive further instructions from the production team, including instructions on how to review your proof when it is ready. Please keep in mind that we are working through a large volume of accepted articles, so please give us a few days to review your paper and let you know the next and final steps.

Lastly, if your institution or institutions have a press office, please let them know about your upcoming paper now to help maximize its impact. If they'll be preparing press materials, please inform our press team within the next 48 hours. Your manuscript will remain under strict press embargo until 2 pm Eastern Time on the date of publication. For more information, please contact onepress@plos.org.

You will receive an invoice from PLOS for your publication fee after your manuscript has reached the completed accept phase. If you receive an email requesting payment before acceptance or for any other service, this may be a phishing scheme. Learn how to identify phishing emails and protect your accounts at https://explore.plos.org/phishing.

If we can help with anything else, please email us at customercare@plos.org.

Thank you for submitting your work to PLOS One and supporting open access.

Kind regards,

PLOS One Editorial Office Staff

on behalf of

Dr. Yusuf Oloruntoyin Ayipo

Academic Editor

PLOS One
